# Advancing outcome measure development and analytical approaches: Pain in Animals Workshop 2023

**DOI:** 10.3389/fpain.2025.1615862

**Published:** 2025-08-21

**Authors:** B. D. X. Lascelles, D. Barratt, P. S. Basran, D. C. Brown, J. F. Coetzee, M. Gill, M. R. Hutchinson, C. Johnson, S. P. L. Luna, D. P. Mohapatra, M. L. Oshinsky, S. Robertson, C. F. Ruberman, E. R. Smith, Q. Zhang

**Affiliations:** ^1^ Translational Research in Pain and Comparative Pain Research and Education Center, Department of Clinical Sciences, College of Veterinary Medicine, North Carolina State University, Raleigh, NC, United States; ^2^ Thurston Arthritis Center, UNC School of Medicine, Chapel Hill, NC, United States; ^3^ Center for Translational Pain Research, Department of Anesthesiology, Duke University, Durham, NC, United States; ^4^ School of Biomedicine, University of Adelaide, Adelaide, SA, Australia; ^5^ College of Veterinary Medicine, Cornell University, Ithaca, NY, United States; ^6^ Medical Affairs, Mars Veterinary Health, Vancouver, WA, United States; ^7^ Department of Anatomy and Physiology, Kansas State University, Manhattan, KS, United States; ^8^ National Institute of Neurological Disorders and Stroke/National Institutes of Health, Bethesda, MD, United States; ^9^ Center for Veterinary Medicine, Food and Drug Administration, Rockville, MD, United States; ^10^ Faculty of Veterinary Medicine and Animal Science, Sao Paulo State University, Botucatu, Brazil; ^11^ Lap of Love Veterinary Hospice, Lutz, FL, United States

**Keywords:** pain, Pain in Animals Workshop, translational, measurement, biomarker

## Abstract

Annually, millions of humans and animals suffer from chronic and acute pain, creating welfare and quality of life concerns for both humans and animals who suffer this pain. In developing new therapeutic approaches, the challenge is to accurately measure this pain to ascertain the efficacy of novel therapeutics. Additionally, there is a need to develop new and effective analgesic options that may offer alternatives to using opioids that contribute to the opioid epidemic. The Pain in Animals Workshop (PAW) meetings are held every other year in partnership with the National Institutes of Health (NIH), bringing key stakeholders together to understand pain in humans and animals better. The 2023 workshop focused on presenting and discussing updates on validated approaches to measuring pain, highlighting opportunity areas for additional outcome measure development. It also discussed study design and analytic approaches to the use of outcome measures in clinical trials, including the important concepts of success-failure approaches and the application of multiple endpoints in evaluating analgesic therapies. The workshop also introduced the concept of the biopsychosocial model of pain, broadening the conversation around the impact of pain and thus opportunities to modulate the pain experience. The application of artificial intelligence to the measurement of pain was introduced. The workshop brought together academia, government, and industry experts in human and animal pain assessment and analgesic intervention development. Given the topic's importance and the meeting's uniqueness, capturing the thoughts and ideas presented and discussed is critical. This narrative is one product from that meeting, summarizing several presentations from the workshop.

## Introduction

The 2-day Pain in Animals Workshop was held on September 26 and 27 2023, at the National Institutes of Health campus, Bethesda, Maryland. The full agenda can be found in Supplementary File S1. On the first day, the morning covered the topic of “Updates on validated approaches to measuring pain”, focusing on new information available since the 2017 and 2019 PAW meeting. The day began with the first annual Michele Sharkey Memorial Lecture (narrative summary submitted for publication), followed by discussions on the measurement properties of pain scoring instruments in farm animals, pain biomarkers, and the application of artificial intelligence/machine learning to large animal pain assessment. The afternoon focused on “Opportunity Areas (Biopsychosocial) for Additional Outcome Measure Development”, with presentations covering the biopsychosocial model of pain and discussions on the domains impacted by acute and chronic pain. The second day focused on study design and analytic approaches to using outcome measures in clinical trials, including defining clinically meaningful changes and success/failure criteria for outcome measures, as well as using composite endpoints and adaptive study designs. This narrative contains summaries from speakers who were able to contribute extended abstracts to this document, and the remaining presentation abstracts can be found in the meeting proceedings (Supplementary File S2). This narrative is complementary to the recordings of the workshop, which are archived at the NIH and are available at:

Day 1, https://videocast.nih.gov/watch=52472;
Day 2, https://videocast.nih.gov/watch=52508

## Updates on validated approaches to measuring pain

The morning session of the first day was devoted to discussing recent updates to current approaches to measuring pain, and then extending the discussion by focusing on where the field is moving. The Plenary Lecture by Dottie Brown “Outcome Assessment in Veterinary Pain Studies: The Yellow Brick Road Continues” provided historical background to, and the current status of, the use of owner completed questionnaires to assess the impact of chronic osteoarthritis pain in dogs (1). This also provided a reflection on how the PAW forums have been integral to the most recent wave of knowledge gain and awareness. Here, two other presentations highlighting the future direction of research are summarized: the application of biomarkers, and artificial intelligence.

## Biomarker update: what progress have we made?

In this presentation, Daniel Barratt discussed the past, present, and future of the discovery and development of blood-based pain biomarkers in livestock, with circulating miRNAs as the current focus.

### Contexts of use for objective biomarkers of pain in livestock

Several main contexts of use currently motivate and guide efforts to discover, develop, and validate objective blood-based pain biomarkers in livestock. Primarily, there is a need for tools to objectively measure pain within clinical research and trial settings to demonstrate analgesic efficacy in support of drug development and approvals in livestock species (2, 3). Within this context, it is expected that convergent evidence from multiple endpoints will be required to demonstrate the efficacy of a pain mitigation intervention. Therefore, whilst there is aspiration toward surrogate endpoint status, novel objective measures will likely be employed as supportive diagnostic or monitoring biomarkers in a multidimensional assessment (2–6).

### Psychoneuroimmunological basis for blood-based pain biomarkers

Increased understanding of the immune system's role in pain and the bidirectional communication between central and peripheral neuronal and immune systems has prompted the exploration of novel blood-based transcriptomic biomarkers of pain and analgesia (7–9). Examples of the potential for blood-based biomarkers of pain are the work identifying blood transcriptome changes that correlate strongly with variability in graded chronic constriction injury-induced pain in rats (10), as well as human research showing blood cell immune phenotyping can differentiate between painful patients and healthy controls (11), and also blood transcriptome changes that could differentiate between high and low pain states within an individual, as well as between painful patients and healthy controls (12).

### Circulating miRNAs as potential pain biomarkers

MicroRNAs are small, non-coding RNA molecules that regulate gene expression within and between cells through release into the extracellular environment (e.g., within exosomes). Circulating microRNAs can be quantified in blood plasma and serum and have shown potential as biomarkers for various pathological conditions (including neurological disorders) (13, 14). Thus, circulating microRNAs have attracted interest as possible pain biomarkers in veterinary species. Lecchi and colleagues identified candidate microRNAs differentially expressed between sham versus tail docked and castrated piglets, between horses with acute laminitis vs. controls, and between pre- vs. post-surgery in pond sliders (15–17).

### What progress have we made?

Promising results have been seen in a Kansas State University and University of Adelaide collaboration to identify candidate microRNA biomarkers of pain and analgesia utilizing existing behavioral data and small RNA sequencing of serum exosomes from analgesic trials. In male Holstein calves undergoing sham procedure, or dehorning and castration with or without analgesia (meloxicam ± lidocaine), repeated measures differential gene expression analysis was combined with binomial and linear regression approaches, applying the principles of matching analysis method to the context of use, and harnessing the power of repeated measures and variability within animals and treatment groups. This approach successfully identified serum exosome miRNAs with temporal expression profiles closely matching those of behavioral pain scores, as well as “rapid” (6 h post-procedure) and “delayed” (96+ hours post-procedure) response miRNAs. Lead candidates, such as bta-miR-30a-5p, −122, −143 and −885, demonstrated “outstanding” to “perfect” performance (areas under receiver operating characteristic curves (AUROC) >0.9) in distinguishing sham from dehorned and castrated calves, exceeding other measures reported previously (6), as well as indicating “reversal” with meloxicam treatment (to which other trial measures were insensitive). Similar promising results from a trial of firocoxib and ethyl chloride in piglets undergoing tail docking (and castration for males) were also presented. However, caution was advised due to the absence of a sham control (no docking/castration) in the piglet trial.

### Next steps and key challenges

Currently, at the discovery stage of biomarker development, these findings need to be replicated with appropriate controls. Two key limitations of work to date and challenges to moving forward for qualifying transcriptomic blood-based biomarkers for pain were discussed. Firstly, variations in sample type (e.g., whole blood, plasma, serum, exosomes) and processing, sequencing methodology, and bioinformatic and analysis decisions post-sequencing create challenges for assessing reproducibility and, thus, candidate prioritization across multiple studies. Secondly, trials employing standard husbandry procedures and veterinary-relevant analgesic approaches (albeit with uncertain analgesic efficacy) have high ecological validity. Still, they are ill-suited for distinguishing pain biomarkers *per se* from tissue damage and wound healing biomarkers. Bioinformatic (e.g., gene set/pathway enrichment) analyses, when implemented appropriately (18) may provide tenuous support for a mechanistic link to pain. However, suppose blood-based pain biomarkers are to achieve surrogate endpoint status. In that case, complementary studies using injury-free pain models and/or analgesic (or even anesthetic) approaches of more certain efficacy may be required to demonstrate specificity.

Future research should integrate these promising biomarker approaches alongside end-of-life brain and spinal cord histology and protein analyses to build an integrated picture of the short- and long-term molecular, cellular, systems, and behavioral consequences of pain associated with procedures, paving the way for significant improvements in the diagnosis and treatment of pain in livestock.

## Artificial intelligence applied to measuring pain

Parminder Basran gave an overview of artificial intelligence (AI) in veterinary medicine and touched on the more recent application of AI to pain measurement.

Artificial intelligence (AI) is revolutionizing the way we live and work. The reach of AI systems seems ubiquitous, including search tools, recommender systems, personal assistants, fraud detection, and automated systems. The impact of AI in veterinary medicine is similarly growing, and it has the potential to become an essential tool for companion animal health, livestock health, and population medicine (19). With the advent of AI, veterinarians will have access to powerful algorithms and machine-learning tools that may help them make timely and potentially more accurate diagnoses (20). AI can also analyze large volumes of data, including medical records, diagnostic tests, and imaging studies, to identify patterns and trends, which may enable veterinarians to provide more personalized and effective treatment plans for their patients (21). By collecting and analyzing data from multiple sources, including animal health records, environmental data, and social media; AI can help identify disease outbreaks and inform public health policies (22). This can be especially helpful in managing zoonotic disease outbreaks that can spread from animals to humans and vice versa (23). AI is also transforming agricultural practices where farmers can monitor the health and well-being of their livestock, identify potential health issues early on, and optimize feeding and breeding practices (24). This can improve animal welfare and increase productivity, benefiting farmers and consumers.

AI may be broadly categorized as computer vision, natural language processing, and speech recognition tasks (25). Applications of these forms of AI have only recently emerged in animal behavior and pain assessments (26–28). While all AI applications are exciting and can potentially improve animal health and welfare, they also raise ethical concerns about data privacy, transparency challenges, and bias. In the context of pain assessment, some additional issues to address include:

1. Clarity and transparency of AI features and decisions: The ability to understand the features learned by AI methods and the comprehensibility of their decision-making processes are pivotal for these systems to be practical and accepted in clinical settings as supportive tools for pain diagnosis and monitoring. This is particularly important in veterinary medicine, where there is heavy reliance on signalment and visual assessments of animals to deduce states of pain. A significant hurdle to overcome in applying any form of AI to assist with the identification of pain is ensuring the “ground truth” of the data the algorithm is trained on is the best it can be.
2. Leveraging multiple data sources for enhanced performance: Incorporating a wide range of pain-related data, alongside integrating additional functions such as detecting head pose, motion, and facial features, can enhance the resilience and effectiveness of automatic pain detection systems. Acquiring multiple data sources and integrating them for analysis poses challenges in the veterinary setting, given the often less than optimal environmental conditions.
3. Addressing challenges through interdisciplinary collaboration: Tackling the difficulties in developing a reliable automatic pain detection system requires collaborative efforts across various disciplines. The success of AI adoption heavily depends on the spectrum of disciplines involved in its development and adoption (29). Collaboration amongst veterinarians, basic biologists, data scientists, and other specialties will ensure that AI models applied to pain assessment are adaptable and adoptable.
4. Incorporating temporal aspects and medication effects: Considering the time-based attributes of pain episodes and the influence of pain medication can be a valuable approach for reducing false alarms and enhancing the precision of pain detection.
5. Exploring generalizability across diverse cohorts: Investigating the capacity of automatic pain detection systems to perform consistently across different groups with varying diagnostic statuses should be a subject of future research. When faced with significant genotype and phenotype variations, generalizability becomes a sharper challenge for animals. Models must be characterized with a “Range of Usefulness” based on breeds, sex, and other potential covariates.
6. Address ethical considerations: AI models rely on reliable and reproducible datasets for training. The generation of data for training AI models that classify behavior or quantify pain in animals should be done ethically and responsibly. Animal owners and caregivers may also be integral participants in data collection and utilization.

These considerations underline a need for transparent and responsible data management practices and emphasize an imperative to improve the AI competencies of researchers and practitioners engaged in pain research. The application of AI technologies should not be perceived as an all-encompassing solution for pain evaluation and assessment but instead as a collection of tools that can offer decision support for healthcare professionals.

## Overview of the domains impacted by pain

As an introduction to the afternoon session on “Opportunity Areas (Biopsychosocial) for Additional Outcome Measure Development”, Duncan Lascelles provided an overview of the domains/dimensions impacted by pain. The presentation aimed to broaden the perspective on the potential changes that could be measured as a surrogate estimation of the impact of pain in different species.

The backbone of pain assessment in humans is self-report. In non-verbal humans and animals, self-report is not an option, and thus, one might consider the measurement of pain to be an uphill struggle, especially when one considers that we cannot measure pain in an animal or human who does not self-report—pain is what the individual says it is and describes it as. Indeed, without the option of self-report of pain, the measurement of pain in any species is difficult. Still, on the positive side, pain affects individuals in a multidimensional manner, resulting in many different ways in which the impact of pain can be measured. Measuring the impact of pain allows us to estimate pain. Pain has both neurophysiologic and affective components, which means that a variety of aspects (or domains) of feeling, behavior, function, and social interactions are affected. This multidimensional impact on people includes cognitive, affective, behavioral, functional, physiological, sensory, and socio-cultural dimensions or domains (30). The multi-dimensionality of pain offers a multitude of opportunities to measure the impact of pain and hence estimate the burden of “pain” itself.

There is no “gold standard” consensus on describing the domains impacted by acute or persistent (chronic) pain. Descriptions of the domains impacted by pain vary depending on the pain condition and the context in which they are being discussed (31). There is further variation depending on whether domains that contribute to quality of life/health are being described (in reference to the impact pain has on health), or whether the focus is more directly on what behaviors that pain impacts. Further, the descriptions of domains impacted by pain can be viewed from the perspective of the available measurement tools. Regardless of the way in which one describes “domains” in relation to pain, the fact is that pain has widespread and varied impacts across all aspects of an animal's life. This provides an opportunity to develop multiple measurement tools and, by extension, is the chance to gain a more holistic view of the negative impact of pain or the utility of an analgesic intervention.

A starting point for consideration of the broad multidimensional impact of pain might be to consider the following domains:

– Movement and mobility
– Ability to perform the activities of daily living
– Cognitive function
– Affective states (fear, anxiety)
– Interactions with conspecifics, other animals and humans (social)
– Physiological function
– Sensory processing
– Sleep

Such a list can be used to explore varied and new ways to approach the measurement of the impact of pain. For example, in canine osteoarthritis (OA), there has been an emphasis on the measurement of limb use and function (32). However, assessing other domains, such as cognitive function and sleep quality, may provide meaningful insight into the impact of pain.

When one considers the domains that are impacted by pain, it is important to understand that within each domain are many varying aspects that can be affected—and so opportunities for measurement. For example, canine osteoarthritis pain clearly impacts the domain of “movement and mobility”. But within this, it is understood that there are multiple components that can be differentially impacted by joint pain:

• Limb use
• Overall activity
• Smoothness or quality of motion
• Power of movement
• Resting body weight distribution
• Speed of motion
• Willingness to move

Even within each of these are multiple measures—for example, with “limb use”, one can measure vertical force, vertical impulse, propulsion and braking forces, and static loading. Each of these can be further broken down into summary values (e.g., vertical force can be summarized as “peak vertical force”, or described as a force/time curve. It is also important to remember that within one domain, pain can differentially impact components. Think, for example, of a dog with significant OA pain in one joint; the use of that limb would likely be dramatically negatively affected. Still, overall mobility (effected by using the other 3 limbs) may not be greatly affected much.

A comprehensive understanding of the domains and their components that are impacted by different pain states and conditions will lead to the development of new measurement approaches and tools. A challenge will be to understand how pain impacts different individuals within a pain condition and, therefore, the importance of measuring one domain, or one aspect of a particular domain, in both individual and groups of animals. A further challenge will be to understand what impacts are meaningful to the individual animal and what a clinically important change in the measured parameter is. There has been discussion in the veterinary literature about what degree of change in a measured parameter is unlikely to be seen by chance, and so, by extension, what degree of change may be meaningful (e.g., peak vertical force 32), but only recently have focused attempts have been made to start to define “minimal clinically important differences” for the measurement tools currently in use (33).

## The biospychosocial model of pain

Mark Hutchinson elucidated the complexities of pain as a biopsychosocial phenomenon, using livestock as the example. The presentation underscored the interplay between biological processes and environmental factors, positing pain not merely as a physical sensation but as a complex puzzle that requires multidimensional analysis and innovative methodologies to be fully understood.

### Pain as a complex biopsychosocial puzzle

Pain in animals should be redefined as a complex biopsychosocial puzzle. This perspective recognizes pain as more than a simple response to physical stimuli; it involves a dynamic interaction among biological, psychological, and social factors. Traditional models, which predominantly focus on the physiological aspects of pain, fail to capture the nuanced realities experienced by animals. This broader framework necessitates a shift from a unidimensional to a holistic approach, where pain assessment incorporates behavioral changes, environmental contexts, and psychosocial dynamics (8).

### Focusing on psychoneuroimmunology

Psychoneuroimmunology explores how the nervous and immune systems interact within the context of pain. Stress and disease can modify immune responses, which, in turn, impact neurological states, affecting an animal's pain perception and behavior. Studies have revealed that by understanding these interactions, particularly how they manifest in chronic pain states, new therapeutic strategies can be devised that are more aligned with the underlying psychobiological mechanisms of pain rather than merely addressing its symptoms (9).

### Window into biopsychosocial pain

Advanced analytical technologies can be used to provide a window into the biopsychosocial aspects of pain. The use of biophotonics in this context is pivotal, offering real-time insights into the physiological changes occurring within an animal subjected to various stressors. These insights may lead to the development of timely, precise, and context-specific interventions, ultimately leading to improved animal welfare and management practices (34, 35). Using spectral domain analysis, early work shows how features in the central nervous system of animals change proportionally with their pain states, providing a vivid illustration of how pain impacts neurological functions (34–36).

Assuming advances are to be made in the field. In that case, it has to be understood that significant shortcomings are associated with viewing pain as a digital signal (37) — a binary state of “pain” or “no pain” — which oversimplifies the true nature of pain processes. By treating pain as an analogue signal, researchers and clinicians can capture a continuum of pain intensities and complexities, enhancing the accuracy of pain assessments and the effectiveness of interventions. Further, pain should be explored from the perspective of measurements of real-time relevance, utilizing techniques that provide immediate, actionable data on an animal's pain state. This approach is critical in understanding and managing pain as it unfolds, rather than relying solely on retrospective or less timely data. It shifts the focus from static to dynamic pain assessment, facilitating interventions that are responsive to the immediate needs of the animal. Or even better, prevent the conversion of acute to chronic pain states. To achieve all of this, understanding and measuring changes in the foundational neurobiological substrate of nociceptive processing is essential for developing validated, evidence-based practices in pain management (9). The future of successful pain research lies in its ability to harness cutting-edge or innovative technologies to observe, in real-time, how pain modifies the central nervous system's activity and to develop interventions that directly address these changes.

The translation of research findings into practice is a cornerstone of the future of the work in the field. There must be an emphasis on the importance of convergence in research practices—integrating insights from various disciplines to ensure that scientific advancements have practical and translational relevance (7).

Future research will see the formation of large convergence science teams that operate beyond the sum of their parts. The evolving geopolitical landscape, such as the AUKUS agreement (trilateral security partnership between Australia, U.K. and U.S. (AUKUS)) agreement and the associated Pillar II activities, promises unprecedented multinational information sharing. In this context, the Safeguarding Australia through Biotechnology Response and Engagement (SABRE) Alliance aims to foster collaborative efforts that leverage biotechnology for dual purposes, including animal pain management. This initiative uses collaborative, cross-disciplinary efforts to address complex challenges like animal pain, which are crucial for both ethical and practical dimensions of animal welfare and agricultural productivity.

## Application of success-failure to pain outcome measures: the canine brief pain inventory”

In the session “Analytic approaches to utilize outcome measures in clinical trials', Dottie Brown discussed the development of success-failure criteria for the Canine Brief Pain Inventory assessment of osteoarthritis pain in dogs.

The Canine Brief Pain Inventory (CBPI) is a publicly available owner-completed questionnaire designed to quantify the severity of chronic pain and its impact on routine activities in companion dogs. The instrument includes four questions about pain severity that are averaged to generate the Pain Severity Score (PSS) and six questions about the degree to which pain interferes with the dog's routine activities, which are averaged to generate the Pain Interference Score (PIS) (38, 39).

Rather than comparing the overall mean or median differences in scores between groups of animals, it can be important to assess whether the treatment has a measurable effect for individual animals. Particularly in the context of clinical studies for drug development, the criteria for successful treatment of an individual animal are predefined so that the success or failure of the treatment in each animal can be determined at study completion. The number of treatment successes and failures in each group (often animals that receive an active agent vs. those administered a placebo) can then be compared. This method can minimize the impact of outliers in response to treatment, particularly when sample sizes are relatively small.

The practice of pooling data from two or more independent data sets generated through identical study designs was used (40–42). The pooled data included 150 dogs from double-blind (owner and investigator/study staff), randomized, placebo-controlled clinical studies, where carprofen was used as a positive control (43). All dogs were >8 kg with a medical history, clinical signs, physical examination findings, and radiographic findings consistent with osteoarthritis. Only dogs with newly diagnosed osteoarthritis or those that had received no previous treatment for osteoarthritis were included. The CBPI was completed by the same owner for each dog at screening (Day-14 to Day -7), baseline (Day 0), and after two weeks of treatment with placebo or carprofen (Day 14).

The statistical analysis performed on this data set explored the power of defining treatment success as a reduction of 1, 2, or 3 in either or both the PSS and PIS, as well as how setting the inclusion criteria at baseline to a PSS and PIS 1, 2, or 3 affected the power of the statistical analysis to detect differences between the placebo and carprofen treatment. The treatment group summarized the number and percentage of treatment successes and failures. Possible differences between treatment groups were evaluated with the *X*^2^ test. For each definition of success within each population, power was calculated by means of a continuity-corrected 2-sided *z* test, with a = 0.05. Based on the pooled placebo & carprofen data, a study protocol to evaluate treatment effects in dogs with osteoarthritis will be most useful if:

• The inclusion criteria at baseline (Day 0) are predefined as a PSS and PIS each ≥2 and
• Success for each patient is predefined as a decrease ≥1 in PSS and a decrease ≥2 in PIS.

Although this kind of analysis requires more animals to be enrolled in each arm of a study, compared with an evaluation of median change in scores between groups, it allows for the determination of response at the individual dog level as opposed to the group level, which may be key to the pivotal evaluation of intervention efficacy.

There are several scientific approaches to applying success-failure criteria to health assessment instruments, each with its own strengths and limitations (44–46).

• Threshold-Based Criteria: Establishing specific thresholds for what constitutes a successful outcome. Limitations: Thresholds may not capture clinically meaningful changes for all patients.
• Responder Analysis: Classifying patients as responders or non-responders based on predefined criteria. Limitations: This approach may oversimplify the complexity of patient responses and ignore partial improvements.
• Minimal Clinically Important Difference (MCID): Determining the smallest change in an outcome measure that patients perceive as beneficial. Limitations: MCID can vary between populations and conditions, making it challenging to standardize.
• Composite Endpoints: Combining multiple individual outcomes into a single measure of success. Limitations: Composite endpoints can be complex to interpret and may dilute the impact of individual outcomes.

These approaches highlight the importance of selecting appropriate criteria based on the specific context and goals of the health outcome assessment.

## Validated scales for assessing acute pain in ruminants and pigs: approaches to defining success-failure and what is next?

Continuing the discussion around the interpretation of pain scales, Stelio Luna discussed the attributes of the current pain scales used in production animals.

Success in pain assessment is achieved by correctly identifying animals suffering pain (true positives) from those that do not suffer pain (true negatives). The “successful” pain scale is the one with the highest sensitivity and specificity. Other attributes that guarantee success in pain assessment are intra (repeatability) and inter-rater reliability (reproducibility).

**Figure 1:**
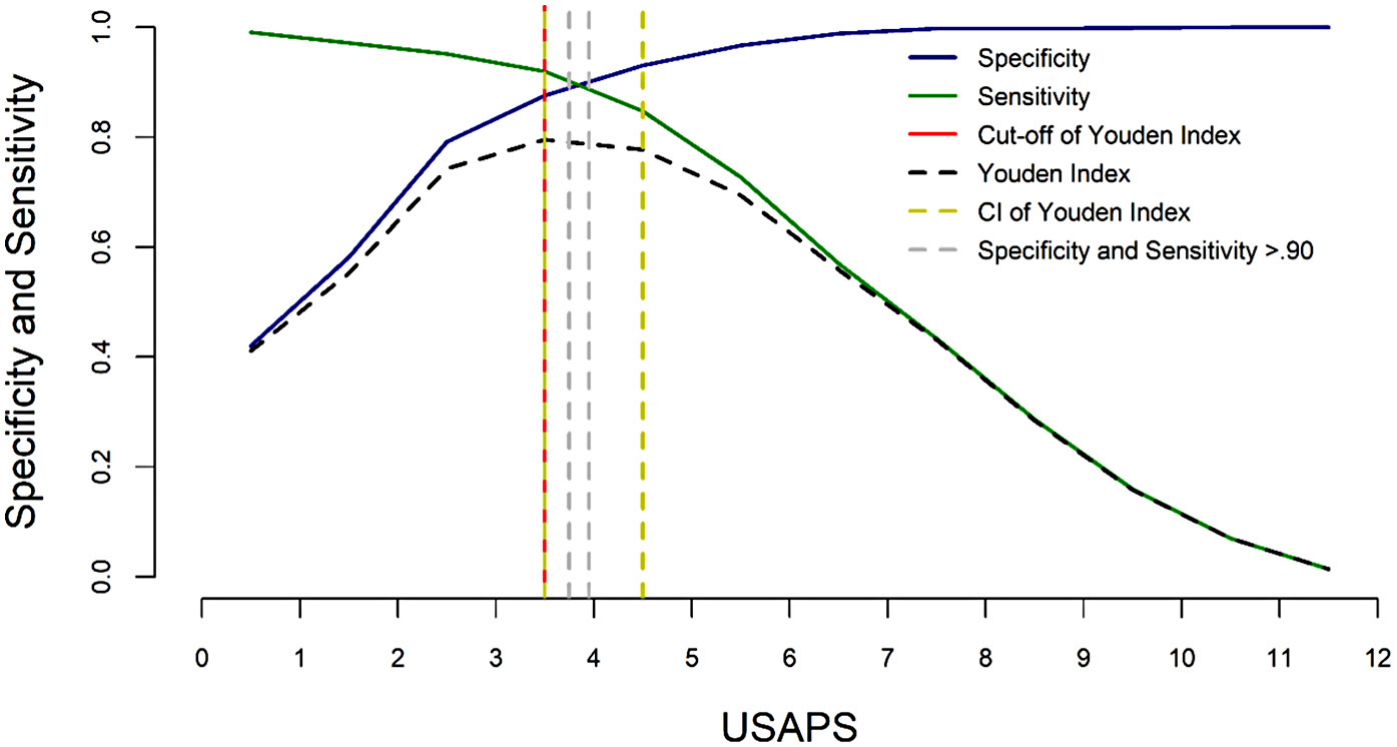
Two-graph ROC curve of the USAPS. The diagnostic uncertainty zone of the cut-off point based on the Youden index was estimated by the 95% confidence interval, calculated from 1,001 replications, and by sensitivity and specificity values >0.90. The diagnostic uncertainty zone was 4 to 5; therefore ≤3 indicates pain-free sheep (true negative), and ≥5 indicates sheep suffering pain (true positive). A score ≥4 is representative of the cut-off point for the indication of rescue analgesia. This figure was previously published in (52).

One of the best approaches to assess the methodological quality of studies and to investigate whether an instrument is validated and reliable is the COnsensus Based Standards for the Selection of Health Measurement INstruments (COSMIN) (47, 48) and GRADE (Grading of Recommendations, Assessment, Development, and Evaluations), implemented by the World Health Organization (49). According to a recent systematic review using these criteria (50) the only three behavior-based instruments that scored highly for the strength of evidence were the Unesp-Botucatu Composite Acute Pain Scales for assessing postoperative pain in cattle (UCAPS) (51), sheep (USAPS) (52), and pigs (UPAPS) (53). Since this review, the Unesp-Botucatu Goat Acute Pain Scale (UGAPS) has also been published following COSMIN guidelines (54). These scales are based only on observation and require a short time for assessment (<4 min). The scale cut-off points for indicating intervention analgesia, based on the area under the Receiver Operating Characteristic curve, increase the accuracy for decision-making on whether to treat pain, therefore minimizing oligoanalgesia and improving welfare (Figure 1). Such cut-off points can be used to define success-failure criteria.

Because validation is an ongoing process, the instruments described above still require clinical validation and further work to fill any gaps. The original pig scale was developed in weaned 38-day-old pigs, and the validity of the scale may change when used in pigs of other ages. Two recent studies clinically validated the pig scale in 5-day-old piglets (55, 56) with similar results to the weaned pigs (53) suggesting broad application to pigs of various ages for the assessment of acute pain.

The original cattle scale was validated only in Bos indicus under field conditions (51). However, recently, the UCAPS was clinically validated in Bos taurus and indicus and in the hospital environment with cows restrained in stocks (57).

The Vetpain application, created by a group of researchers headed up by Dr. Stelio Luna, is a useful tool to facilitate the use of these instruments. It is available for both IOS (https://apps.apple.com/ca/app/vetpain/id6462712970) and Android (https://play.google.com/store/apps/details?id=com.vetpain.app) and contains four steps: (1) videos that demonstrate the behaviors related to each item on the scales, for prior learning by the user, in order to improve their accuracy, repeatability, and reproducibility of the results; (2) videos for training, where the user can check their learning on the answer key before actually using the scales, (3) evaluation of pain in their animal (for owners and caregivers), or in patients under the care of Veterinarians or Technicians, or even for research where the score is automatically calculated, and (4) a defined score for each scale that indicates the decision to provide analgesia (success-failure criteria).

Ongoing research has shown that untrained students may use these instruments to detect intense pain in animals with a similar accuracy to an expert. Recent studies using artificial intelligence algorithms have distinguished the most relevant pain behaviors in sheep (58), pigs (59), and cattle (60), suggesting that future work may result in a simplification of these current scales, improving usability.

Some limitations and confounding factors in pain detection involved with the use of behavioral-based pain scales in large animals are, in acute pain studies, the residual effects of anesthesia leading to false positive results (61), the effects of time of day on behavior (54, 61), the period animals take to adapt to the hospital environment (61, 62) and, one of the most important, the observers presence which tends to underestimate pain leading to false negative results (63), suggesting that remote evaluation of pain should be preferable. Also, importantly, reliability improved when experienced observers assessed pain in horses and piglets compared to veterinary students or less experienced observers respectively (64).

## Statistical considerations when using multiple or composite endpoints

Multiple or composite endpoints may be of more relevance to the holistic assessment of the impact of pain. However, the use of multiple endpoints requires appropriate statistical approaches. Claire Ruberman discussed this topic.

Pain studies often utilize more than one outcome to evaluate a drug or intervention's effect(s). These outcomes may be evaluated separately as multiple endpoints, either as multiple primary endpoints or co-primary endpoints, or combined into a composite endpoint. Statistical considerations when choosing an appropriate endpoint or endpoints include the following aspects: (1) how to balance Type I (rejecting the null hypothesis when the null is true) and Type II errors (failing to reject the null hypothesis when the alternative is true) while controlling the Type I error; (2) what statistical methods are used to analyze different types of endpoints; (3) how potential sources of missing data such as early withdrawals are handled; and (4) how to interpret the results.

When multiple endpoints are analyzed in a single clinical trial, there is a risk of increasing the likelihood of making a false conclusion about the effectiveness of a drug if there are no appropriate adjustments for multiple endpoints and analyses (referred to as a multiplicity problem). This can be quantified as the family-wise-error rate (FWER), which is the probability of making one or more Type I errors among all hypotheses tested (65).

One method for addressing multiplicity is by defining co-primary endpoints, in which the success of the study depends on a positive outcome in all endpoints. This may be appropriate when the demonstration of treatment effect on 2 or more distinct endpoints is critical to establish clinical benefit; however, utilizing co-primary endpoints has the disadvantage of reducing power. Additionally, if multiple primary endpoints are defined such that a demonstration of a treatment effect on at least one of several primary endpoints is sufficient to establish study success, then statistical methods for controlling the FWER may be utilized, such as the Bonferroni Method or Holm or Hochberg procedures (66, 67). There are numerous different methods for controlling the FWER depending on the type of data and assumptions made about the distribution of the data; an important commonality among these methods is that they should be prespecified. A concept similar to the FWER commonly utilized in exploratory studies is the false discovery rate (FDR), which defines the expected proportion of false positive findings among all rejected hypotheses (68, 69). Similar to the FWER, there are a number of different methods for controlling the FDR, which may be appropriate when testing a large number of hypotheses (i.e., exploratory studies with a large number of endpoints of interest) when controlling the FWER may be overly conservative.

A fourth method for addressing multiplicity is defining a composite endpoint, wherein multiple distinct component endpoints are combined into a single endpoint. Composite endpoints have the advantage of avoiding choosing a primary endpoint or adjusting for multiple testing. However, they may be less beneficial in an exploratory setting if the objective is to identify which components may be impacted by the treatment. Additionally, when defining a composite endpoint, it is important to consider the clinical relevance and interpretability of both the composite endpoint and its individual components. Such considerations should include the magnitude of the response, the associated clinical benefit, and the consistency with which the clinical effect can be demonstrated, and tools such as weighting may be utilized to address the clinical importance as well as the scale and directionality of different components (70, 71). Additionally, the results of a statistical analysis of the composite endpoint apply only to the composite endpoint itself and not to its individual components, and if one is interested in conducting statistical testing on the individual components as well, then multiplicity adjustments should be considered.

## Examples of use of multiple or composite endpoints in veterinary species: food animals

Hans Coetzee highlighted two examples of using multiple and composite endpoints in veterinary studies involving food animals. The first example centered on the effectiveness studies for flunixin transdermal solution (Banamine® Transdermal), a drug approved by the FDA for the control of pain associated with foot rot in cattle. This study was conducted at two sites with 30 Holstein steers each. The study design employed a multi-faceted approach to evaluate drug efficacy. The trial design involved the experimental induction of footrot followed by a treatment evaluation phase. Multiple endpoints were used to provide a comprehensive assessment of pain control. This included traditional lameness scoring but also incorporated advanced real-time gait analysis. This technology measured specific parameters such as maximum total force and contact area on the affected limb, offering objective data to complement subjective scoring. The effectiveness criteria specified that each study site demonstrated both a statistically significant difference in the percent of animals with clinically improved lameness scores (classified as “treatment success”) and measurable and clinically relevant improvements in gait parameters in the treated group at six hours post-treatment.

The increasing prevalence of composite endpoints in veterinary behavioral studies was also discussed. This approach involves scoring various behavioral categories—such as social interactions, activity levels, posture, and feeding behaviors—on simple numerical scales. These individual scores are then aggregated to create a comprehensive behavioral score, typically out of 10 points. This method has been described in cattle and pig studies, offering a nuanced way to quantify complex behavioral patterns. These examples illustrate a growing trend in veterinary clinical research towards more holistic and multidimensional assessment methods. By employing multiple and composite endpoints, researchers can capture a more complete picture of an animal's response to treatment or environmental factors. This approach not only enhances the robustness of clinical trials but also paves the way for more targeted and effective interventions in veterinary medicine. As the field continues to advance, such sophisticated methodologies are likely to become increasingly common, further bridging the gap between veterinary and human clinical research practices. This evolution promises to yield more precise and actionable insights, ultimately benefiting animal health and welfare.

## Adaptive and other innovative pain measurement study designs

Adaptive clinical trial designs have been commonly applied to cancer studies in humans and have increasingly been considered in veterinary clinical trials. Still, they have been slow to be applied to veterinary pain studies. Qiao Zhang discussed adaptive and other innovative study designs and how they may be applied to veterinary pain research.

Adaptive designs and enrichment designs are increasingly being proposed for use in animal clinical trials to evaluate the effectiveness of an animal drug. CVM published a relevant Guidance for Industry (GFI) 268 “Adaptive and Other Innovative Designs for Effectiveness Studies of New Animal Drugs” in 2021. According to the guidance, adaptive design refers to a clinical effectiveness study design that allows for prospectively planned modifications, which may affect sample size, study duration, endpoint selection, or other design features. Enrichment design refers to the prospective use of any characteristic to select a study population in which it is more likely to detect a treatment effect than in an unselected population. The guidance provides recommendations to enhance the validity and interpretability of confirmatory studies; particularly, it points out that the designs should be prospective, with the protocol pre-specifying the type(s) of adaptation or enrichment strategy.

In a typical adaptive design, all the important study features should be pre-specified in the planning stage, including the hypothesis, number and timing of interim analyses, the statistical analysis methods, the adaptation, and the criteria for triggering the adaptation, and the specific algorithm governing adaptation decisions, etc. Possible adaptations include increasing the sample size, stopping the study early for futility, stopping the study early for convincing efficacy, or other appropriate modifications by design. Because multiple tests may be performed, the proper control of Family Wise Error Rate (FWER) is a critical consideration to be addressed in adaptive design studies, along with strategies for controlling operational bias. The following discussions are on 2 commonly used adaptive designs:

(1) Sample size re-estimation, or SSR, allows the increase of the final sample size based on interim analysis results. Conditional power is a well-established method for performing SSR. Several publications provide details on the methods to control FWER based on conditional power (72–74). While SSR can help avoid under-powering a study, we should be careful not to “over-power” a study, i.e., to choose a sample size that can power a test to detect an effect size so small that it is no longer clinically relevant. This concern may be addressed by specifying a clinically relevant treatment effect or minimal clinically important difference (MCID). Adaptation should not be conducted too early, when the results may be misguided by the highly variable interim data and unreliable estimates, or too late, when there is a limited window to adapt the study. Currently established methods of SSR may not be directly applicable to animal studies due to the differences in study design (mainly in the randomization schemes) between human and animal clinical trials, and the corresponding statistical analysis methods.
(2) Group sequential design allows for one or more prospectively planned interim analyses with the possibility to stop the trial early for convincing effectiveness. The level of significance for each test should be adjusted to properly control FWER. The timing of tests should be selected such that the interim analysis includes a sufficient sample size needed to generalize the effectiveness of the results to the target population, obtain independent substantiation of evidence, and provide reliable safety evaluation.

A placebo lead-in design is one example of an enrichment design that aims to mitigate placebo effects. There is no requirement for alpha adjustment in this design. However, a meta-analysis of 101 studies (75) of depression showed that the typical placebo lead-in design did not lower the placebo response rate, nor did it increase the drug-placebo differences. Updated versions of the placebo lead-in design have been proposed with better blinding strategies. One main concern about the enrichment design is the generalizability of the effectiveness of the results to the target population.

Adaptive and enrichment methods show benefits over a fixed sample design; however, to maintain the study's validity and integrity, any adaptation or enrichment should be pre-specified at the design stage. Appropriate statistical methods should be applied to account for the adaptations, including the control of the FWER. When interpreting and generalizing the results to the target population, the design should be carefully considered.

## Future directions

As veterinary and comparative medicine embraces the “biopsychosocial” model of pain we are going to see expanded opportunities to develop measurement approaches in multiple new areas across species, such as cognitive, affective and social aspects. The social effects of, and influences on, pain have been explored in rodent studies (76, 77). Other species offer exciting translational opportunities—for example, dogs have relevant emotions, cognitive responses, and established social relationships with humans that frequently mirror those between human family members. Indeed, even compared to non-human primates, dogs' performance on many cognitive tasks is more human-like than our closest primate relatives (78, 79).

Artificial Intelligence, in its many forms, offers exciting opportunities to improve the measurement of pain across species. Simplistically, near future advances will fall into two broad buckets—(1) automating processes that can already be performed—such as automation of facial grimace detection in mice (80). (2) Development of novel approaches to the measurement of the impact of pain, for example through assessment of high frequency inertial movement unit data in animals with chronic musculoskeletal pain. Thus we will see advances in efficiency as well as novel approaches to measure pain. In some respects, the opportunities for the application of AI to the varied impacts of pain may be greater in non-human species where the lack of verbal self-report has forced consideration of other measures, and because AI is already embraced in many sectors, such as farming. However, just as in human medicine, determining “ground truth” is absolutely critical to the development of algorithms (81).

With advances in the measurement of pain, and our ability to classify pain states across species, coupled with improved annotation of genomes across species and the accessibility of “omics” technology, we are likely to see the development of useful biomarkers of pain states.

## Summary

The ability to measure the impact of pain in animals is fundamental to any advances in the development of novel pain therapeutic approaches. The 2023 PAW meeting brought together researchers and clinicians from academia, government, practice, and industry, all of whom had interest and expertise in human and/or animal pain assessment, to discuss the current and future status of pain assessment. Such multidisciplinary forums for discussion are critical in order to bridge the “silos” we all work in and to enrich the power of translational research. The 2023 meeting provided updates and insights into the current status of the measurement of pain. Additionally, it facilitated discussions on expanded opportunities to develop novel and clinically meaningful outcome measures. A key concept presented was the biopsychosocial model of pain which considers the pain experience across the biological, psychological and social domains. This model provides both opportunities for novel measurement methods as well as novel interventions. In recognition of the increasing application of artificial or augmented intelligence (AI) and machine learning to all aspects of life, including in biomedical research, the discussion of the application of AI to facilitate the measurement of pain was timely and forward thinking. The 2025 PAW meeting (https://www.PAW-2025.com) will continue to explore the theme of application of AI to pain measurement.

Collaborative discussions, such as those fostered by the PAW meeting are critical to the advancement of comparative and translational pain assessment and management, and will help shape future approaches to translational preclinical data analysis with a focus on meaningful changes that have relevance to human drug development.
