# Supplementary material for: Advancing outcome measure development and analytical approaches: Pain in Animals Workshop 2023
Source: Front Pain Res (Lausanne). 2025 Aug 21;6:1615862. doi: 10.3389/fpain.2025.1615862 (PMC12408590; doi:10.3389/fpain.2025.1615862)
Supplement: Supplementary file 1 [file Datasheet1.pdf]

PAIN IN ANIMALS WORKSHOP 2023 (PAW2023)

Advancing Outcome Measure Development and Analytical Approaches

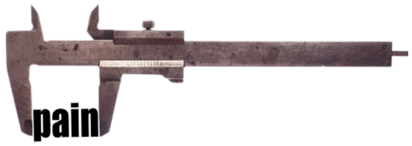

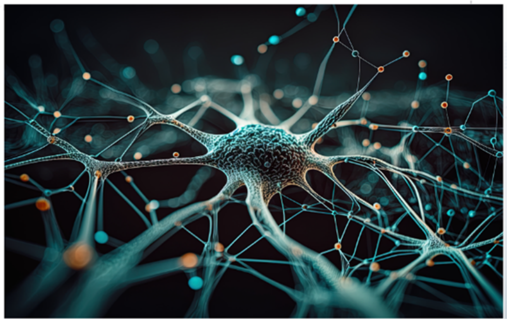

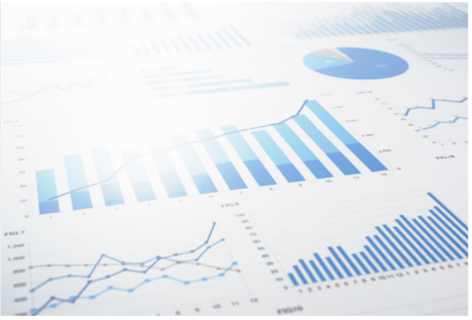

September 26 - 27, 2023 • National Institutes of Health • Bethesda, Maryland

Thank you to our sponsors

The MAYDAY Fund

NIH National Institute of Neurological Disorders and Stroke

MORRIS ANIMAL FOUNDATION

Comparative Pain Research and Education Centre

Tuesday, September 26, 2023

TIME	DESCRIPTION	SPEAKER
8:00am-9:00am	Registration /Check-In	
9:00am-9:10am	Welcome	
	**Updates on Validated Approaches to Measuring Pain**	
9:10am-9:50am	Michele Sharkey PAW Lecture: Outcome Assessment in Veterinary Pain Studies: The Yellow Brick Road Continues	Dottie Brown, MSCE, DVM, DACVS<br>Mars Veterinary Health
9:50am-10:10am	Measurement Properties of Pain Scoring Instruments in Farm Animals	Marina Cayetano Evangelista, MV, MSc, PhD<br>Université de Montréal
10:10am-10:30am	Biomarker Update: What Progress Have We Made?	Daniel Barratt, BSc(Hons),PhD<br>University of Adelaide
10:30am-10:50am	BREAK	
10:50am-11:10am	Artificial Intelligence in Veterinary Medicine	Parminder S. Basran, PhD, FCCPM<br>Cornell University
11:10am-11:30pm	Application of AIML to Large Animals for Pain Measurement	Eduarda Bortoluzzi, BVMS, MS, PhD<br>Kansas State University
11:30am-12:00pm	*Discussion on advances in measurement tools, use of biomarkers and potential application of AI to pain measurement*	**Panel Discussion**<br>(Evangelista, Barratt, Basran, Bortoluzzi)
12:00pm-1:40pm	**LUNCH**<br>12:15 – 12.45 American Association of Swine Veterinarians "Assessing pain in pigs: A collaborative effort by industry, academia, and government to advance pig welfare."<br><br>12:45 – 1.15 Recent Advances in Rodent Pain	Sherrie Webb, MSc<br>American Association of Swine Veterinarians<br><br>Liezl Maree, MS<br>Salk Institute for Biological Studies

	Measurement: Automated Detection of Behaviors and Application of Deep Learning	
	**Opportunity Areas (Biopsychosocial) for Additional Outcome Measure Development**	
1:40pm-2:00pm	Overview of the Domains Impacted by Pain	B. Duncan Lascelles, BSc, BVSc, PhD, FRCVS<br>NC State University
2:00pm-2:50pm	The Biospychosocial Model of Pain	Mark Hutchinson, BSc, PhD<br>University of Adelaide
2:50pm-3:20pm	BREAK	
3:20pm-3:50pm	Domains Impacted by Acute Pain	Ishmail Abdus-Saboor, PhD<br>Columbia University
3:50pm-4:20pm	Domains Impacted by Chronic Pain	Kathleen Sluka, PT, PhD, FAPTA<br>University of Iowa
4:20pm-4:40pm	*Discussion: Are Different Pain Conditions Impacting Various Domains Differently?*	**Panel Discussion**<br>(Lascelles, Hutchinson, Abdus-Saboor, Sluka)
4:45pm-7:00pm	Social w/ Drinks & Posters	

Wednesday, September 27, 2023

TIME	DESCRIPTION	SPEAKER
	**Analytic Approaches to Utilize Outcome Measures in Clinical Trials**	
9:00am-9:20am	Approaches to Defining Clinically Meaningful Change in Trial Outcome Measures	Jennifer Gewandter, PhD, MPH<br>University of Rochester

9:20am-9:40am	Creating MCID Thresholds in Veterinary Medicine: Estimating Minimal Clinically-Important Difference (MCID) for LOAD and COI	John F. Innes, BVSc, PhD, CertVR DSAS(orth),FRCVS<br>University of Liverpool
9:40am-9:50am	Discussion of Application of Success-Failure to Pain Outcome Measures: CBPI	Dottie Brown, MSCE, DVM, DACVS<br>Mars Veterinary Health
9:50am-10:10am	BREAK	
10:10am-10:20am	Validated Scales for Assessing Acute Pain in Ruminants and Pigs: Approaches to Defining Success-Failure and What is Next?	Stelio Luna, DVM, Ms, PhD, DipECVAA, CVA (IVAS)<br>São Paulo State University
10:20am-10:30am	Success-Failure: Ground Reaction Forces	Michael Conzemius, DVM, PhD, DACVS<br>Gilbert Queen Creek
10:30am-10:50am	Use of AUC in Human Pain Studies	James N. Campbell, MD<br>Centrexion Therapeutics
10:50am-11:20am	*Discussion: Analytic Approaches to Utilize Outcome Measures in Clinical Trials*	**Panel Discussion**<br><br>(Gewandter, Innes, Luna, Conzemius, Campbell)
11:20am-12:30pm	**LUNCH**	
12:30pm-12:50pm	Primary Endpoints: Single, Multiple Endpoints, or Composite Endpoints?	John T. Farrar, MD, PhD<br>University of Pennsylvania
12:50pm-1:10pm	Statistical Considerations When Using Multiple or Composite Endpoints	Claire Ruberman, PhD<br>FDA CVM
1:10pm-1:30pm	Examples of Use of Multiple or Composite Endpoints in Veterinary Species: Food Animals	Hans Coetzee, BVSc, Cert CHP, PhD, DACVCP, DACAW, DipECAWBM<br>Kansas State University

1:30pm-1:50pm	Adaptive and Other Innovative Pain Measurement Study Designs	Qiao Zhang, MS, PhD<br>FDA CVM
1:50pm-2:10pm	*Discussion on Pros and Cons of Multiple / Composite Endpoints, Design Adaptations in Veterinary Clinical Pain Research*	**Panel Discussion**<br><br>(Farrar, Ruberman, Coetzee, Zhang)
2:10pm-2:30pm	*Open Discussion and Future Planning*	
