# Supplementary material for: Advancing outcome measure development and analytical approaches: Pain in Animals Workshop 2023
Source: Front Pain Res (Lausanne). 2025 Aug 21;6:1615862. doi: 10.3389/fpain.2025.1615862 (PMC12408590; doi:10.3389/fpain.2025.1615862)
Supplement: Supplementary file 2 [file Datasheet2.pdf]

PAIN IN ANIMALS WORKSHOP 2023 (PAW2023)

Advancing Outcome Measure Development and Analytical Approaches

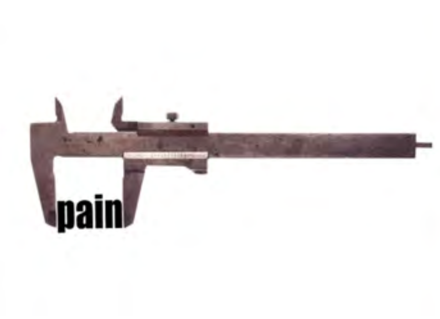

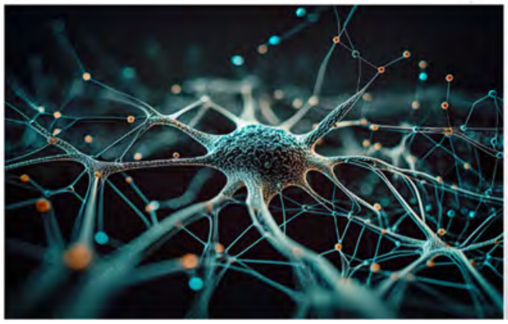
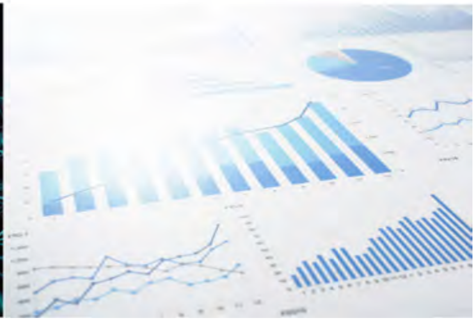

September 26 - 27, 2023 • National Institutes of Health • Bethesda, Maryland

Thank you to our sponsors

The MAYDAY Fund

National Institute of Neurological Disorders and Stroke

MORRIS ANIMAL FOUNDATION

Comparative Pain Research and Education Centre

Tuesday, September 26, 2023

TIME	DESCRIPTION	SPEAKER
8:00am-9:00am	Registration /Check-In	
9:00am-9:10am	Welcome	
	**Updates on Validated Approaches to Measuring Pain**	
9:10am-9:50am	Michele Sharkey PAW Lecture: Outcome Assessment in Veterinary Pain Studies: The Yellow Brick Road Continues	Dottie Brown, MSCE, DVM, DACVS<br>Mars Veterinary Health
9:50am-10:10am	Measurement Properties of Pain Scoring Instruments in Farm Animals	Marina Cayetano Evangelista, MV, MSc, PhD<br>Université de Montréal
10:10am-10:30am	Biomarker Update: What Progress Have We Made?	Daniel Barratt, BSc(Hons),PhD<br>University of Adelaide
10:30am-10:50am	BREAK	
10:50am-11:10am	Artificial Intelligence in Veterinary Medicine	Parminder S. Basran, PhD, FCCPM<br>Cornell University
11:10am-11:30pm	Application of AIML to Large Animals for Pain Measurement	Eduarda Bortoluzzi, BVMS, MS, PhD<br>Kansas State University
11:30am-12:00pm	*Discussion on advances in measurement tools, use of biomarkers and potential application of AI to pain measurement*	**Panel Discussion**<br>(Evangelista, Barratt, Basran, Bortoluzzi)
12:00pm-1:40pm	**LUNCH**<br>12:15 – 12.45 American Association of Swine Veterinarians "Assessing pain in pigs: A collaborative effort by industry, academia, and government to advance pig welfare."<br><br>12:45 – 1.15 Recent Advances in Rodent Pain	Sherrie Webb, MSc<br>American Association of Swine Veterinarians<br><br>Liezl Maree, MS<br>Salk Institute for Biological Studies

	Measurement: Automated Detection of Behaviors and Application of Deep Learning	
	**Opportunity Areas (Biopsychosocial) for Additional Outcome Measure Development**	
1:40pm-2:00pm	Overview of the Domains Impacted by Pain	B. Duncan Lascelles, BSc, BVSc, PhD, FRCVS<br>NC State University
2:00pm-2:50pm	The Biospychosocial Model of Pain	Mark Hutchinson, BSc, PhD<br>University of Adelaide
2:50pm-3:20pm	BREAK	
3:20pm-3:50pm	Domains Impacted by Acute Pain	Ishmail Abdus-Saboor, PhD<br>Columbia University
3:50pm-4:20pm	Domains Impacted by Chronic Pain	Kathleen Sluka, PT, PhD, FAPTA<br>University of Iowa
4:20pm-4:40pm	*Discussion: Are Different Pain Conditions Impacting Various Domains Differently?*	**Panel Discussion**<br>(Lascelles, Hutchinson, Abdus-Saboor, Sluka)
4:45pm-7:00pm	Social w/ Drinks & Posters	

Wednesday, September 27, 2023

TIME	DESCRIPTION	SPEAKER
	**Analytic Approaches to Utilize Outcome Measures in Clinical Trials**	
9:00am-9:20am	Approaches to Defining Clinically Meaningful Change in Trial Outcome Measures	Jennifer Gewandter, PhD, MPH<br>University of Rochester

9:20am-9:40am	Creating MCID Thresholds in Veterinary Medicine: Estimating Minimal Clinically-Important Difference (MCID) for LOAD and COI	John F. Innes, BVSc, PhD, CertVR DSAS(orth),FRCVS<br>University of Liverpool
9:40am-9:50am	Discussion of Application of Success-Failure to Pain Outcome Measures: CBPI	Dottie Brown, MSCE, DVM, DACVS<br>Mars Veterinary Health
9:50am-10:10am	BREAK	
10:10am-10:20am	Validated Scales for Assessing Acute Pain in Ruminants and Pigs: Approaches to Defining Success-Failure and What is Next?	Stelio Luna, DVM, Ms, PhD, DipECVAA, CVA (IVAS)<br>São Paulo State University
10:20am-10:30am	Success-Failure: Ground Reaction Forces	Michael Conzemius, DVM, PhD, DACVS<br>Gilbert Queen Creek
10:30am-10:50am	Use of AUC in Human Pain Studies	James N. Campbell, MD<br>Centrexion Therapeutics
10:50am-11:20am	*Discussion: Analytic Approaches to Utilize Outcome Measures in Clinical Trials*	**Panel Discussion**<br><br>(Gewandter, Innes, Luna, Conzemius, Campbell)
11:20am-12:30pm	**LUNCH**	
12:30pm-12:50pm	Primary Endpoints: Single, Multiple Endpoints, or Composite Endpoints?	John T. Farrar, MD, PhD<br>University of Pennsylvania
12:50pm-1:10pm	Statistical Considerations When Using Multiple or Composite Endpoints	Claire Ruberman, PhD<br>FDA CVM
1:10pm-1:30pm	Examples of Use of Multiple or Composite Endpoints in Veterinary Species: Food Animals	Hans Coetzee, BVSc, Cert CHP, PhD, DACVCP, DACAW, DipECAWBM<br>Kansas State University

1:30pm-1:50pm	Adaptive and Other Innovative Pain Measurement Study Designs	Qiao Zhang, MS, PhD<br>FDA CVM
1:50pm-2:10pm	*Discussion on Pros and Cons of Multiple / Composite Endpoints, Design Adaptations in Veterinary Clinical Pain Research*	**Panel Discussion**<br><br>(Farrar, Ruberman, Coetzee, Zhang)
2:10pm-2:30pm	*Open Discussion and Future Planning*	

Pain in Animals 2023 Speakers

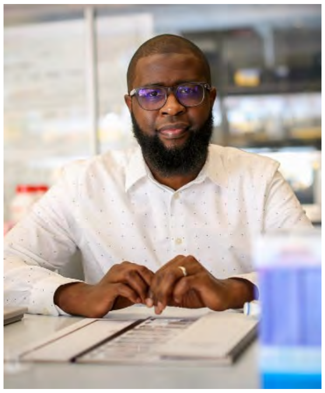

Ishmail Abdus-Saboor, PhD

Associate Professor & Principal Investigator, Columbia University's Zuckerman Mind Brain Behavior Institute

Ishmail Abdus-Saboor is an associate professor of biological sciences at Columbia University's Zuckerman Mind Brain Behavior Institute. Ishmail attended an HBCU for college, North Carolina A&T University, followed by a Ph.D. in Cell and Molecular Biology at the University of Pennsylvania. Following training in developmental genetics, he moved into sensory neuroscience as a postdoc at Cornell and the University of Pennsylvania. He has received many competitive honors and awards including an NIH K99/R00 Pathway to Independence Award and Burroughs Wellcome Fund PDEP Fellowship as a postdoc. Since opening his lab in 2018, he has been named a Rita Allen Foundation Scholar, Alfred P. Sloan Research Fellow, Pew Biomedical Scholar, Kavli Fellow of the National Academy of Sciences, HHMI Freeman Hrabowski Scholar, McKnight Scholar, and a recipient of the Chan Zuckerberg Initiative Science Diversity Leadership Award and NIH DP2 New Innovator Award.

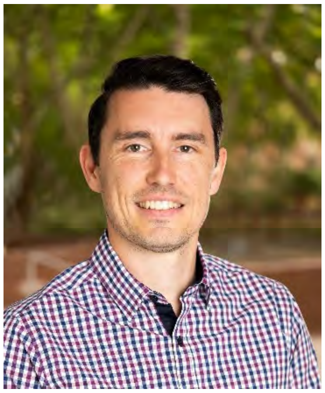

Daniel Barratt, BSc, PhD

School of Biomedicine, University of Adelaide

Dr Barratt is a post-doctoral researcher in the Neuroimmunopharmacology and Clinical Pharmacogenomics and Drug Disposition Laboratories of the University of Adelaide, Australia. He has nearly 20 years' research experience in the field of precision medicine; examining mechanisms and biomarkers of drug response through the integration of multi-omics, drug disposition, demographic and disease data. His current research focuses on the discovery and development of blood-based biomarkers of pain and analgesic response in humans to support precision pain medicine, and in livestock to support objective welfare assessment and development and approval of novel analgesics.

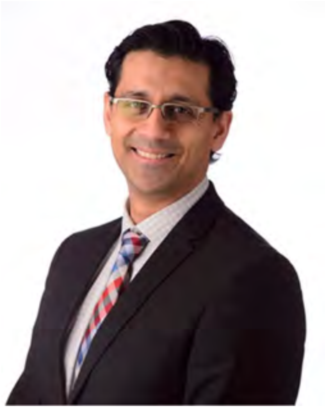

Parminder S. Basran, PhD, FCCPM

Department of Clinical Sciences. Cornell University – College of Veterinary Medicine

Parminder S. Basran PhD (2002- University of Calgary), MSc (1997-University of Alberta) is a Associate Research Professor at Cornell University, College of Veterinary Medicine. He is a Member (2004) and Fellow (2010) of the Canadian College of Physicists in Medicine. The Basran Lab, named the Veterinary Artificial Intelligence in Diagnostic Imaging and Radiotherapy (VAIDER Lab), has 3 main areas of focus in Veterinary Medicine: Radiation Dosimetry and Treatment Planning; Medical Image Processing and Analysis; and Medical Physics Training and Education. Medical imaging processing projects focus on the use of machine learning in veterinary medicine, including multi-omics (Ultrasound + CBC and blood serum) approaches for discriminating lymphoma from inflammatory bowel disease in cats, leveraging machine-learning from x-ray images of the horse fetlock to predict the risk of injury in Thoroughbred racehorses, and adopting computer vision technologies to detect and control mastitis in dairy cows. Radiation dosimetry and treatment planning projects focus on high-precision radiation medicine to animals including limb preservation for dogs diagnosed with appendicular osteosarcoma, the development of standardized radiation dosimetry techniques for accreditation and benchmark performance in veterinary radiation oncology, and surveying the technical and human resources in the delivery of safe and effective radiation therapy in the veterinary setting. Projects related to education include the use of gaming technologies such as Lego© and 3D printing in medical physics education, and developing open-access educational content for medical physics education in low-to-middle-income countries.

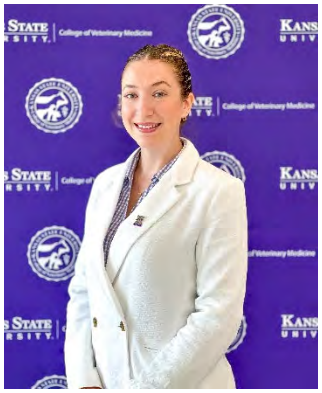

Eduarda M. Bortoluzzi, MV, MS, PhD

Animal Welfare, Department of Anatomy and Physiology, College of Veterinary Medicine, Kansas State University,

Dr. Eduarda Bortoluzzi is an Assistant Professor of Animal Welfare at the Department of Anatomy and Physiology at Kansas State University. She holds a veterinary medicine degree from the Federal University of Pampa, Brazil. She completed her master’s and Ph.D. degrees at the Department of Animal Sciences and Industry at Kansas State University. Her interests include animal behavior and the welfare of domestic species, emphasizing the development of new welfare metrics using artificial intelligence and machine learning.

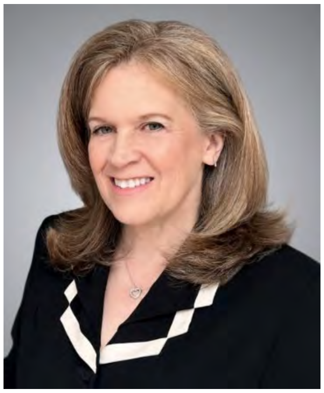

Dorothy (Dottie) Cimino Brown, MS, DVM, DACVS

Science and Healthcare Innovation for Mars Veterinary Health

Dr Dottie Cimino Brown is the Vice President of Science and Healthcare Innovation for Mars Veterinary Health (MVH) where she provides strategic and innovative leadership to build the science function across MVH globally. She joined Mars in May of 2023 from Elanco Animal Health where she led the Translational Comparative Medical Research program and focused on therapeutic drug development, directing teams in Research & Early Development to deliver innovative approaches to disease prevention and treatment. Prior to joining Elanco in 2017, had 25 years at the University of Pennsylvania as a Professor of Surgery and Clinical Epidemiologist, where she led a translational research program that focused on the measurement and management of chronic pain in companion animals and directed the Veterinary Clinical Investigation Centre. More than 130 scientific publications and book chapters mark her 25 years in academia.

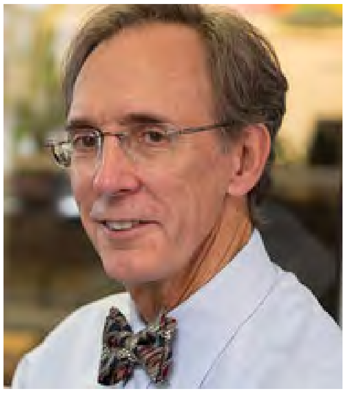

James N. Campbell, M.D

President and Chief Scientific Officer, Centrexion Therapeutics
Professor Emeritus of Neurosurgery, Johns Hopkins University

Dr. Campbell serves as President, co-founder, and Chief Scientific Officer of Centrexion Therapeutics, a Boston based company involved in the development of new therapeutics to treat chronic pain. He is Professor Emeritus of Neurosurgery at Johns Hopkins University. In the past, he has served as Vice Chairman and Director of the Residency Training in the Department of Neurosurgery at Hopkins, and presently serves as head of the Blaustein Endowment for the Pain Research and Education, based at Johns Hopkins Hospital. He has served previously as Executive-in-Residence at the California based venture firm, InterWest Partners, and for several years also served as a consultant. Jim has co-founded several companies in biotechnology in the areas of immuno-therapeutics, chronic pain, and other neurological diseases. Dr. Campbell has a long record of accomplishment in academic medicine and neuroscience research. For much of his clinical career at Hopkins he was listed in Best Doctors in America. His work has been cited more than 15,000 times. He has edited textbooks, published well over 100 peer-reviewed research papers, and served on the editorial board of leading academic journals. He has received several national awards for research and public service, including the Jacob Javits Neuroscience Investigator Award from the National Institutes of Health; the Grass Award from the Society of Neurological Surgery; the Kerr Award for Research from the American Pain Society; and a Pain Research Award from Bristol Myers Squibb. He has also received awards for Public Service and Leadership from the American Pain Society and the American Pain Foundation. Dr. Campbell received his B.A. from the University of Michigan with High Honors and High Distinction and his M.D. degree from Yale University. He completed his residency in Neurosurgery at Johns Hopkins Hospital and obtained post-doctoral training in Neurophysiology in the Department of Physiology at Johns Hopkins. Dr. Campbell is a Diplomate of the American Board of Neurological Surgery

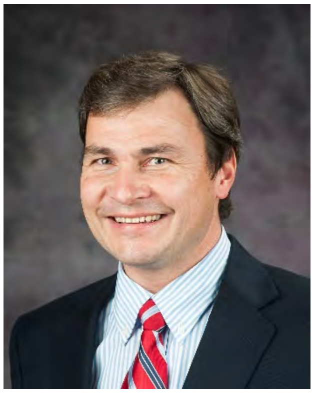

Johann (Hans) Coetzee BVSc, Cert CHP, PhD, DACVCP, DACAW, DipECAWBM (AWSEL), MRCVS
Department of Anatomy and Physiology, Kansas State University

Dr. Hans Coetzee is a University Distinguished Professor, and Head of the Department of Anatomy and Physiology at Kansas State University. He earned his Bachelor of Veterinary Science degree from the University of Pretoria, South Africa in 1996. After graduation he worked for four years in mixed animal practice in Northern Ireland followed by 2 years in pharmaceutical research and development at Norbrook Laboratories Ltd. He was awarded a specialist Certificate in Cattle Health and Production from the Royal College of Veterinary Surgeons (London) in 2000 and earned a doctorate in Veterinary Microbiology from Iowa State University in 2005. He holds dual board certification in the American College of Veterinary Clinical Pharmacology and American College of Animal Welfare and is a European Specialist in Animal Welfare Science, Ethics and Law. His professional interests include developing pain assessment tools and identifying practical methods to provide pain relief in livestock. He has published over 200 peer-reviewed scientific papers and received over $20 million in research funding. In his free time, he enjoys fly fishing and spending time with his wife and his twin daughters.

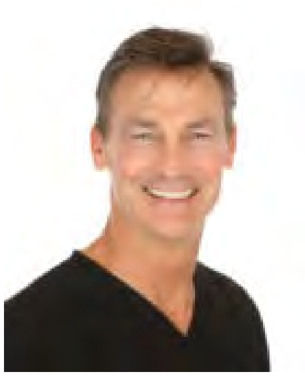

Michael G. Conzemius, DVM, PhD, DACVS

Gilbert Queen Creek Vets

Dr. Mike Conzemius received his DVM and PhD in Biomedical Engineering from Iowa State University. He completed his surgical residency at the University of Pennsylvania. He has served as a faculty member at the University of Pennsylvania, Iowa State University at the University of Minnesota. He is a Diplomate of the American College of Veterinary Surgeons and an ACVS Founding Fellow in Joint Replacement Surgery.

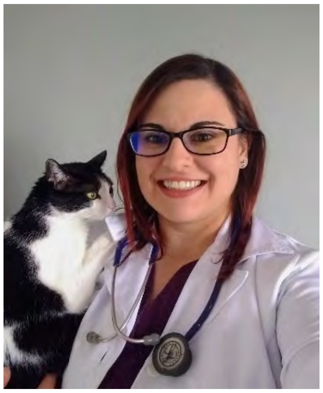

Marina Cayetano Evangelista, MV, MSc, PhD

Senior Research Anesthetist, Charles River Laboratories

Veterinary doctor (MV) graduated from the University of Sao Paulo (USP), Brazil. Completed a residency in veterinary anesthesiology at the Sao Paulo State University (UNESP - Botucatu), Brazil. Obtained a Master's degree at the University of Sao Paulo with an international collaboration program (research externship in Canada). Recently obtained her Ph.D. from the Université de Montréal, Canada, and currently works as a Senior Research Anesthetist at Charles River Laboratories - Senneville (Canada).

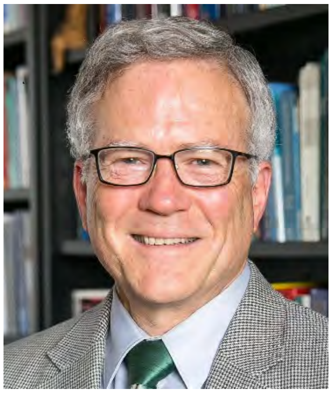

John T. Farrar, MD, MSCE, PhD

Perelman School of Medicine, University of Pennsylvania

Dr Farrar is a Professor of Epidemiology, Neurology, and Anesthesia at the Perelman School of Medicine (PSOM) at the University of Pennsylvania and President of the US Association for the Study of Pain. He has been involved in clinical research for over 30 years, involving randomized trials (RCTs), cohort studies, and methodologic studies of pain and associated symptoms. His focus has been on the measurement and meaning of pain and related symptoms, study design, study conduct, analysis, and interpretation of studies of the efficacy of pain therapeutics. He is currently the Principal Investigator (PI) of an FDA-funded contract to study the use of opioids in treating pain and a second contract to evaluate treatments for acute pain. He is the PI of the Penn Specialized Clinical Center as part of the NIH-HEAL EPPIC-Network initiative. He is also a co-investigator for the NIDDK-funded data coordinating center (DCC) for the "Multidisciplinary Approach to Pelvic Pain (MAPP) Research Network". Additionally, he teaches courses in health measurement, clinical trials, and grant writing, and co-directs the Master of Science in Clinical Epidemiology (MSCE) program, a two-year program focused on launching the independently funded clinical research careers of 25-30 fellows per year.

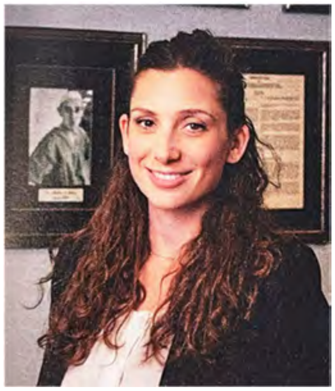

Jennifer Gewandter, PhD, MPH

Department of Anesthesiology and Pain Medicine / Department of Dentistry / Department of Neurosurgery, University of Rochester School of Medicine & Dentistry

Dr Gewandter is an Associate Professor at the University of Rochester and a former Associate Director of the FDA Public-Private partnership, ACTTION. Her research and scholarly activities are focused on optimizing the design, execution, and transparent dissemination of clinical trials for pain and peripheral neuropathy treatments as well as researching interventions for chemotherapy-induced peripheral neuropathy. She has co-authored over 85 peer-reviewed publications and is an Associate editor for the Clinical Journal of Pain. She has mentored over 25 medical students, residents, fellows, and clinical faculty in research design and scientific writing.

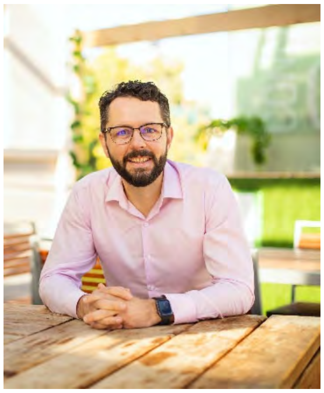

Mark Hutchinson, PhD

School of Biomedicine at the University of Adelaide
ARC Centre of Excellence for Nanoscale BioPhotonics

Professor Hutchinson is the Director of the ARC Centre of Excellence for Nanoscale BioPhotonics (CNBP), an Australian Research Council Future Fellow, head of the Neuroimmunopharmacology Laboratory, and a Professor within the School of Biomedicine at the University of Adelaide. Mark is also President of Science and Technology Australia, the peak body in Australia that represents 115,000 scientists. This is the Aussie equivalent to the AAAS. He is a Ministerial appointment to the ARC CEO advisory council, co-Chair of the Safeguarding Australia through Biotechnology Response and Engagement (SABRE) Alliance, the Chair of the Australian Pain Solutions Research Alliance, and a founding member of The Animal Welfare Collaborative.

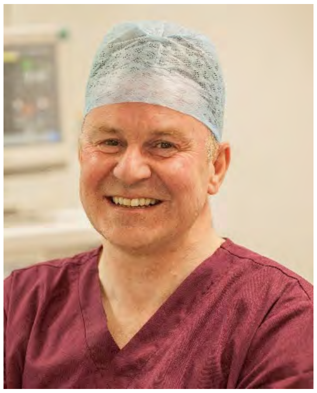

John Innes BVSc, PhD, DSAS(orth) FRCVS

Director, Movement Referrals, Cheshire, UK
Honorary Professor, University of Liverpool

John Innes graduated as a veterinarian in 1991 from University of Liverpool and he spent 22 years in academia (Bristol 1991-2001; Liverpool 2001-2013). After completing a PhD in the Rheumatology Unit at Bristol Medical School, he developed a research group investigating veterinary and comparative orthopaedics, particularly joint diseases. In 2013, he was recruited as Referrals Director, and then latterly Chief Veterinary Officer, of a major corporate veterinary group in UK. He decided to return to a veterinary-led environment in 2022 and he co-founded 'Movement Referrals: Independent Veterinary Specialists' with like-minded veterinary specialists. John was elected chair of the RCVS Fellowship Board 2019-2022 and received the BSAVA Blaine Award in 2023 for contributions to veterinary science.

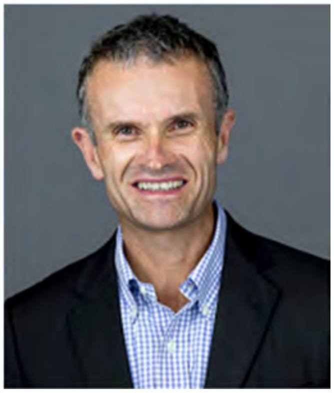

B. Duncan X. Lascelles, BSc, BVSC, PhD, FRCVS, CertVA, DSAS(ST), DECVS, DACVS

Professor of Translational Pain Research and Surgery
Director, Translational Research in Pain [TRiP] Program
Director, Comparative Pain Research and Education Center
North Carolina State University College of Veterinary Medicine

Dr. Lascelles is Professor in Small Animal Surgery and Professor of Comparative and Translational Pain Research at North Carolina State University. His research program (Translational Research in Pain [TRiP]) develops methods to measure pain associated with spontaneous disease in animals, and seeks to understand the underlying neurobiology. His work improves pain control in companion animals, and facilitates analgesic development in human medicine through proof of concept studies in companion animals, and discovery of relevant mechanisms involved in specific, naturally occurring painful disease states. He has a particular interest the measurement of movement in relation to pain, and in the role of neurotrophins and neurotrophic factors, and downstream signaling, in pain states. He is director of the Comparative Pain Research and Education Centre (CPREC). He has authored over 230 peer reviewed research papers and reviews, ~400 research abstracts, and ~ 30 book chapters.

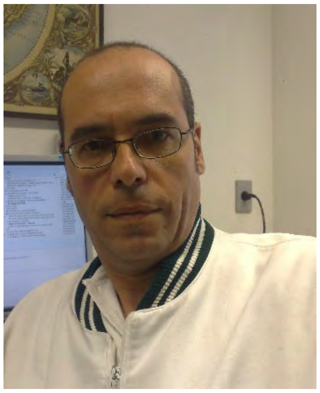

Stelio Pacca Loureiro Luna, DVM, Msci, PhD, Diplomate ECVAA, IVAS cert

School of Veterinary Medicine and Animal Science –University of São Paulo State

Stelio received his graduation in Veterinary Medicine at the School of Veterinary Medicine and Animal Science (FMVZ)–University of São Paulo State (UNESP) in 1984. After graduation, he performed Residence in Veterinary Anesthesiology and finished his MSc at the same institution in 1990. He performed his Ph.D. at the University of Cambridge, UK (1990-1993), is Certified by the International Veterinary Acupuncture Society (IVAS) since 2002, and has been a Diplomate by the European College of Veterinary Anaesthesiology and Analgesia since 1995, becoming a non-practice Diplomate in 2022. Until 2022 he was a Full Professor of Veterinary Anesthesiology at the Veterinary Medicine of the School of Veterinary Medicine and Animal Science (FMVZ)–University of São Paulo State (UNESP), where he had been working as a lecturer since 1987. Since his retirement, he has been a volunteer at the same School, where he performs research and teaches undergraduate and graduate students. His main area of interest is pain assessment and management, animal welfare,
veterinary anesthesia, and acupuncture.

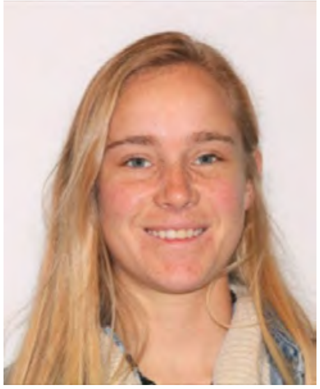

Liezl Maree, BS, MS

Salk Institute for Biological Sciences

Liezl is a scientific programmer (software engineer) in the Talmo Lab at the Salk Institute for Biological Sciences. She received her BS in Electrical and Electronics Engineering and MS in Intelligent Systems, Robotics and Control from UCSD and previously worked as an engineer at General Atomics and more. She is working on the core software stack for computer vision and deep learning-based applications, including SLEAP.

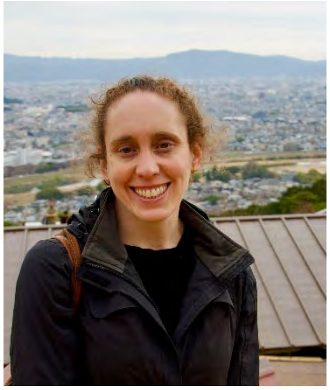

Claire Ruberman, PhD

Office of New Animal Drug Evaluation, Center for Veterinary Medicine,
Food and Drug Administration

Claire Ruberman is a Mathematical Statistician in the Office of New Animal Drug Evaluation in the Center for Veterinary Medicine at the Food and Drug Administration. She received a PhD in Biostatistics from the Johns Hopkins Bloomberg School of Public Health and a BA in Mathematics from Pomona College.

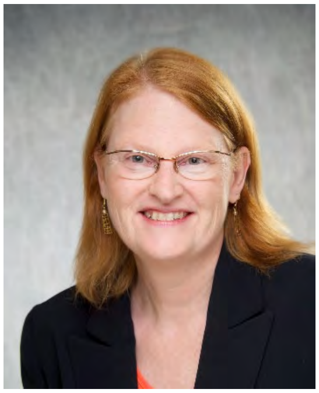

Kathleen A. Sluka, PT, PhD, FAPTA

Department of Physical Therapy and Rehabilitation Science
The University of Iowa Carver College of Medicine,

Dr. Sluka is a professor in the Department of Physical Therapy and Rehabilitation Science at the University of Iowa. Dr. Sluka's translational research program focuses on the neurobiology of musculoskeletal pain as well as the mechanisms and effectiveness of non-pharmacological pain treatments. She has published over 250 peer-reviewed manuscripts, numerous book chapters, and a textbook on Pain Mechanisms and Management for the Physical Therapist. She is actively involved in the International Association for the Study of Pain, and the American Physical Therapy Association serving on committees, task forces and society boards.

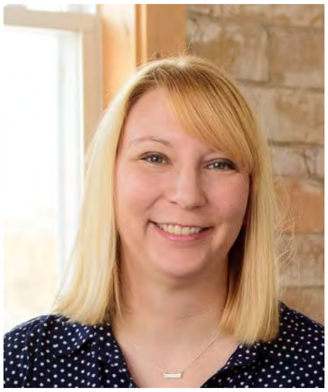

Sherrie Webb, M.S.

Associate Editor, Journal of Swine Health and Production (JSHAP)

Sherrie Webb is the Associate Editor of the Journal of Swine Health and Production, the peer-reviewed scientific journal of the American Association of Swine Veterinarians. In this role, Sherrie is responsible for editing scientific articles submitted for publication to ensure manuscripts conform to the journal's standards. She also held the role of AASV's Director of Animal Welfare from 2018-2022, and continues to oversee select projects related to animal welfare for the organization. Prior to joining AASV, Webb was Director of Animal Welfare for the National Pork Board for 13 years where she used her expertise in swine welfare to advise on animal welfare issues and aid in the development of welfare outreach and education opportunities. Webb received her master's degree in animal science with a focus on stress physiology in swine from the University of Illinois in 2006. Sherrie and her family reside in Iowa.

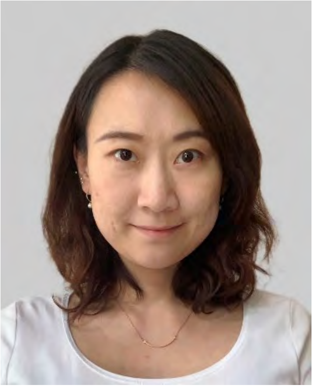

Qiao Zhang, PhD

Office of New Animal Drug Evaluation, Center for Veterinary Medicine,
Food and Drug Administration

Dr. Qiao Zhang is a Mathematical Statistician in the Office of New Animal Drug Evaluation in the FDA Center for Veterinary Medicine (CVM). She received her doctorate degree in Statistics at Stony Brook University in Long Island, New York. In CVM, Dr. Zhang works as a statistical reviewer using many types of statistical procedures with comprehensive biomedical knowledge in performing reviews and evaluations for evidence of safety and effectiveness of investigational new animal drugs. Her research area of interest is adaptive and innovative study design and its applications in pharmaceutical product development.

Dr. Michele J Sharkey Memorial PAW Lecture

Outcome Assessment in Veterinary Pain Studies: The Yellow Brick Road Continues

Dottie Cimino Brown MS, DVM, DACVS
Vice President Science & Healthcare Innovation
Mars Veterinary Health

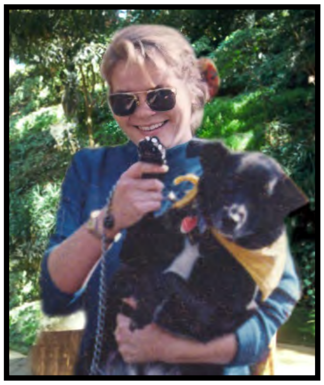

This first Dr. Michele J Sharkey memorial lecture will recognize Michele as a key figure in the success of this Pain in Animals Workshop (PAW). Since its inception, Michele was the glue that held the PAW planning committee together. She was phenomenal at ‘herding cats’, but more importantly, she always kept us focused on our mission. She kept us honest in our commitments and challenged us to think outside the box. She connected individually with everyone in only the most positive and uplifting ways. Recognizing how important Michele has been in our journey to find better ways to measure and manage pain in animals, we will look back at how far we have come from that first workshop in 2017.

PAW 2017: Creating a Roadmap for Measuring Chronic Pain in Dogs & Cats

The first workshop discussed the status of chronic pain measurement in companion animals from the perspectives of therapeutic drug development & translational research. It touched on the predominant methods of outcome assessment including clinical metrology instruments, gait analysis, and activity monitoring, as well as the potentially valuable approaches of quantitative sensory testing and nociceptive withdrawal reflex. The impact of placebo effects on pain outcome measures was also addressed. It was a broad list of topics from which an extensive list of priorities for future research was generated. We will look back on those priorities and see where progress has been made and where more focus is needed.

PAW 2019: Cross-Species Measurement of Acute Pain

The second workshop broadened the discussion to include farm animal species, discussing the advances and roadblocks to the measurement of acute pain and the potential for spontaneous conditions in animals to contribute to translational acute pain research for human therapeutics. Both subjective and objective measures were discussed including questionnaires, facial expression scales, activity monitoring, kinetic limb evaluation, complex behavioral tests, and physiological measures across species. The impact of placebo effects on outcome was again discussed and animal welfare in pain studies was an additional focus. We will look back at the list of priorities for future research that was generated during this workshop and see which areas have robust activity.

PAW 2021: Exploring Best Practices & Knowledge Gaps for Objectively Measuring Chronic Pain

The third workshop drilled down on topics presented in the prior meetings to highlight new knowledge and continued priorities for future research. The discussions focused on gait analysis - establishing best practices for measuring and reporting ground reaction forces; and accelerometry - critiquing its use in evaluating osteoarthritis pain in companion animals, as well as acute pain in cattle. In addition, a new topic was also introduced, the potential for the use of cell-based and transcriptome biomarkers for the objective evaluation of pain. We will highlight some of the progress made on these initiatives since this meeting just two years ago.

Measurement Properties of Pain Scoring Instruments in Farm Animals

Marina Cayetano Evangelista, MV, MSc, PhD

Measurement properties refer to the characteristics or attributes of an instrument which are a consequence of the methodology used in their respective studies. They often refer to the quality aspect of an instrument (i.e.: reliability, validity and responsiveness). In the literature, different terminology and definitions for measurement properties are continuously being used. The nomenclature used herein follows the taxonomy of measurement properties relevant for evaluating outcome measure instruments developed by the COSMIN initiative[1].

A recent systematic review[2] assessed nine categories of measurement properties in farm animals' pain scales: two for scale development (general design requirements and development, content validity and comprehensibility) and seven for measurement properties (internal consistency, reliability, measurement error, criterion and construct validity, responsiveness, and cross-cultural validity).

The most studied species are bovine, ovine and swine, which together comprise 20 instruments included in the systematic review. There was considerable variability concerning their development and measurement properties. Twelve behavior-based scales were evaluated, including six for bovine (beef and dairy cattle): Unesp-Botucatu Unidimensional Composite Pain Scale for assessing postoperative pain in cattle (UCAPS), Posture Scoring System, Multidimensional Pain Scoring System, *Escala Composta Análogo-Visual*, Veterinarian Pain Scale and Technician Pain Scale; three for ovine: Pain Scoring System for Ventricular Assist Devices-Implanted Sheep, Behavior Assessment Scheme, Unesp-Botucatu Composite Scale to Assess Acute Postoperative Abdominal Pain in Sheep (USAPS); and three for porcine: Unesp-Botucatu Pig Composite Pain Scale (UPAPS), Perception of Pain, Distress and Discomfort Assessment and Behavioral Pain Scale in Piglets. Additionally, seven facial expression/grimace scales including one for bovine: Pain Assessment Based on Facial Expression; three for ovine: Sheep Pain Facial Expression Scale (SPFES), Sheep Grimace Scale (SGS) and Lamb Grimace Scale (LGS); and three for porcine: two named Piglet Grimace Scale (PGS-A and B) and Sow Facial Expression Scale. The Cow Pain Scale is composed by facial expressions and behaviors for dairy cattle.

Only three behavior-based instruments scored high for strength of evidence: UCAPS (cattle), USAPS (sheep) and UPAPS (pigs). Other four instruments scored moderate for strength of evidence: MPSS (bovine), SPFES (sheep), LGS (lamb) and PGS-B (piglets). Most instruments included in the systematic review scored low or very low for final overall strength of evidence.

The most reported measurement property was construct validity, followed by criterion validity and reliability. In addition to an increase in the use of the available scales, instruments with reported validation are urgently required for pain assessment of other species such as buffalos, goats, camelids, and poultry.

References

[1] Mokkink LB, Terwee CB, Patrick DL, Alonso J, Stratford PW, Knol DL, Bouter LM, de Vet HC. The COSMIN study reached international consensus on taxonomy, terminology, and definitions of measurement properties for health-related patient-reported outcomes. J Clin Epidemiol. 2010; 63(7):737-45. doi: 10.1016/j.jclinepi.2010.02.006.

[2] Tomacheuski RM, Monteiro BP, Evangelista MC, Luna SPL, Steagall PV. Measurement properties of pain scoring instruments in farm animals: A systematic review protocol using the COSMIN checklist. PLOS ONE. 2021; 16(5): e0251435. https://doi.org/10.1371/journal.pone.0251435

Biomarker Update: What Progress Have We Made?

Daniel Barratt, BSc(Hons), Phd
University of Adelaide.

Objective biomarkers of pain can help assess the impacts of production practices on animal wellbeing and support analgesic drug development and approvals in livestock species.

Increased understanding of the role of peripheral immune cells in pain, and of gene expression changes in response to noxious stimuli, have prompted exploration of novel blood-based transcriptomic biomarkers of pain and analgesia. As key regulators of gene expression that have shown potential as minimally invasive biomarkers in other diseases, circulating microRNAs (miRNAs) have attracted particular interest, with candidate miRNA biomarker discovery studies utilising existing data and small RNA sequencing of serum exosomes from analgesic trials in cattle and pigs producing promising results.

In male Holstein calves undergoing sham procedure, or dehorning and castration with or without analgesia (meloxicam±lidocaine), we identified serum exosome miRNAs with temporal expression profiles closely matching those of behavioural pain scores, five of which demonstrated ‘outstanding’ performance (area under receiver operating characteristic curve (AUROC) >0.9) in distinguishing ‘pain’ from ‘no pain’ timepoints within calves. Differential gene expression analyses also identified additional ‘rapid’ (6 hours post-procedure) and ‘delayed’ (96+ hours post-procedure) response miRNAs, some of which indicated “reversal” with meloxicam.

In a trial of transmammary firocoxib and topical ethyl chloride in piglets undergoing tail docking (and castration for males), multiple serum exosome miRNAs were significantly up- or down-regulated 24 hours following docking/castration in control (no analgesic) piglets, with AUROCs ranging 0.71 (acceptable) to 1 (perfect) for differentiating pre- versus post-procedure. A subset of miRNAs also suggested “reversal” with firocoxib. However, these encouraging findings must be interpreted with caution due to the absence of a sham control (no docking/castration).

The critical work of replicating these initial discoveries (with appropriate controls) is underway. Complementary research in sheep is also in progress, examining blood total RNA transcriptome and epigenetics, as well as miRNAs, alongside end-of-life brain and spinal cord histology and protein analyses. In addition to biomarker discovery, this work is helping to build an integrated picture of the short- and long-term molecular, cellular, systems and behavioural consequences of painful marking procedures.

Trials employing standard husbandry procedures (docking/castration/dehorning) and veterinary-relevant analgesic approaches, with their high ecological validity, have rightly formed the basis of this biomarker discovery work. One challenge with this approach moving forward is distinguishing biomarkers of pain *per se* from biomarkers of tissue damage and wound healing; complementary studies using alternative pain models and/or analgesic approaches may be required to specifically address this.

Title: Veterinary Medicine and Artificial Intelligence: Do Androids Dream of Electric Sheep in Pain?

Artificial intelligence (AI) is revolutionizing the way we live and work. From search tools, recommender systems, personal assistants, fraud detection, and automated systems, the reach of AI systems seems ubiquitous. The impact of AI in veterinary medicine is similarly growing, and it has the potential to become an essential tool for companion animal health, livestock health, and population medicine. With the advent of AI, veterinarians will have access to powerful algorithms and machine-learning tools that may help them make more accurate and timely diagnoses. AI can also analyze large volumes of data, including medical records, diagnostic tests, and imaging studies, to identify patterns and trends, which may enable veterinarians to provide more personalized and effective treatment plans for their patients. By collecting and analyzing data from multiple sources, including animal health records, environmental data, and social media, AI can help identify disease outbreaks and inform public health policies. This can be especially useful for managing zoonotic diseases that can spread from animals to humans and vice versa. AI is also transforming agricultural practices where farmers can monitor the health and well-being of their livestock, identify potential health issues early on, and optimize feeding and breeding practices. This can lead to improved animal welfare and increased productivity, which can benefit both farmers and consumers.

As ongoing research and commercial advancements in AI continue to unfold, integrating this technology into veterinary practice brings both opportunities and challenges. While AI has the potential to improve animal health and welfare, it also raises ethical concerns about data privacy and transparency challenges, and bias.

In this presentation, we will first provide a basic introduction to artificial intelligence, with a focus on computer vision, natural language processing, and speech recognition as they relate to veterinary medicine. Second, we will present examples of AI within veterinary medicine in the commercial and research spaces. We will also describe how AI technologies could be used to predict pain scores in animals, how such systems might work, and what datasets and AI architectures could be used. Finally, we will discuss the strengths and challenges of using AI in veterinary medicine.

The promises of AI must be balanced with reality: Too often, these ubiquitous AI systems have demonstrated biases with serious deleterious effects on society. While computational techniques can be quickly and economically borrowed from human-centric applications, critical differences, and challenges unique to veterinary medicine must be noted, such as data privacy, the interpretations of outputs, and its relationship with patient care -most notably euthanasia- and the heterogeneity of data given variations in breeds and socio-economic factors. This presentation will also briefly discuss these important considerations when developing trustworthy AI in veterinary medicine.

Assessing pain in pigs: A collaborative effort by industry, academia, and government to advance pig welfare

Sherrie R. Webb[1], Angela Baysinger[2], Jennifer Brown[3], Hans Coetzee[4], Neil Cox[5], Ashley DeDecker[6], Locke Karriker[7], Janis Messenheimer[8], Michael Myers[8], Monique Pairis-Garcia[9], Virginia Recta[8], Claire Ruberman[8], Emily Smith[8], Mhairi Sutherland[10], Abbie Viscardi[4], Stephanie Wisdom[11]

[1]American Association of Swine Veterinarians; [2]Merck Animal Health; [3]Prairie Swine Centre; [4]Kansas State University; [5]NeilStat, LLC; [6]Smithfield Foods; [7]Iowa State University; [8]US Food and Drug Administration, Center for Veterinary Medicine; [9]North Carolina State University; [10]Beef + Lamb New Zealand; [11]National Pork Board

Surgical castration of male piglets is performed to prevent off-odor pork and aggressive behaviors. In the United States, the procedure is often performed without analgesics or anesthetics in part due to the lack of US FDA (FDA)-approved drugs with an indication for the control of pain in swine. Additionally, pain control recommendations based on published studies are limited by inconsistencies in study design and outcomes, and a lack of well-defined and reliable endpoints for assessing pain in piglets. These factors create difficulty for pharmaceutical companies to design appropriate studies to demonstrate the substantial evidence of effectiveness needed to support an approval of a new animal drug with a pain control label indication, veterinarians to confidently prescribe FDA-approved products for extra-label use in accordance with the Animal Medicinal Drug Use Clarification Act, researchers to reliably assess pain and potential mitigation strategies, and pig farmers to address animal welfare and make future business decisions. In response to these challenges, a consortium of industry representatives, academicians with expertise in pain assessment, and representatives from the FDA was established. The consortium's primary goal is to conduct well-controlled studies to validate behavioral, physiologic, and biomarker-based outcomes and endpoints, and associated decision criteria that could be used in future clinical studies to demonstrate effectiveness of a drug(s) to control pain associated with surgical castration of nursing male piglets. With funding from USDA-NIFA-AFRI and the swine industry, the consortium team is working to validate various endpoints including a behavioral pain scale, piglet grimace scale, infrared thermography, gait scoring using a pressure mat, cortisol, prostaglandin metabolites, and microRNA. An additional outcome of the research is to develop a standardized research protocol that includes standard operating procedures for each of the validated endpoints to facilitate consistency, reproducibility, and rigor in future research. Used together, the validated endpoints and standardized research protocol will provide a regulatory pathway for pharmaceutical companies to develop protocols for studies to evaluate the effectiveness of therapeutic products for pain management in nursing male piglets. This also will assist researchers, veterinarians, and pig farmers in evaluating drug efficacy and developing consensus on best practices for pain management.

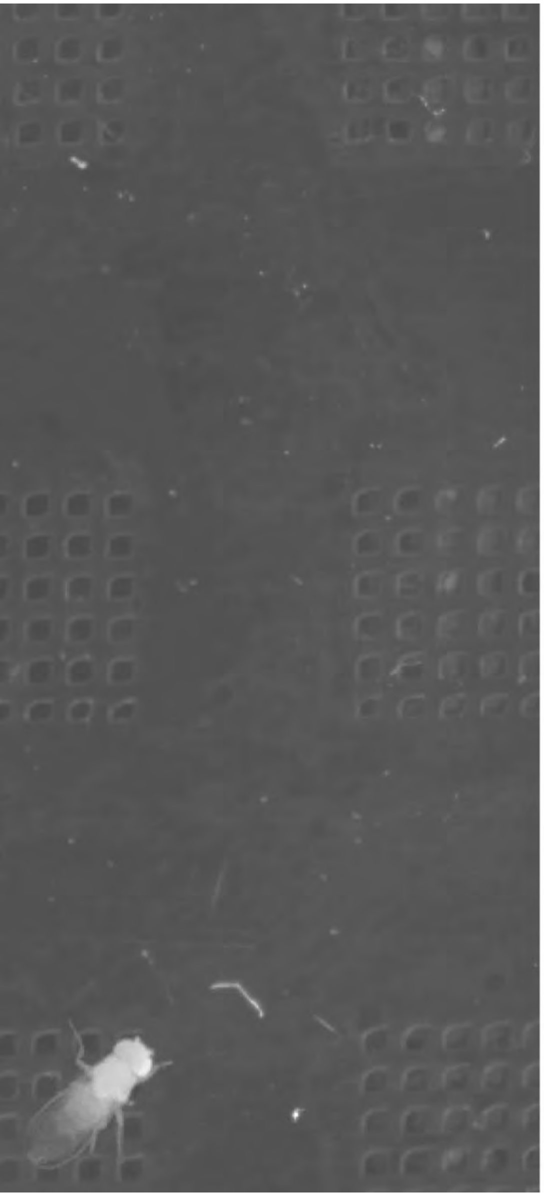

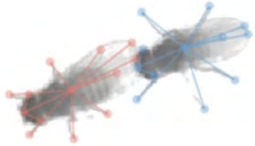

Recent Advances in Rodent Pain Measurement

Automated Detection of Behaviors and Application of Deep Learning

PAW 2023

Liezl Maree, MSc
Scientific Programmer
Salk Institute for Biological Studies
https://talmolab.org

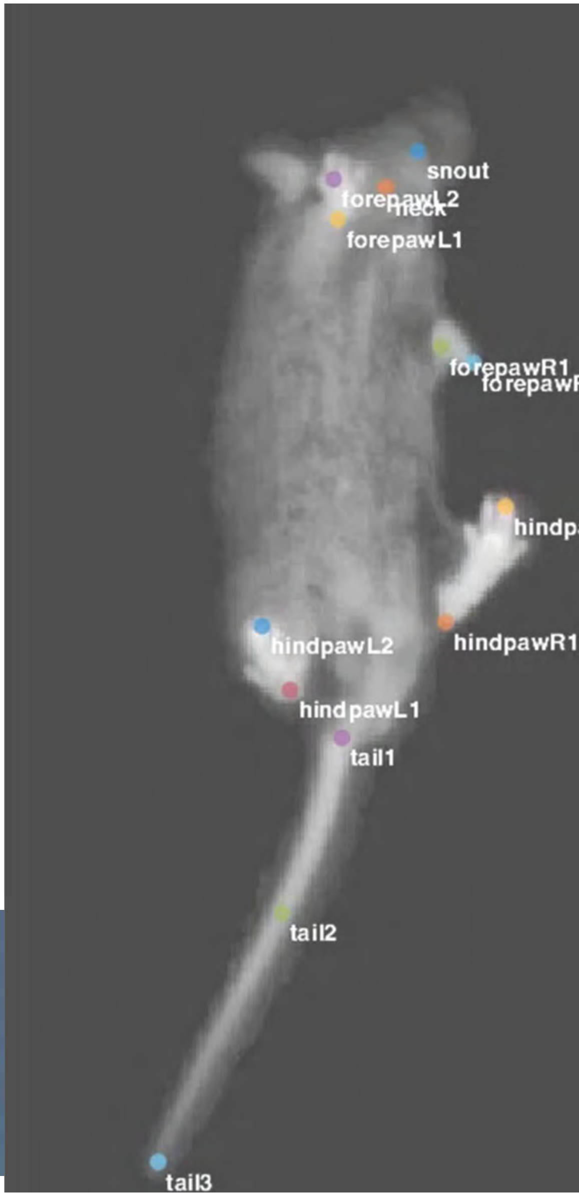

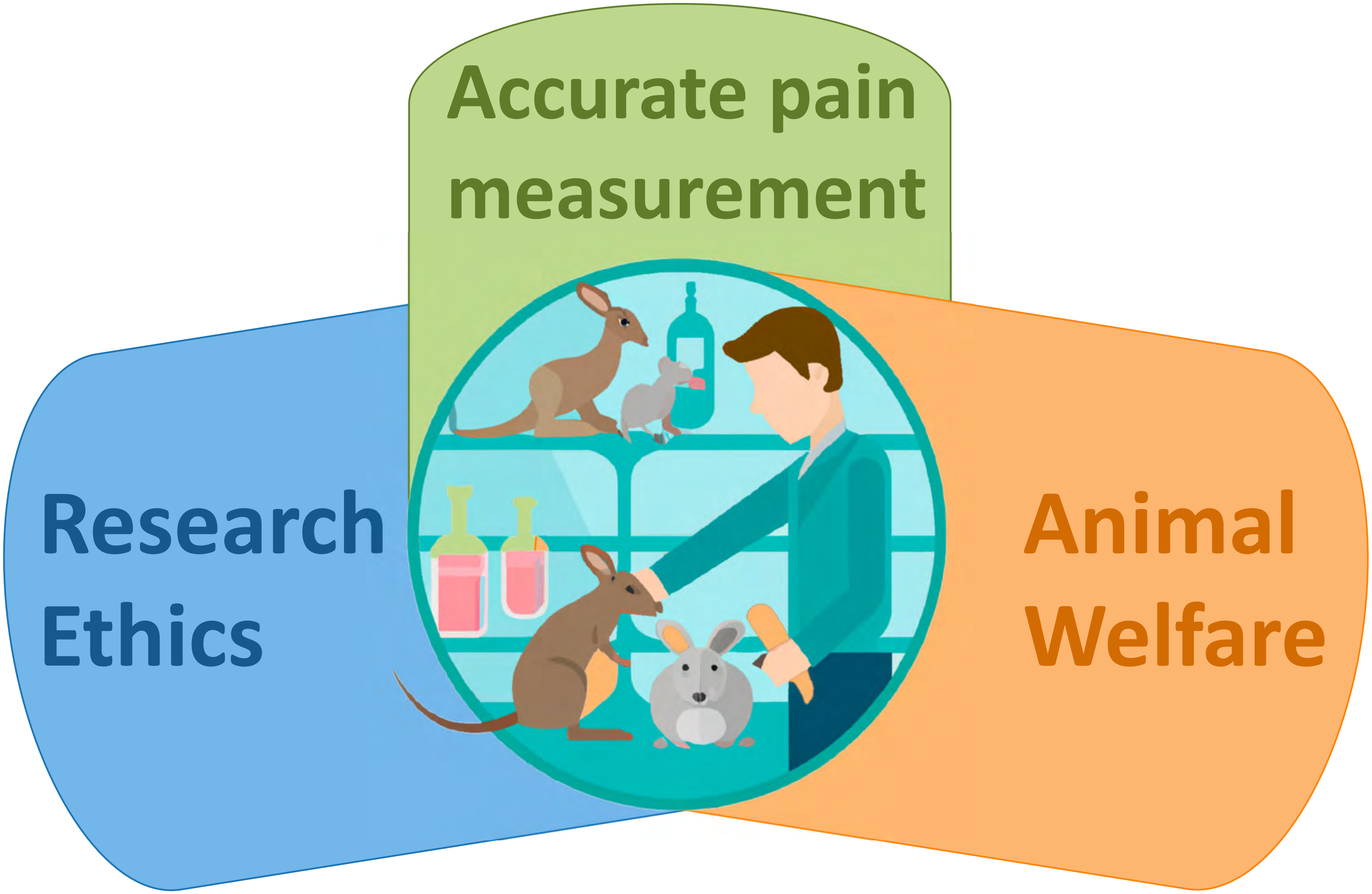
Accurate pain measurement
Research Ethics
Animal Welfare

Limitations of traditional methods: **subjectivity**

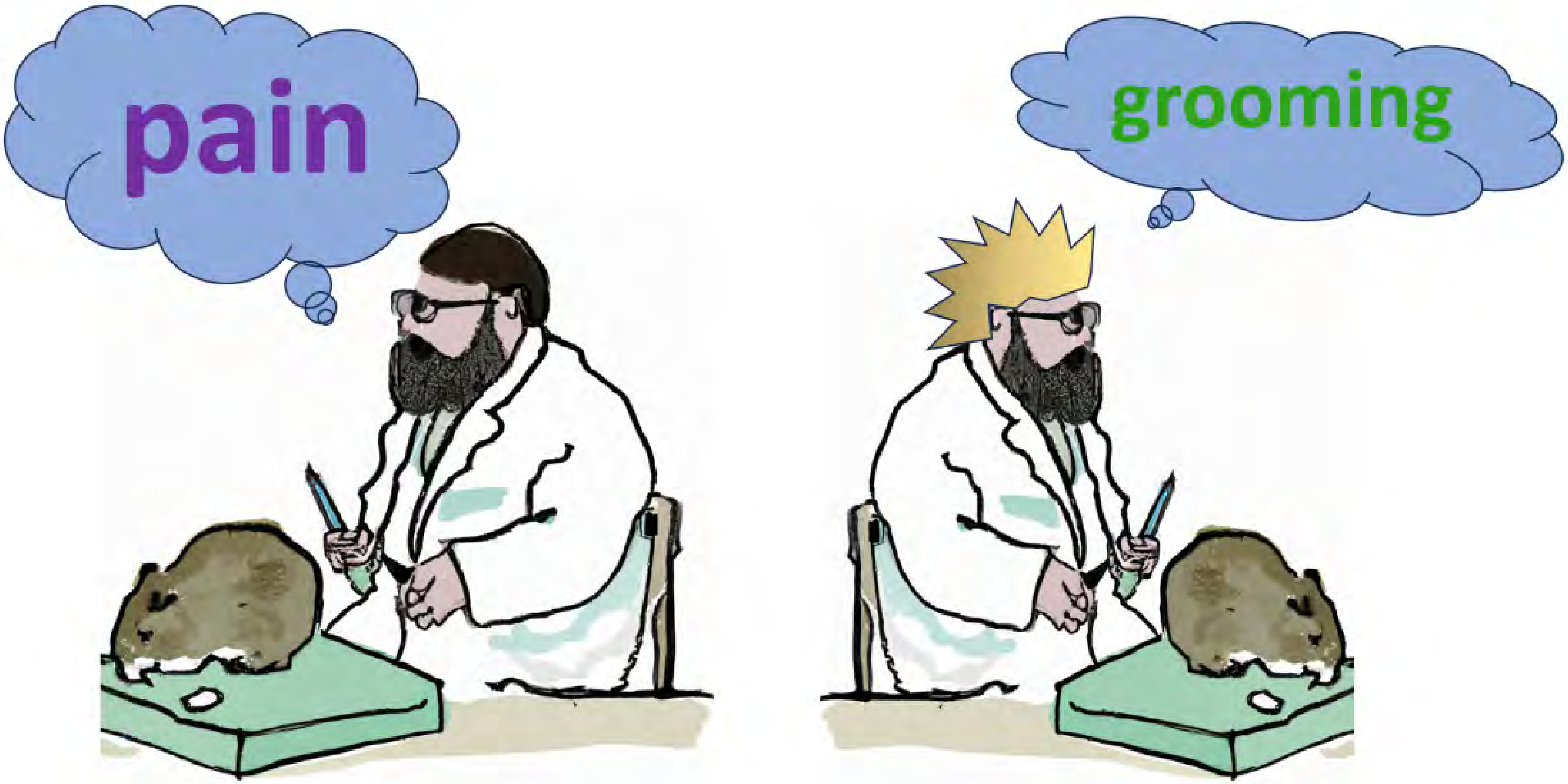

Limitations of traditional methods: **disruption**

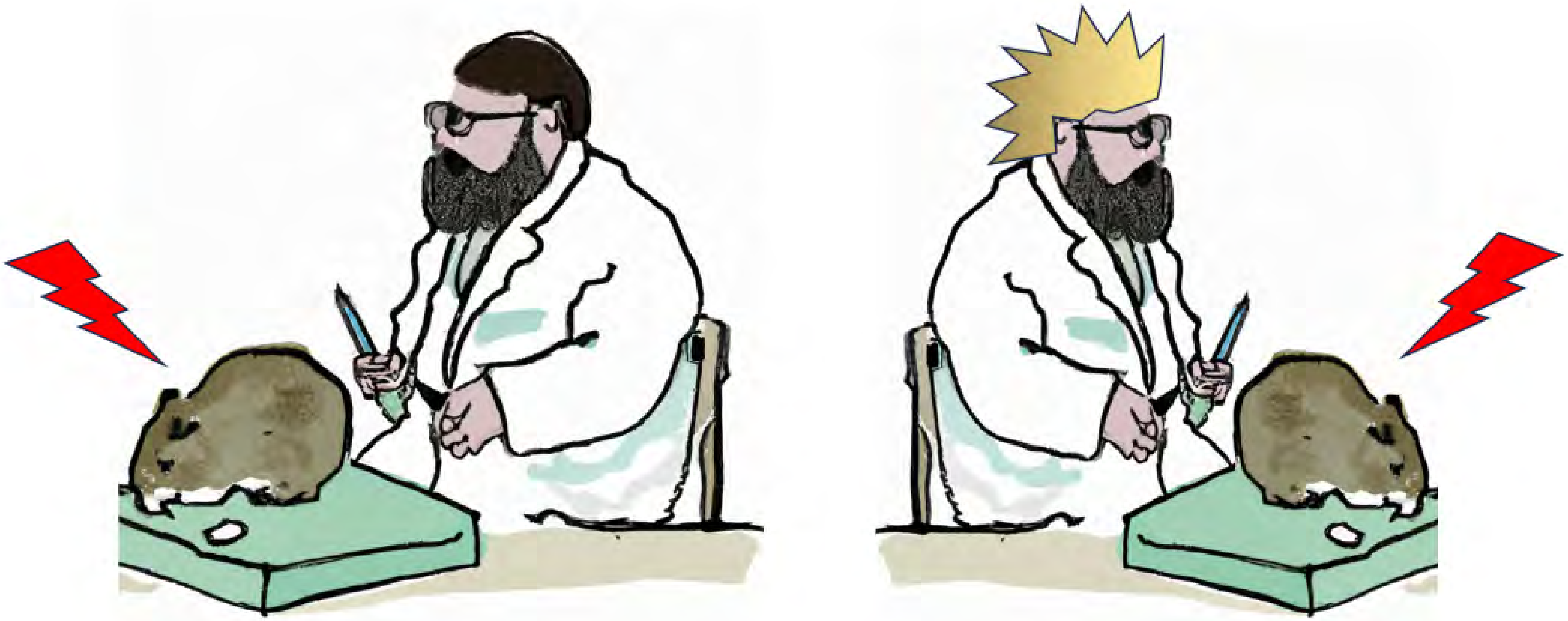

Limitations of traditional methods: **time**

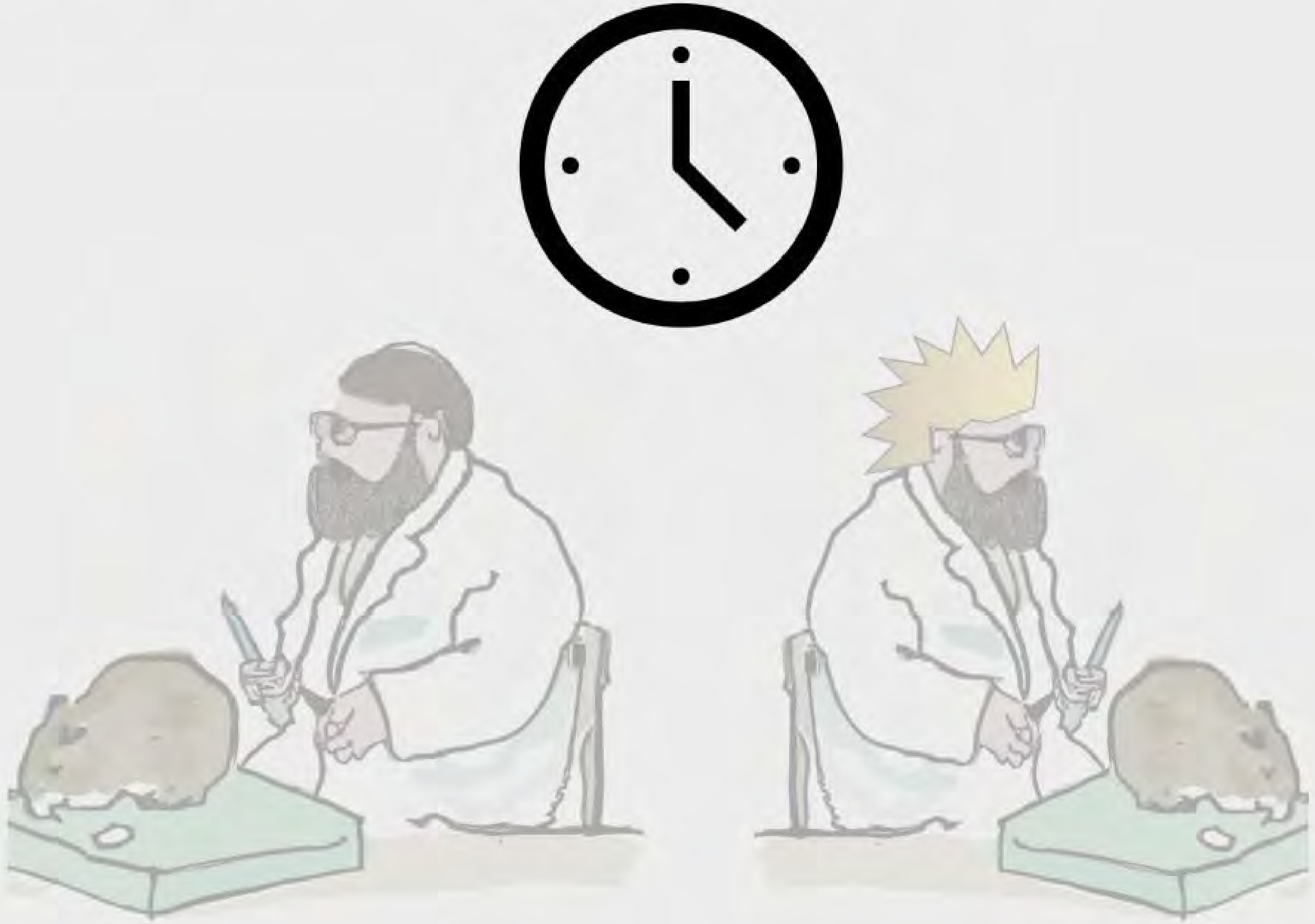

if d < 5

then “paw lick”

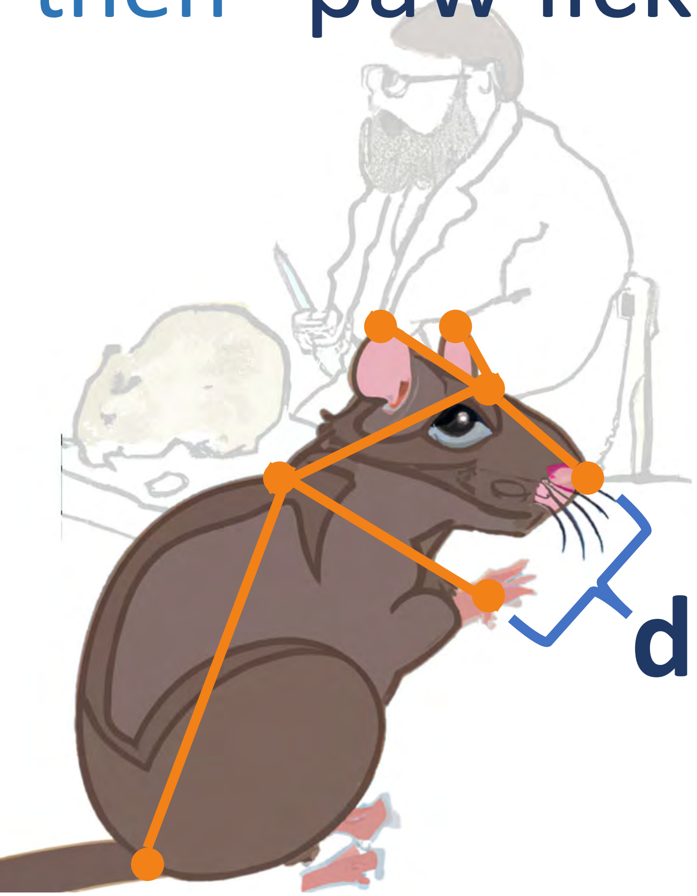

or behavioral classification

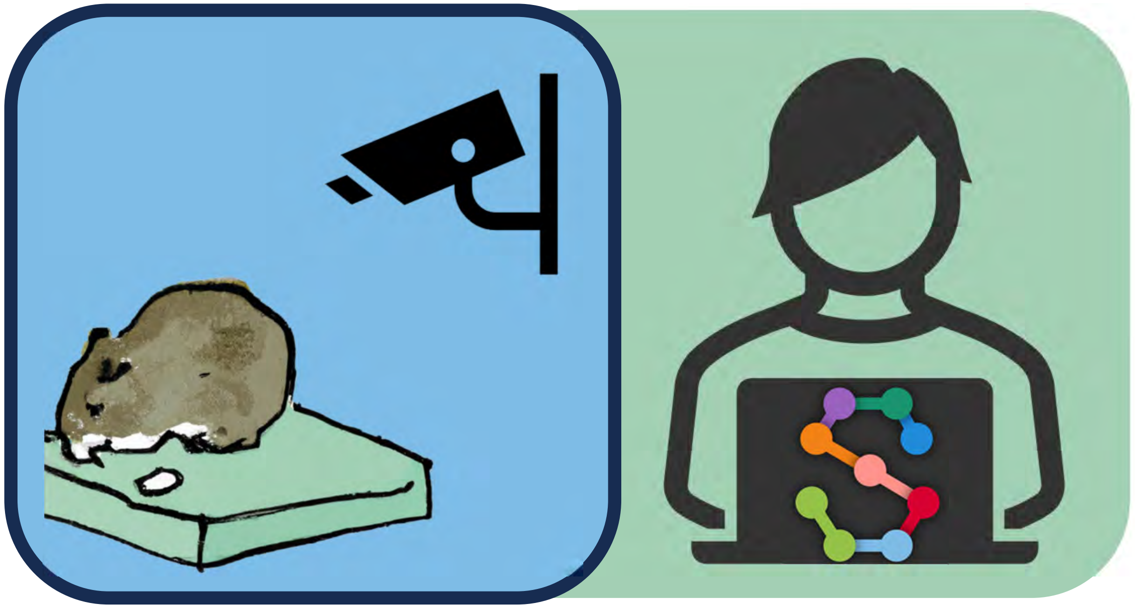

Quantitative analysis

Engineering AI to capture biological motion

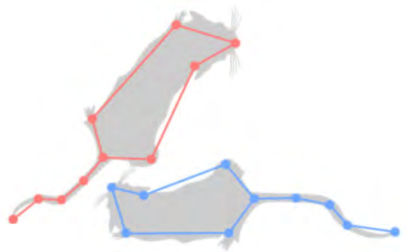

Quantifying **biological motion** from coarse to fine

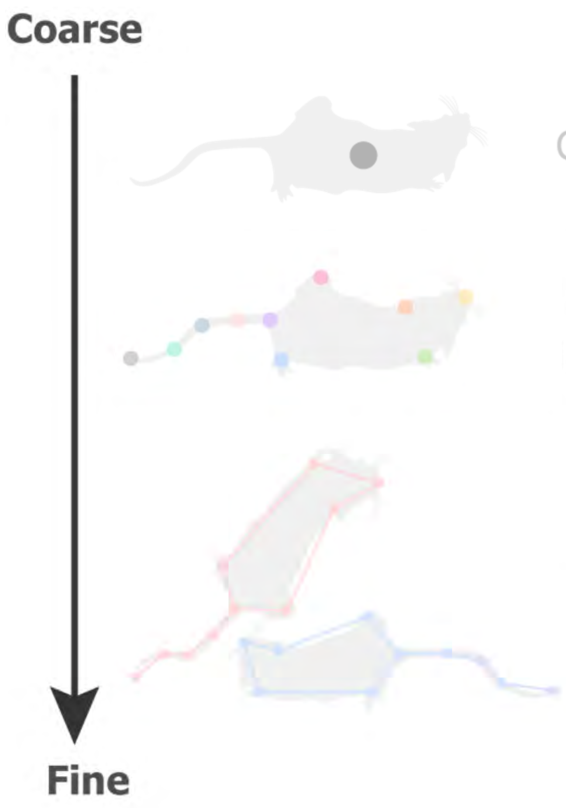

Pereira et al., *Nature Neuroscience* (2020)

Navigation

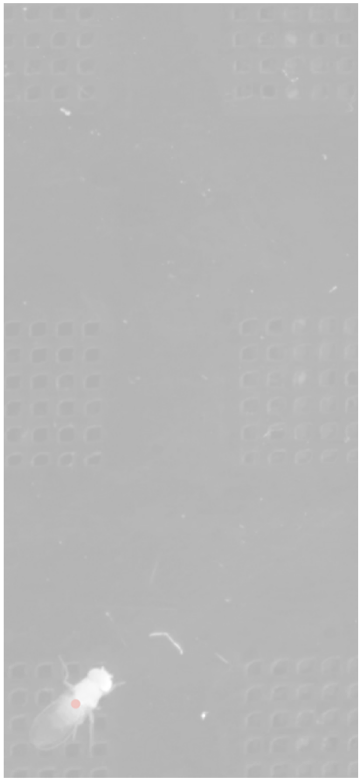

Quantifying **biological motion** from coarse to fine

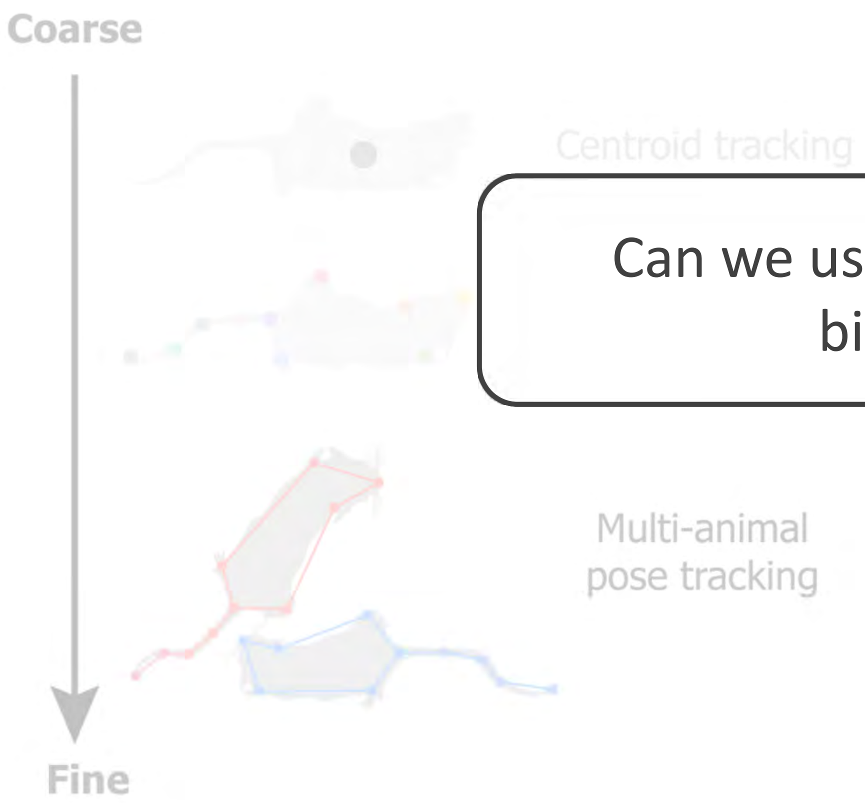

Social behavior

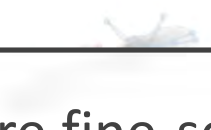

Can we use AI to capture fine-scale biological motion?

Pereira et al., *Nature Neuroscience* (2020)

Social LEAP Estimates Animal Poses (SLEAP)

Pereira et al., *Nature Methods* (2019): LEAP → Pereira et al., *Nature Methods* (2022)

Docs: sleap.ai | **Code:** github.com/talmolab/sleap

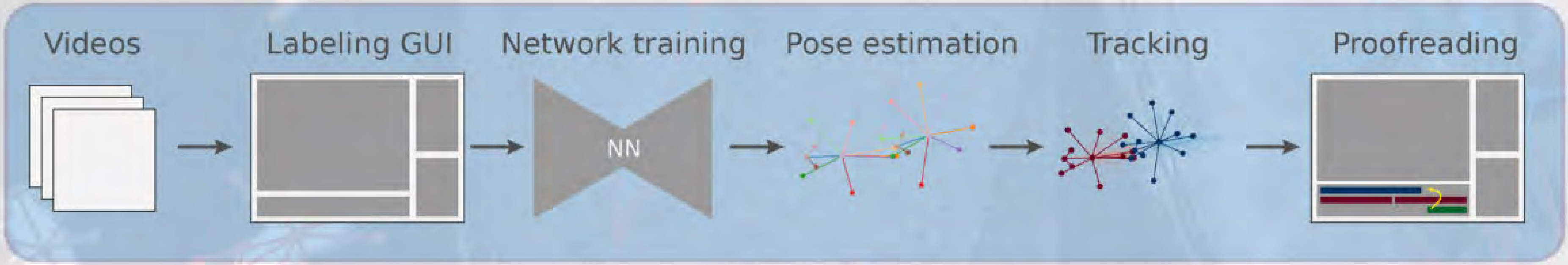

Social LEAP Estimates Animal Poses (SLEAP)

Pereira et al., *Nature Methods* (2019): LEAP → Pereira et al., *Nature Methods* (2022)

Docs: sleap.ai | **Code**: github.com/talmolab/sleap

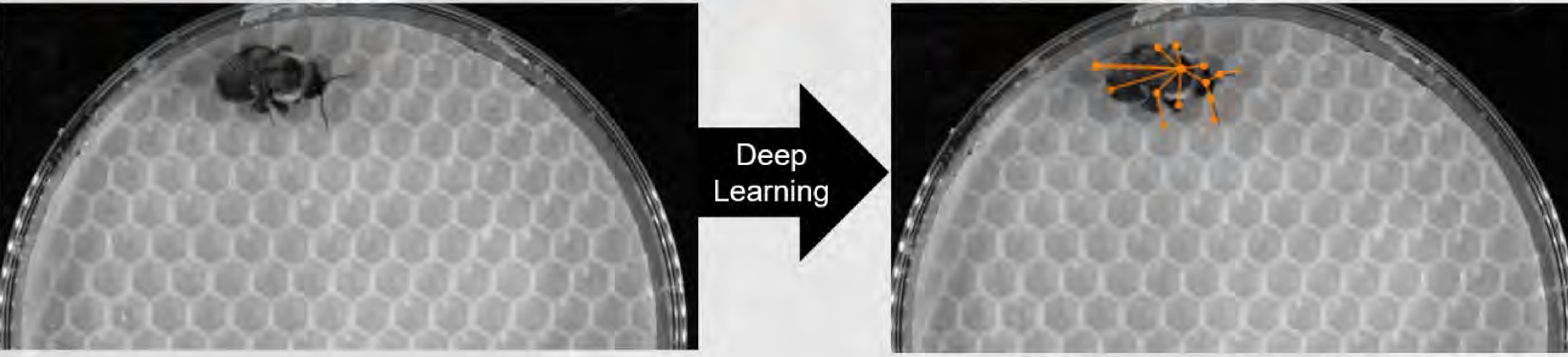

Engineering team (Princeton):
Nat Tabris Dave Turner Arie Matsliah

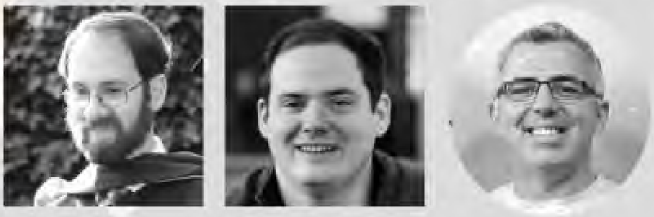

Engineering team (Salk):
Shrivaths Shyam Liezl Maree Divya Murali

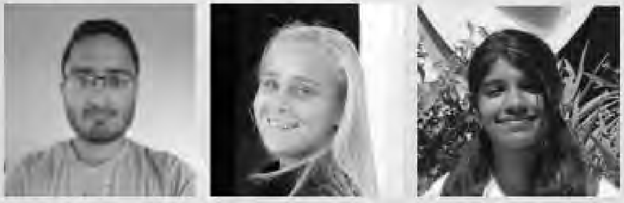

User base:
50,000+ downloads, 400+ citations
15+ countries, 60+ institutions including:
Salk, Princeton, Harvard, MIT, Stanford, Yale, Cornell, University c
Pennsylvania, Columbia, NYU, UCSD, UCLA, UC Berkeley, U of
Oregon, University of Washington, MathWorks, Emory University
Okinawa Institute of Science and Technology (Japan), Weizmann
Institute of Science (Israel), Hebrew University (Israel), European
Neuroscience Institute (Germany), Max Planck Institute for Brain
Research (Germany), UKB University of Bonn (Germany), ...

SLEAP: ID via temporal association (Flow shift)

- ✓ **No labeling or training required**
- ✓ Works with **visually indistinguishable animals**
- ✗ **Propagates errors** over time

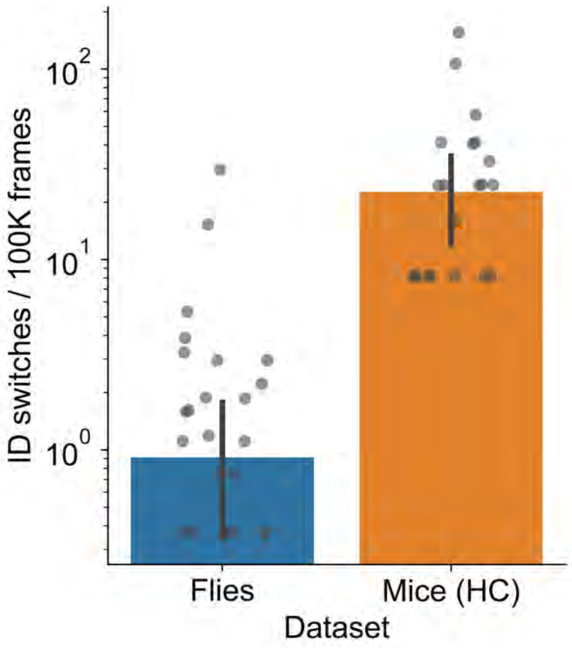

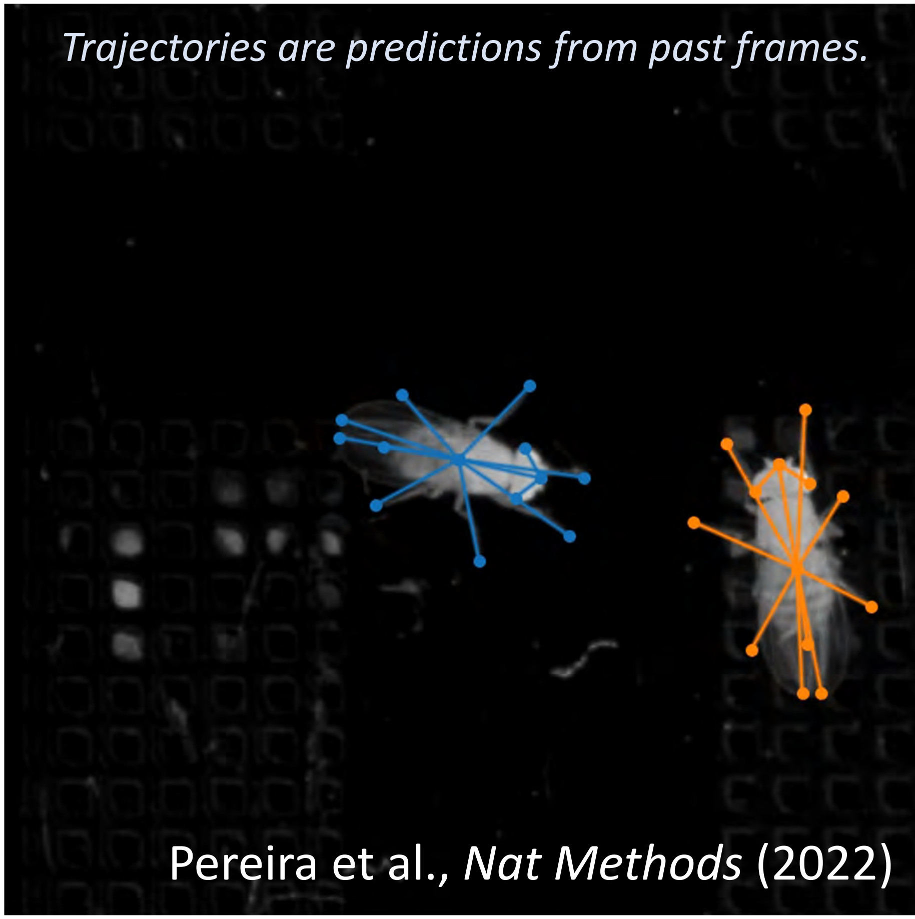

SLEAP: ID via direct classification

- ✓ **Does not propagate errors** (>99.5% Acc)
- ✓ **Low latency** for online tracking (<10-15 ms)
- ✗ Less accurate with **visually similar animals**
- ✗ **Requires labeling** identities

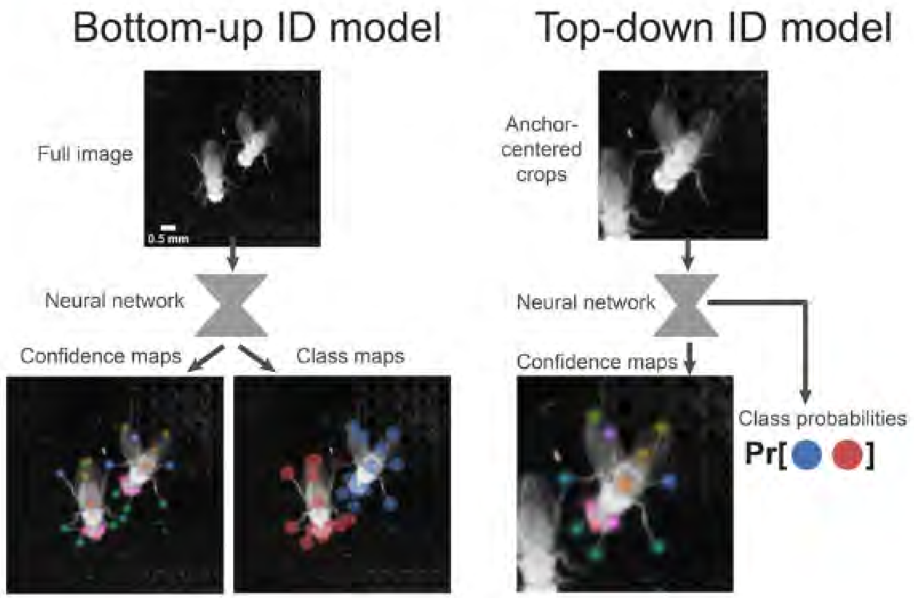

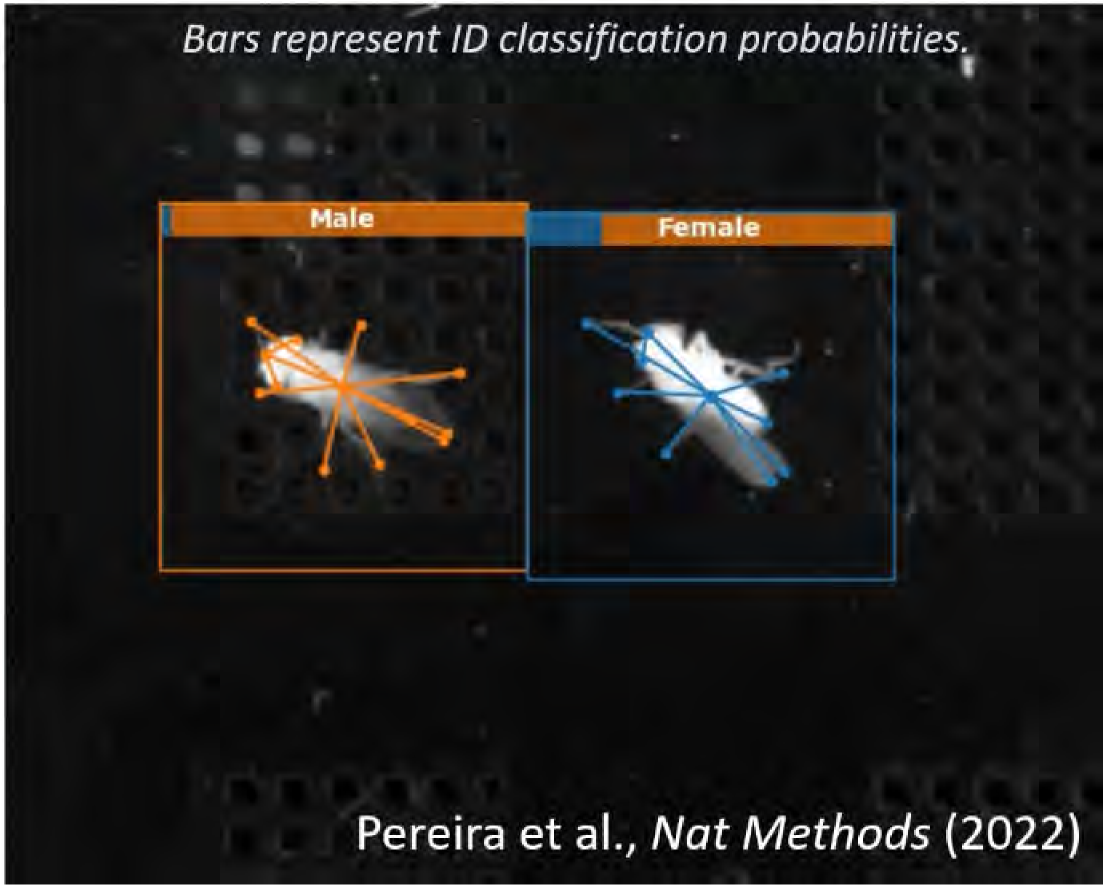

Pereira et al., *Nat Methods* (2022)

Multi-animal behavior in 3D: The simple approach

w/ Lili Karashchuk
Bing Brunton

Multi-animal poses in 2D

Multi-view triangulation

SLEAP
Pereira et al. (2022)

+

Anipose
Karashchuk et al. (2021)

→ **Multi-animal 3D?**

github.com/talmolab/sleap-anipose

Sean Afshar (Princeton)
Stefan Oline (Princeton)
Annegret Falkner (Princeton)

Multi-animal behavior in 3D: The simple approach?

Problem: Some views are harder than others in 2D…

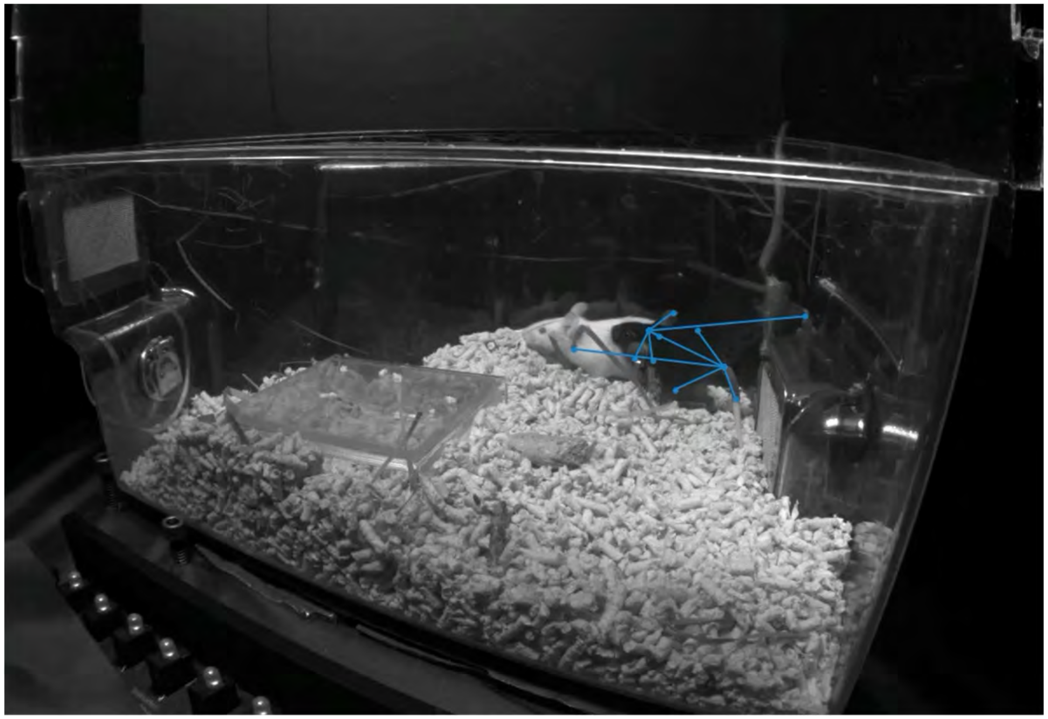

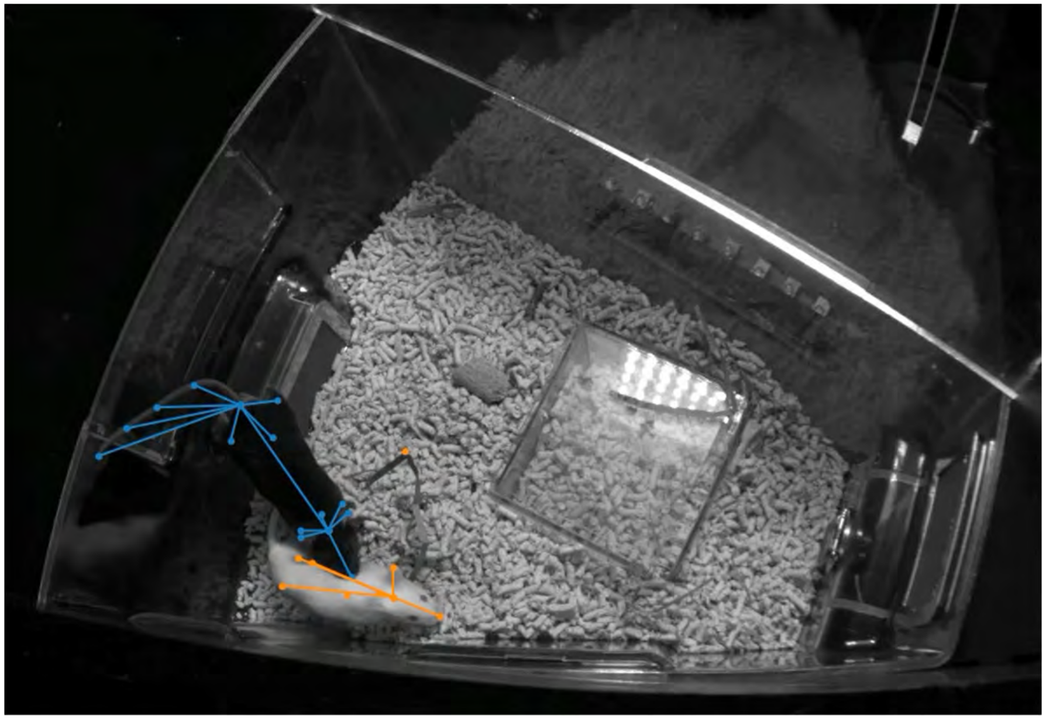

Sean Afshar (Princeton)
Stefan Oline (Princeton)
Annegret Falkner (Princeton)

Multi-animal behavior in 3D: Held-out view

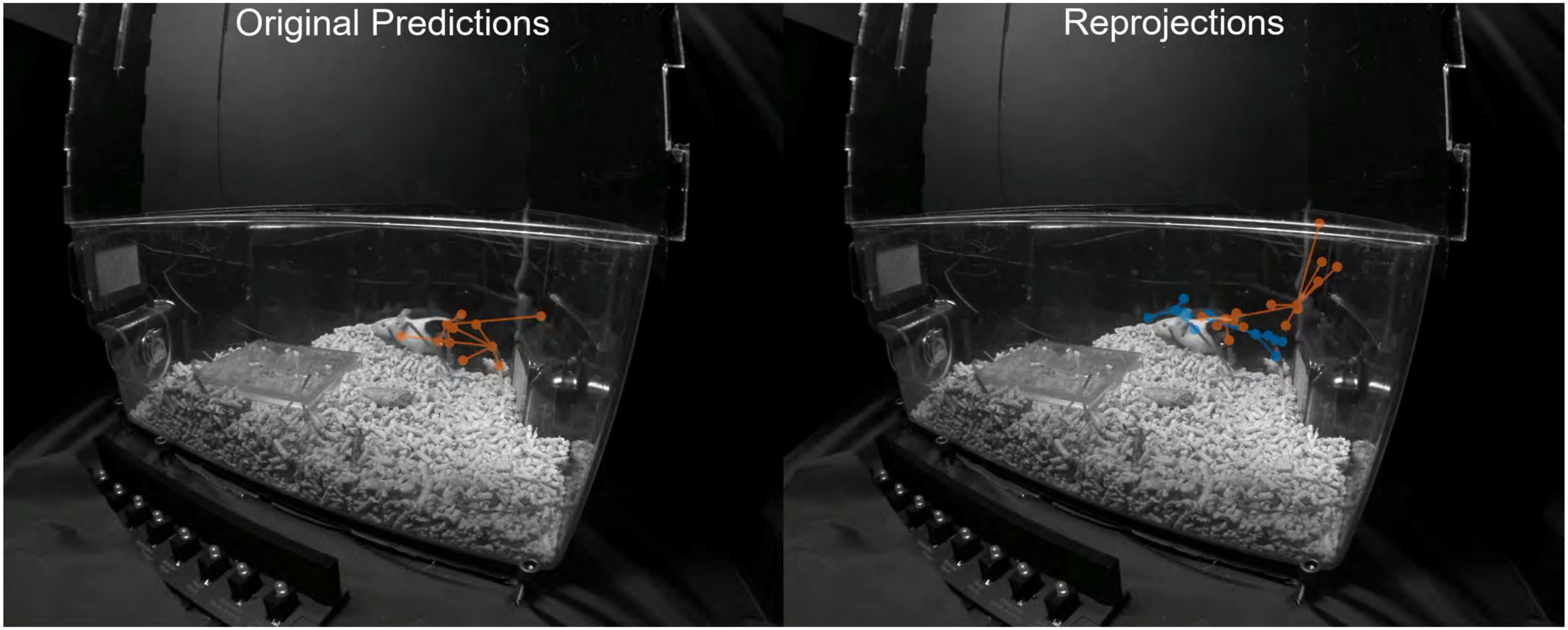

Sean Afshar (Princeton)
Stefan Oline (Princeton)
Annegret Falkner (Princeton)

Multi-animal behavior in 3D

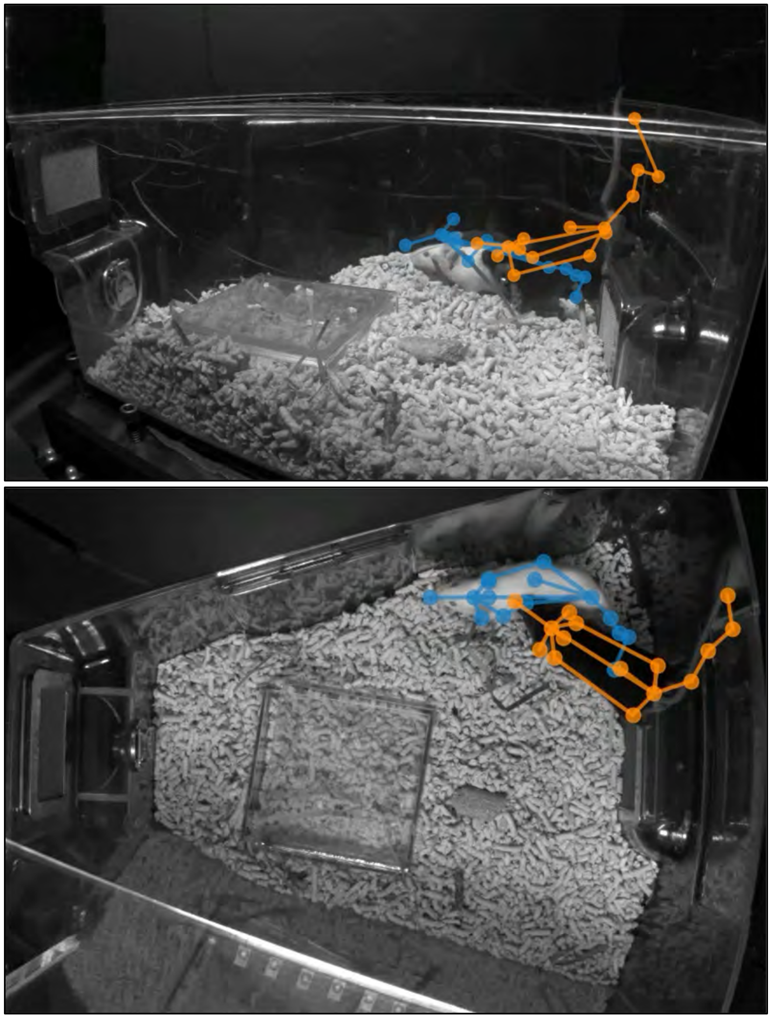

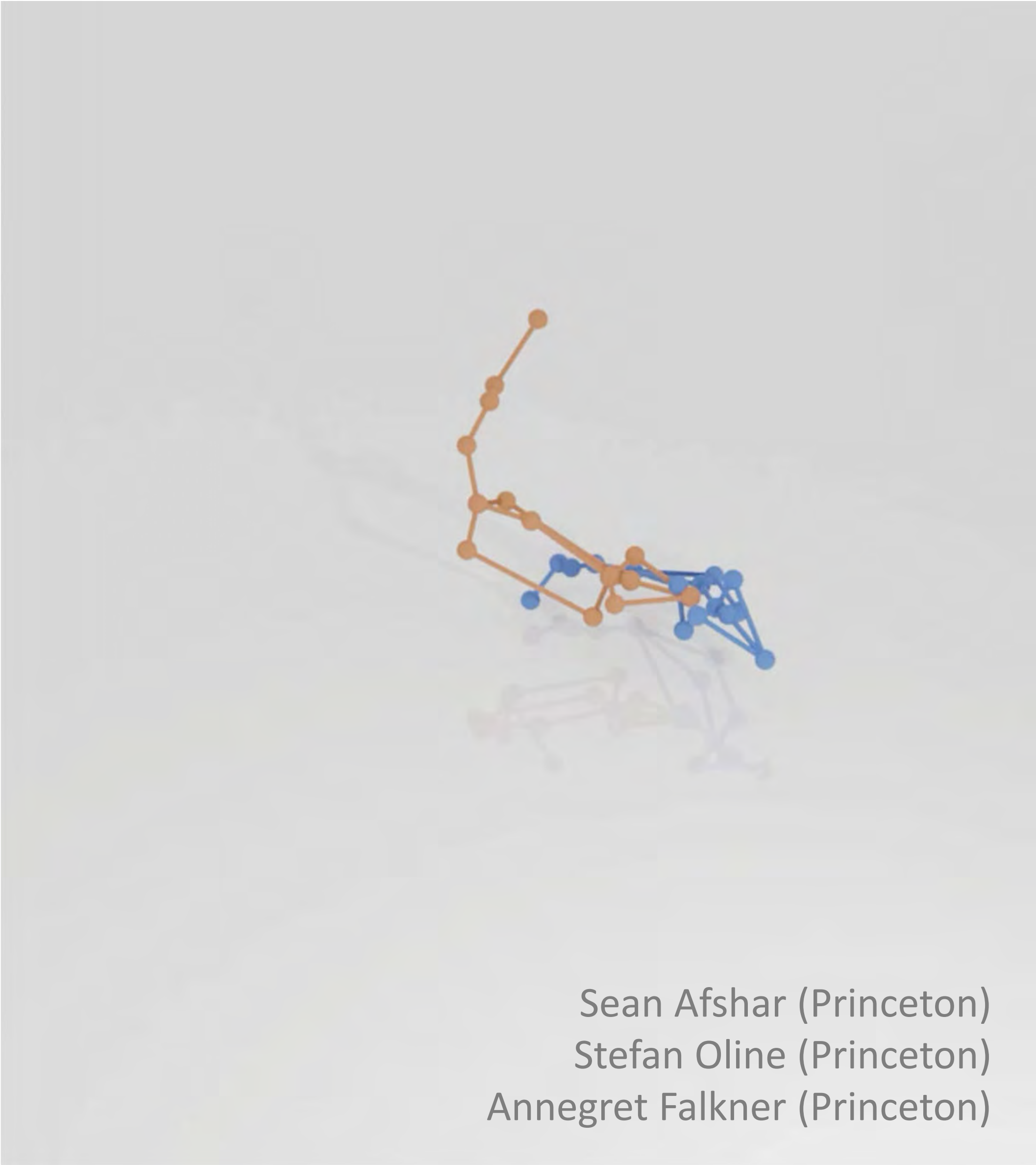

Thanks for listening!

Talmo Pereira, PhD
Salk Fellow & Principal Investigator
Salk Institute for Biological Studies
https://talmolab.org
@talmop

Murthy Lab (Princeton)
Diego Aldarondo (now at Harvard)
Junyu Li
Shruthi Ravindranath
Nat Tabris (now at Coiled)
Arie Matsliah
Dave Turner
Eleni Papadoyannis (rotation)
David Deutsch (now at Haifa University)
Jan Clemens (now at ENI Göttingen)
Fred Roemschied
Edna Normand
Sama Ahmed (now at UW)
Diego Pacheco Pinedo
Christa Baker

Shaevitz Lab (Princeton)
Ugne Klibaite (now at Harvard)
Gordon Berman (now at Emory)
Grace McKenzie-Smith
Scott Wolf
Silke Bergeler
Ben Bratton

Collaborators
Sam Wang
Mikhail Kislin
John D'Uva (JHU)
Annegret Falkner
Lindsay Willmore
Mae Guthman
Stefan Oline
Dakota Blackman
Sara Kocher
Z. Yan Wang
Dave Turner
Dan Sanes (NYU)
Catalin Mitelut (NYU)
Lisa Diez (NYU)
Jens Schweihoff (University of Bonn)
Gabriella Gall (Tel Aviv University)
Yasmine Meroz (Tel Aviv University)
Nick Andrews (Salk)
Wolfgang Busch (Salk)
Elizabeth Berrigan (Salk)
Uri Manor (Salk)
Kay Tye (Salk)
Tom Albright (Salk)
Sergei Gepshtein (Salk)
Dan Aharoni (UCLA)
Valarie Williams (OSU)
...

Funding
NSF GRFP
BRAIN EAGER/R01 (MM+JS)
Princeton IP Accelerator Award
Princeton Porter Ogden Jacobus Fellowship
NSF Award #2217975 (w/ Tom Albright)

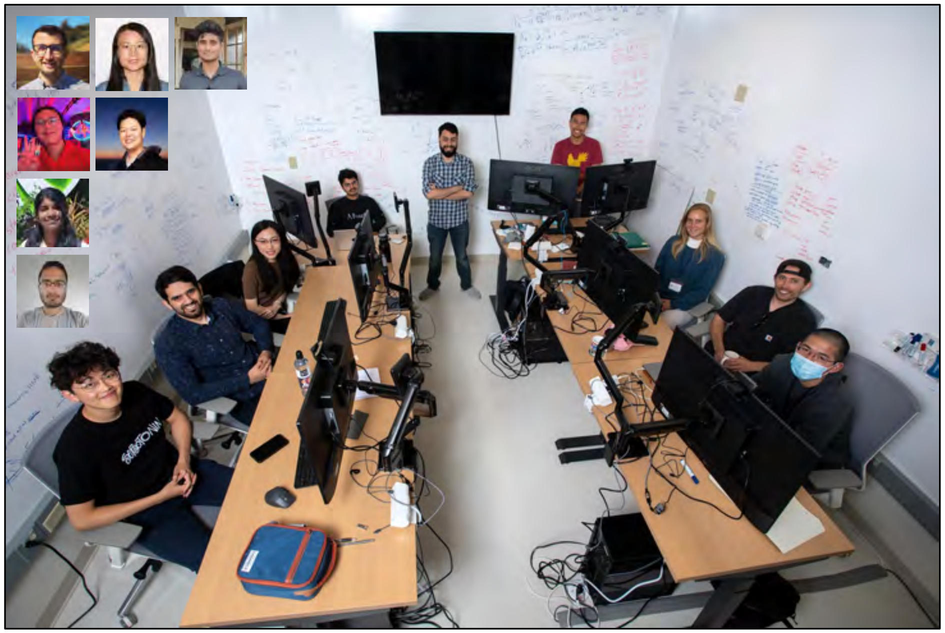

Get some SLEAP at sleap.ai!

Talmo Lab
Advaith Ravishankar
Aaditiya Prasad (w/ Uri Manor)
Divya Murali
Eric Leonardis (w/ Tom Albright)
Liezl Maree
Lin Wang (w/ Wolfgang Busch)
Nathanial Nono (w/ Azim Eiman)
Scott Yang
Shrivaths Shyam
Vincent Tu

Alumni:
Pranav Sankar (now at UCLA)
Chaitanya Kapoor
Dexter Tsin (now at Princeton)
Nancy Guo (now at NYU)
Adam Lee (now at Harvard)
Arlo Sheridan (now at E11 Bio)
Sean Afshar (now at Princeton)
David Samy (still in high school!)
Theo Couris (now at TCU)
Will Knickrehm (still in high school!)
Zaher Abbara (now at Cal Poly SLO)

Learn more @ talmolab.org!

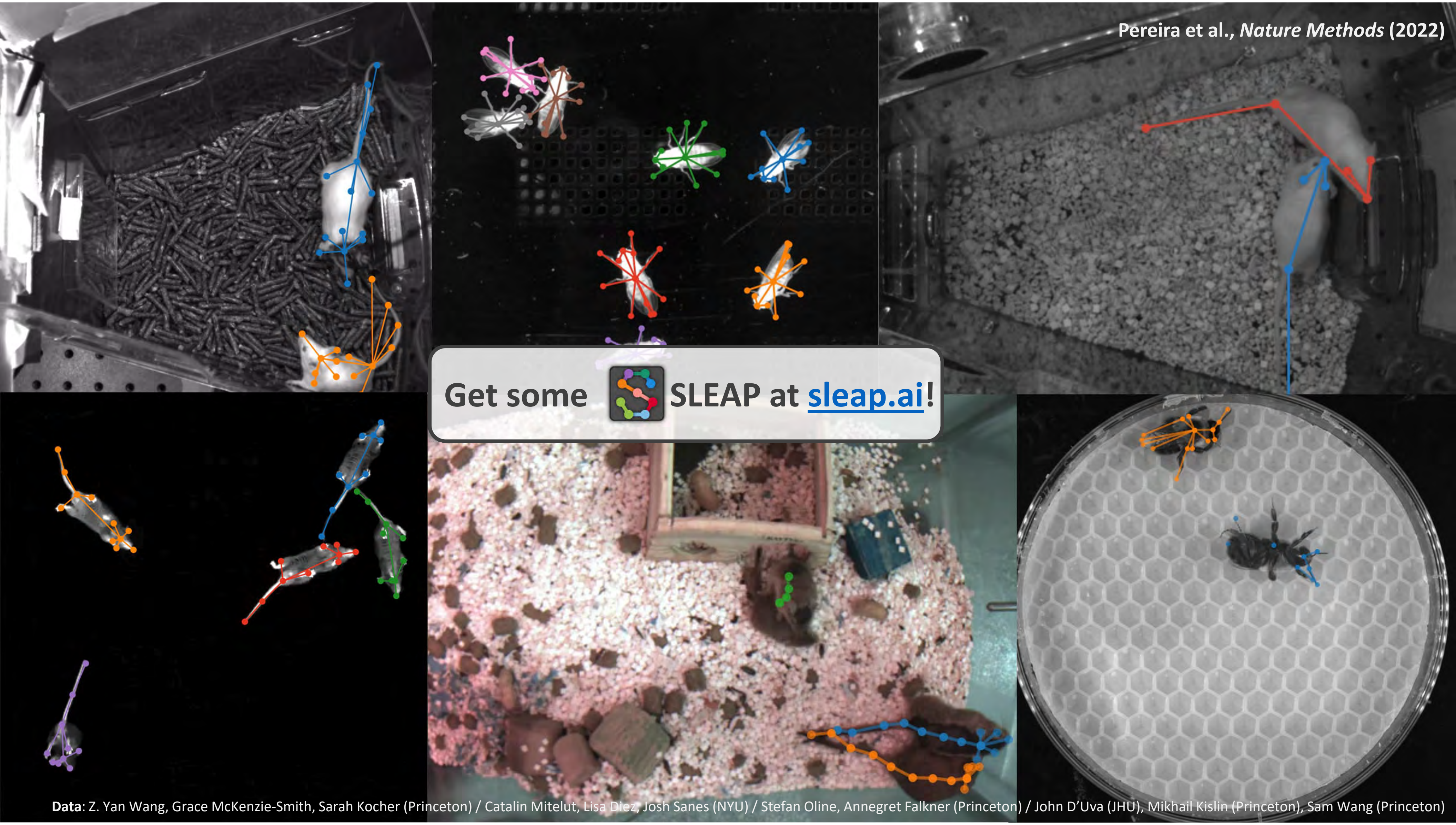
Pereira et al., *Nature Methods* (2022)
Get some SLEAP at sleap.ai!
Data: Z. Yan Wang, Grace McKenzie-Smith, Sarah Kocher (Princeton) / Catalin Mitelut, Lisa Diez, Josh Sanes (NYU) / Stefan Oline, Annegret Falkner (Princeton) / John D'Uva (JHU), Mikhail Kislin (Princeton), Sam Wang (Princeton)

SLEAP: An industry-grade AI system

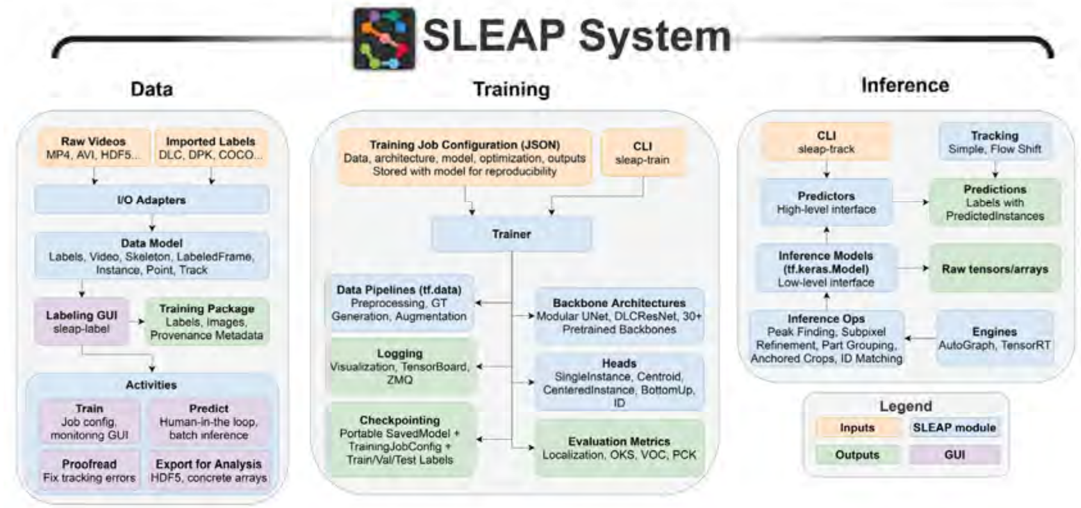

No code GUI workflow

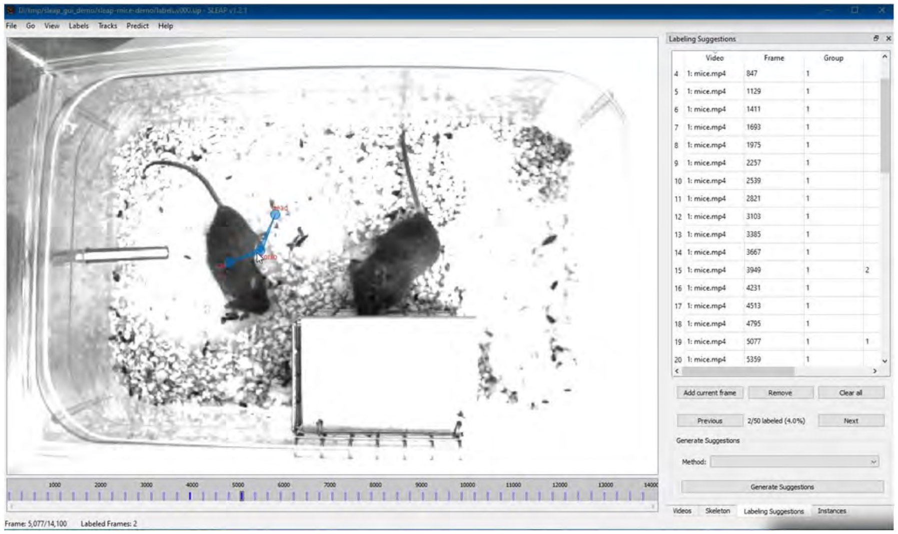

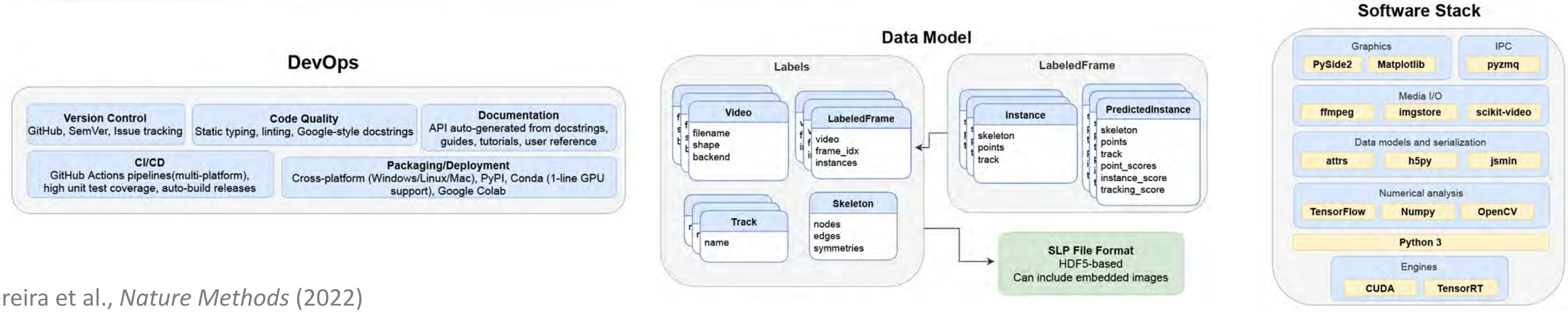

Pereira et al., *Nature Methods* (2022)

Localization + part grouping + ID assignment

SLEAP: End-to-end speed

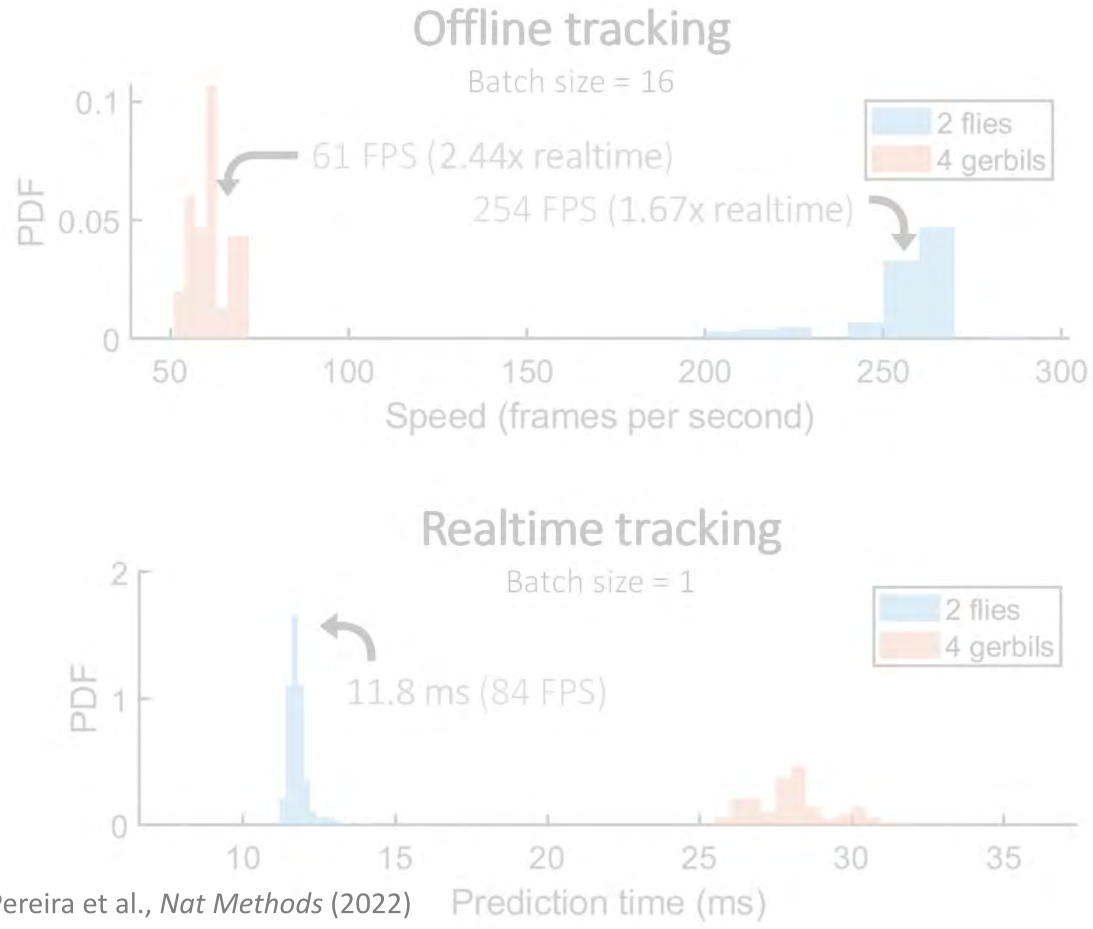

Pereira et al., *Nat Methods* (2022)

Offline | Realtime

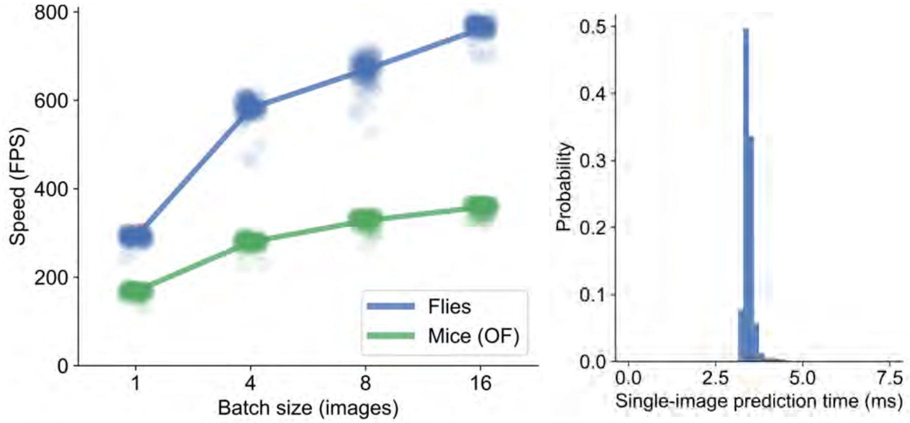

254 FPS → 762 FPS (3x faster)

11.8 ms → 3.5 ms (3.4x faster)

w/ Kaleb Smith (NVIDIA)

Localization + part grouping + ID assignment

SLEAP: End-to-end speed

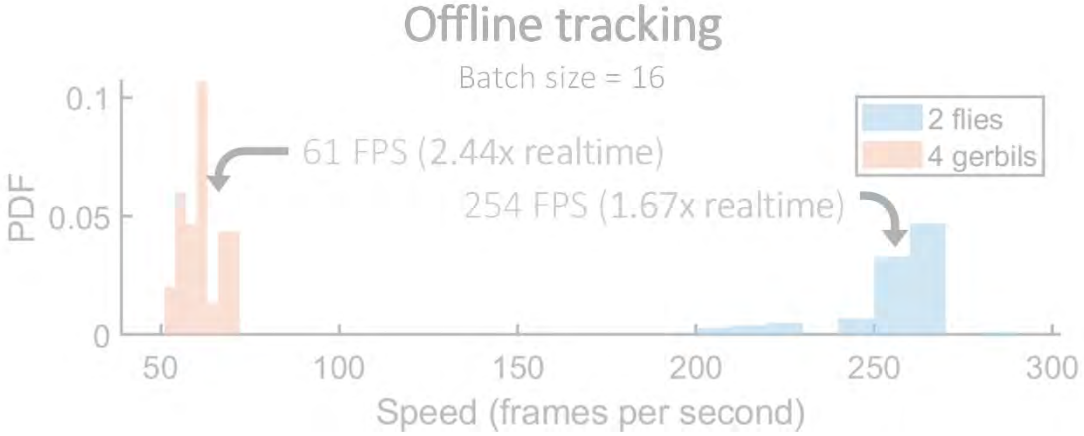

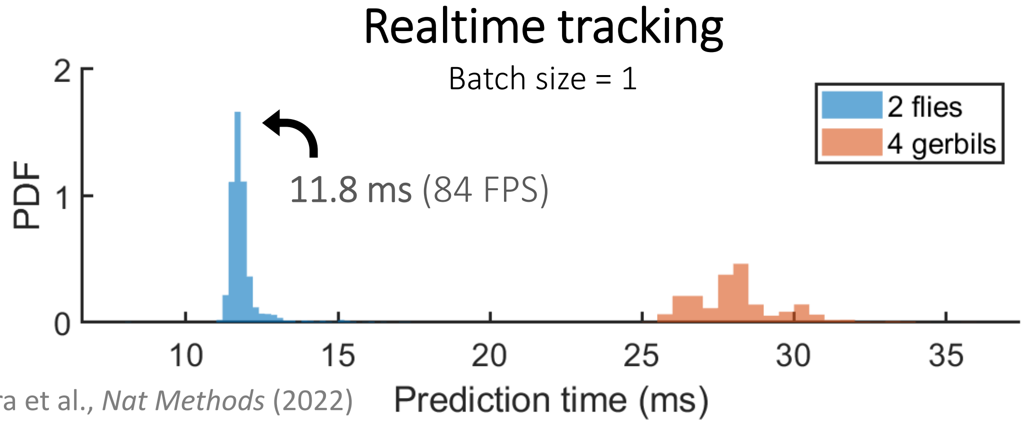

Pereira et al., *Nat Methods* (2022)

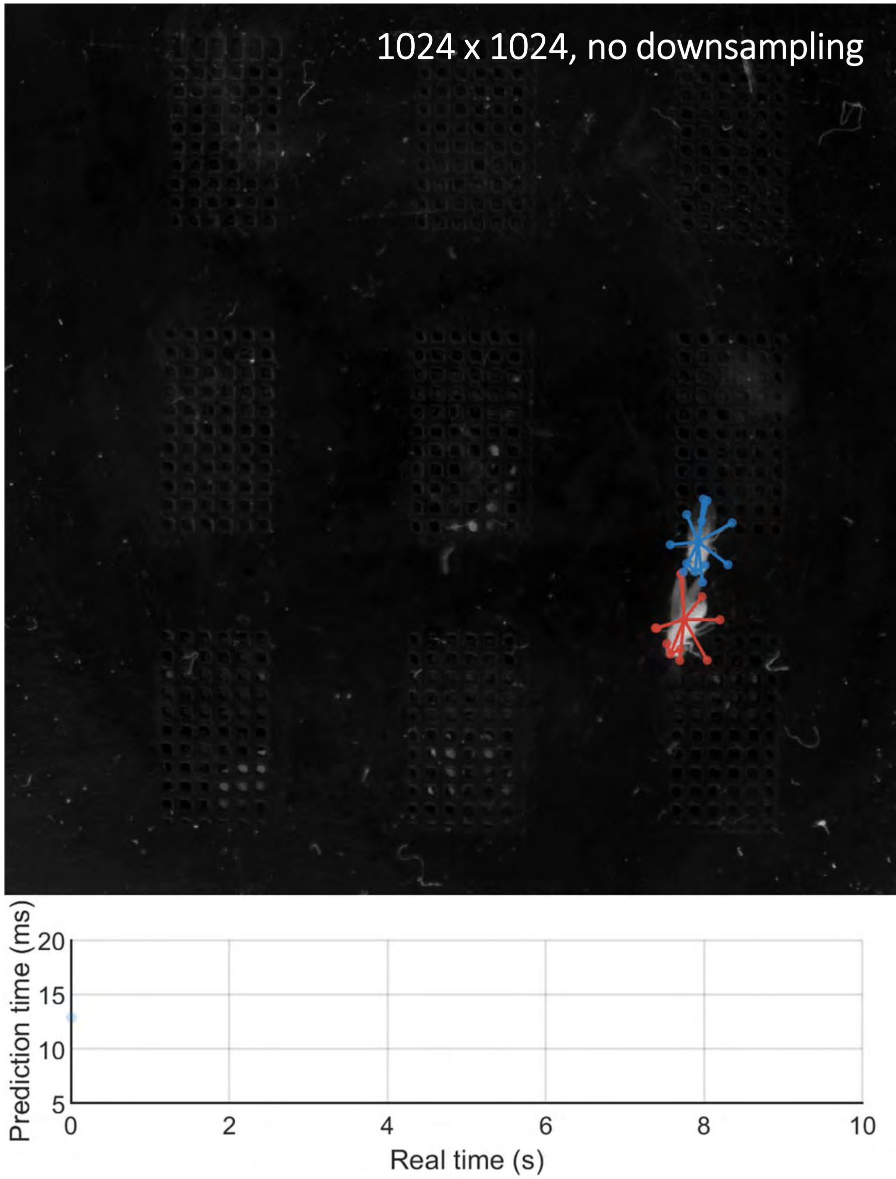

Overview of the domains/dimensions impacted by pain

B. Duncan X. Lascelles
BSc, BVSC, PhD, FRCVS
CertVA, DSAS(ST), Diplomate ECVS, Diplomate ACVS

Dr. J. McNeely and Lynne K. DuBose Distinguished Professor of Musculoskeletal Health
Alumni Association Distinguished Graduate Professor
Professor of Translational Pain Research and Surgery
Director, Translational Research in Pain [TRiP] Program
Director, Comparative Pain Research and Education Center
North Carolina State University College of Veterinary Medicine, Raleigh, NC, 27606, USA

The backbone of pain assessment in humans is self-report. In non-verbal humans and in animals, self-report is not an option, and thus one might consider the measurement of pain to be an uphill struggle. Indeed, without the option of self-report of pain, the measurement of pain in any species is difficult, but on the positive side, pain affects individuals in a multi-dimensional manner. It has both neurophysiologic and affective components and this means that a variety of aspects (or domains) of feeling, behavior and function are affected. This multi-dimensional impact in people includes cognitive, affective, behavioral, functional, physiological, sensory, and socio-cultural dimensions or domains. The multi-dimensionality of pain offers a multitude of opportunities to measure the impact of pain, and hence estimate the burden of 'pain' itself.

There is not a 'gold standard' definition of the domains impacted by acute or persistent (chronic) pain. The domains impacted by pain which are described and used in studies vary with the pain condition and the context in which they are being discussed. There is further variation depending on whether domains that contribute to quality of life / health are being described (in reference to the impact pain has on health), or whether the focus is on what aspects pain impacts. Further, the descriptions of domains impacted by pain can be viewed from the perspective of what measurement tools are available.

Regardless of the way in which one describes 'domains' in relation to pain, the fact is that pain has widespread and varied impacts across all aspects of an animal's life. This provides opportunity for the development of multiple outcome measures, and by extension, provides the opportunity to gain a more holistic view of the negative impact of pain, or the utility of an analgesic intervention.

A starting point for consideration of the broad multi-dimensional impact of pain might be to consider the following domains:

- Movement and mobility
- Ability to perform the activities of daily living
- Cognitive function
- Affective states (fear, anxiety)
- Interactions with animals and humans
- Physiological function
- Sensory processing
- Sleep

Such a list can be used to explore varied and new ways to approach measurement of the impact of pain. For example, in canine osteoarthritis, there has been an emphasis on the measurement of limb use and

function, but assessing other domains such as cognitive function, and sleep quality may provide meaningful insight into the impact of pain.

When one considers the domains that are impacted by pain, it is important to understand that within each domain are many varying aspects that can be affected – and so opportunities for measurement. For example, canine osteoarthritis pain clearly impacts the domain of 'movement and mobility'. But within this, it is understood that there are multiple components that can be impacted by joint pain:

- Limb use
- Overall activity
- Smoothness or quality of motion
- Power of movement
- Resting body weight distribution
- Speed of motion
- Willingness to move

Even within each of these are multiple measures – for example with 'limb use' one can measure peak vertical force, vertical impulse, propulsion and braking forces.

A comprehensive understanding of the domains and their components that are impacted by different pain states and conditions will lead to the development of new measurement approaches and tools. A challenge will be to understand the varying ways in which pain impacts different individuals within a pain condition, and therefore the importance of measuring one domain, or one aspect of a particular domain, in both individual and groups of animals.

The Biospychosocial Model of Pain

Prof Mark R Hutchinson, PhD(Med), BSc(Hons)
University of Adelaide

Within veterinary science, pain management, and animal behaviour, the multidimensional nature of pain is a focal area demanding a deep and nuanced understanding. This presentation ambitiously embarks on an exploration of pain that spans across an array of scales: from intricate nanoscale molecular interactions to macroscopic cellular responses, and further, to systems responses. These vast complexities, occurring across disparate time frames – from ephemeral millisecond reactions to lifelong environmental exposures – find their most coherent synthesis in the biopsychosocial model of pain.

Pain extends beyond its superficial perception as a mere sensation. It embarks on a labyrinthine journey navigating a multitude of internal and external factors, culminating into the poignant human metaphor of a "cancer of the soul." A cornerstone of this discourse will be the symbiotic relationship between immunology and neuroscience. Within this context, the sophisticated interplay of the peripheral and central immune-like systems emerges as pivotal in shaping both brain functions and resulting behavioural outcomes. This paints a vivid tableau where biopsychosocial constructs entwine with the molecular and cellular facets driving these connections.

Evolving discourse around the biopsychosocial model accentuates the role of the neuroimmune brain, emphasizing the immune resilience of the Central Nervous System (CNS). This system, gaining prominence as "the other brain," stands sentinel, orchestrating a harmonious integration of multifarious life experiences. Translated into neurokine signals, these experiences stimulate neuroimmune cells in the CNS to adapt and evolve, redefining the context in which neurons operate. Notably, when these shifts localize within the somatosensory neuroanatomical domains, phenomena such as hypernociception and persistent pain become manifest.

Delving into the annals of scientific exploration reveals that our early understanding of neuroimmune signalling, with its profound implications for brain health and pathology, was largely shaped by insights from the illness response. Over the years, revelations have surfaced illustrating how these unique brain-immune processes not only play roles in conventional responses but also influence the pharmacodynamic properties of analgesic agents.

Through this presentation, a tapestry of the latest scientific revelations will be unfurled, highlighting the intricate nexus between the brain's neuroimmune intricacies, enduring pain experiences, and pharmacological responses. Armed with this holistic insight into the biopsychosocial model, attendees will be poised to engage more effectively in innovative pain management strategies and advanced behavioural research.

Title: “Automated and remote pain delivery with automated pain measurement”

Ishmail Abdus-Saboor

Associate Professor & Principal Investigator, Columbia University’s Zuckerman Mind Brain Behavior Institute

Abstract:

Describing one’s internal pain state can be extremely challenging to communicate verbally. This challenge is compounded in nonverbal rodents that cannot articulate their sensory experiences to onlooking experimenters. Distinguishing between the sensory and emotional states of pain in animals has been extremely challenging because traditional measurements of pain, such as latency or frequency to withdrawal to a sensory stimulus delivered to the paw, provide limited resolution. The Abdus-Saboor lab uses fast video imaging, computation, and mathematics in an attempt overcome this problem and provide more resolution. An innovative aspect of our work is recording sub-second behaviors in freely behaving rodents and analyzing the data with artificial intelligence to measure pain to create rodent “pain scales.” We define pain sensation by rapid paw withdrawal, and pain “affect” with coping and defensive behaviors including intense paw shaking or guarding, jumping, or facial grimace. This automated technology, that we named PAWS (Pain Assessment at Withdrawal Speeds) allowed us to measure pain sensation versus pain “affect” in the mouse in an unbiased manner. In more recent collaborative work we used PAWS to uncover affective pain features in a variety of chronic pain models. Additionally, we used unsupervised machine learning to uncover spontaneous displays of pain and pain relief.

In this presentation, I will also describe a new device we have created. Sensory-reflexive testing of mechanical threshold in rodents is one of the most reliably used approaches to approximate the pain state in pre-clinical rodent models. We have built a device that can automatically and remotely deliver either innocuous or noxious mechanical stimuli to behaving, yet semi-constrained mice. We couple this device to the PAWS platform described above. Together, these approaches offer a way to increase the rigor and reproducibility in which mechanical nociceptive assays are performed and interpreted in mice.

Pain is an unpleasant sensory and emotional experience associated with, or resembling that associated with potential tissue damage. Pain is a multidimensional experience that impacts the person with pain across multiple domains across the biopsychosocial spectrum. Chronic pain can occur from activation of nociceptors in peripheral tissues (nociceptive pain), from damage to the somatosensory system (neuropathic pain), and from altered activity in the central nervous system (nociplastic). Furthermore, pain impacts and can be impacted by psychosocial factors and the motor system. Further, certain factors within the biopsychosocial spectrum can predict the transition to chronic pain and recover and response to treatment. Therefore, assessment of the individual with pain encompasses not only the pain, but also the impact of pain on the person. Assessment of pain can be divided into the following domains: Pain, co-morbid symptoms, psychosocial factors, function and disability. Most assessments of pain and its symptoms are done through self-report. Importantly for pain, the gold standard is self-report. Pain is often assessed at rest, during movement, and the area of pain using verbal rating scales and body maps. Further the mechanistic type of pain, nociceptive, neuropathic or nociplastic can be assessed rough self-report measures. Co-morbid symptoms include cognitive and physical fatigue, sleep, and cognitive function. Psychosocial factors are through self-report and include depression, anxiety, fear of movement, pain catastrophizing and pain self-efficacy. Physical function is measured through self-report, performance based tasks, activity monitoring. Additional constructs of pain interference and pain disability aim to determine the impact of pain on a variety of functional activities including home responsibilities, social activity, work, self-care and recreational activities. This talk with highlight the different types of assessment commonly performed in individual with chronic pain and how these constructs are currently assessed in animal models of pain.

Understanding what is considered a within-patient clinically-meaningful change in the outcome measures used to assess pain can improve interpretation of clinical trial results. This presentation will address the various methods that can be used to estimate within-patient, clinically-meaningful changes in outcomes, including-anchor-based methods, a "stop-watch" method for acute pain, qualitative interviewing, and discrete choice methodologies. Important considerations for interpreting clinically meaningful differences, including variation between people and contextual factors will also be addressed. Differences between within-patient and between group differences will be highlighted, specifically the importance of not applying within-patient minimally important differences to interpreting between group differences. Finally, points to consider when planning whether and how to apply similar concepts to animal research will be highlighted for discussion.

Minimal clinically-important difference for 'Liverpool Osteoarthritis in Dogs' (LOAD) and Canine Orthopedic Index (COI)

John F. Innes[1], Mark Morton[1], B. Duncan X. Lascelles[2], Joao Alves[3]

1 – Movement Referrals: Independent Veterinary Specialists, 3 Abbots Park, Preston Brook, Runcorn WA7 3GH, UK
2 – College of Veterinary Medicine, North Carolina State University, Raleigh, NC 27607, USA
3 - Divisão de Medicina Veterinária, Guarda Nacional Republicana (GNR). Rua Presidente Arriaga, 9 1200-771 Lisbon, Portugal.

Several client-reported outcomes measures (CROMs) have been validated for canine orthopaedics . A COSMIN-based review concluded that three CROMs [Liverpool Osteoarthritis in Dogs (LOAD), Canine Orthopedic Index (COI) and the Canine Brief Pain Inventory (CBPI)] can be recommended for use in dogs with osteoarthritis. [1] However, the minimal clinically-important difference (MCID) has not been reported for these CROMs. MCID is the smallest change in an outcome that a client would identify as important. MCID can be estimated using anchor-based methods or distribution-based methods. In these studies, we set out to estimate MCID for LOAD and COI using data from two populations: RCVS Knowledge Canine Cruciate Registry (CCR) and a cohort of dogs with hip osteoarthritis at the Portuguese Gendarmerie Canine Clinic.

RCVS Canine Cruciate Registry Population

Pre-surgery and 6 weeks post-operative data from the CCR were used. The anchor question was: "Compared to before surgery, how is your dog now?" and possible responses were, "much better", "somewhat better", "the same", "somewhat worse" and "much worse". Data from the CCR were exported in July 2022. The Mann-Whitney U test was used to compare differences in the CROMs and the mean change in the CROMs between the "the same" and "somewhat better" groups from the anchor question. Four anchor-based and two distribution methods were used to estimate MCID.

Data from 133 subjects were available. MCID estimates are shown in Table 1.

Table 1: MCIDs for LOAD and COI

Anchor based					Distribution based	
**CROM**	AC	Change difference	MDC	ROC Curve (AUC)	Effect size	SEM
LOAD	-7.4	-8.8	-3.6	-1.0 (0.867)	±1.5	±2.4
COI	-17.6	-13.9	-14.3	-3.5 (0.785)	±2.2	n/a

AC, average change; MDC, minimal detectable change; ROC, receiver operator characteristic; AUC, area under ROC curve; SEM, standard error of measurement

Portuguese Gendarmerie Canine Clinic population

Baseline and 30 post-treatment follow-up data from 296 dogs treated for hip osteoarthritis were categorized based on an anchor question, and estimates of MCIDs using distribution-based and anchor-based methods were performed.

For the LOAD, the anchor-based methods provided a range of -2.5 to -9.1 and the distribution-based methods from 1.6 to 4.2. For the COI, the anchor-based methods provided a range of -4.5 to -16.6 and the distribution-based methods from 2.3 to 2.4. For the dimensions of COI, values varied from -0.5 to -4.9 with the anchor-based methods and from 0.6 to 2.7 with the distribution-based methods. Receiver operator characteristic curves provided areas under the curve >0.7 for the COI, indicating an acceptable cut-off point, and >0.8 for the LOAD, indicating an excellent cut-off point.

Our estimates of MCIDs for dogs with hip OA were consistent with previously proposed values of -4 for the LOAD and -14 for the COI as derived from the RCVS Canine Cruciate Registry data. We also presented estimates of -4 for 'Stiffness', 'Function', and 'Gait' and -3 for 'quality of life'. These estimates can be used for research and patient monitoring.

In summary, the authors suggest a 'working' MCID for LOAD of 4 and 14 for COI. Further estimates are required in different clinical contexts.

Acknowledgements

Elanco Animal Health for the use of LOAD; RCVS Knowledge for access the CCR data.

References

1. Radke H, Joeris A, Chen M: Evidence-based evaluation of owner-reported outcome measures for canine orthopedic care - a COSMIN evaluation of 6 instruments. Veterinary Surgery 51:244-253, 2022.

Discussion of Application of Success-Failure to Pain Outcome Measures: The Canine Brief Pain Inventory

Dottie Cimino Brown MS, DVM, DACVS
Vice President Science & Healthcare Innovation
Mars Veterinary Health

The Canine Brief Pain Inventory:

The Canine Brief Pain Inventory (CBPI) is a publicly available owner-completed questionnaire designed to quantify the severity of chronic pain and its impact on routine activities in companion dogs. The instrument includes four questions pertaining to the severity of pain that are averaged to generate the Pain Severity Score (PSS) and six questions pertaining to the degree to which pain interferes with the dog's routine activities, which are averaged to generate the Pain Interference Score (PIS).

Identifying Success in Individual Animals:

Rather than comparing the overall mean or median differences in scores between groups of animals, it can be important to assess whether the treatment has a measurable effect for individual animals. Particularly in the context of clinical studies for drug development, the criteria for successful treatment of an individual animal are predefined, so that success or failure of the treatment in each animal can be determined at study completion. The number of treatment successes and failures in each group (often animals that receive an active agent vs those administered a placebo) can then be compared. This method has the advantage of minimizing the impact of outliers in response to treatment, particularly when sample sizes are relatively small.

Methods for Determining a Success-Failure Criteria for the CBPI:

The practice of pooling data from two or more independent data sets generated through identical study designs was used. The pooled data included 150 dogs from double-blind (owner and investigator/study staff), randomized, placebo-controlled clinical studies, where carprofen was used as a positive control. All dogs were > 8 kg with a medical history, clinical signs, physical examination findings, and radiographic findings consistent with osteoarthritis. Only dogs with newly diagnosed osteoarthritis or those that had received no previous treatment for osteoarthritis were included. The CBPI was completed by the same owner for each dog at screening (Day-14 to Day -7), baseline (Day 0), and after two weeks of treatment with placebo or carprofen (Day 14).

The statistical analysis performed on this data set explored the power of defining treatment success as a reduction of 1**,** 2, or 3 in either or both the PSS and PIS, as well as how setting the inclusion criteria at baseline to a PSS and PIS 1, 2, or 3 affected the power of the statistical analysis to detect differences between the placebo and carprofen treatment. The number and percentage of treatment successes and failures were summarized by treatment group. Possible differences between treatment groups were evaluated with the X^2 test. For each definition of success within each population, power was calculated by means of a continuity-corrected 2-sided *z* test, with a= 0.05

Success-Failure Criteria for the CBPI:

Based on the pooled placebo & carprofen data, a study protocol to evaluate treatment effects in dogs with osteoarthritis will be most useful if:

- **the inclusion criteria at baseline (Day 0) are predefined as a PSS and PIS each $\geq$ 2 and**
- **success for each patient is predefined as a decrease $\geq$ 1 in PSS and a decrease $\geq$ 2 in PIS.**

Although this kind of analysis requires more animals to be enrolled in each arm of a study, compared with an evaluation of median change in scores between groups, it allows for determination of response at the individual dog level as opposed to the group level, which may be key to the pivotal evaluation of intervention efficacy.

Validated scales for assessing acute pain in ruminants and pigs: approaches to defining success-failure and what is next?

Stelio P L Luna, DVM, Msci, PhD, DipECVAA

Success in pain assessment is to correctly identify animals suffering pain (true positives) from those that do not suffer pain (true negatives). A “successful” pain scale is the one with the highest sensitivity and specificity. Another attributes to guarantee success in pain assessment are intra (repeatability) and inter-rater reliability (reproducibility). One of the best approaches to assess the methodological quality of studies and to investigate whether an instrument is validated and reliable is the Consensus Based Standards for the Selection of Health Measurement Instrument (COSMIN)[1,2]. According to a recent systematic review using these criteria[3], the only three body behavior-based instruments that scored high for strength of evidence were the Unesp-Botucatu Composite Acute Pain Scale for assessing postoperative pain in cattle (UCAPS)[4], sheep (USAPS)[5] and pigs (UPAPS)[6]. After this review the Unesp-Botucatu Goat Acute Pain Scale (UGAPS) has also been published following COSMIN guidelines[7]. The cut-off point for indication of intervention analgesia of these instruments, based on the Receiver Operating Characteristic curve, increase the accuracy for decision-making on whether or not treating pain, therefore minimizing oligoanalgesia and improving welfare. Because validation is an ongoing process, the instruments required clinical validation and some more work to fill their gaps. The original pig scale had been developed in weaned 38 day pigs. Considering that pain-related behaviors are more apparent in older pigs, a new study clinically validated the pig scale in 5 day piglets[8] with similar results to weaned pigs[6]. The original cattle scale had been validated only in Bos indicus under field conditions. Now it was clinically validated in Bos and indicus and in the hospital environment[9]. Because sheep are used for translational medicine and laparotomy was used to validate USAPS, an ongoing study is evaluating USAPS in orthopedic surgery. Next questions are to investigate if these results are reproducible when used by untrained or lay subjects and validate the scales in different ages, sex, clinical conditions, other types of pain and in-person instead of video analysis. Although these scales are based only on observation and require a short time for assessment (<4 min), the use of algorithms indicated the relevance of different pain behaviors in pigs[10] and sheep[11]; selecting the most relevant pain behaviors could further simplify the pain scales. Finally the future: artificial intelligence detects pain at least with the same accuracy as USAPS in sheep (unpublished data).

1. Mokkink LB et al. 10.1007/s11136-010-9606-8.
2. Mokkink LB et al. 10.1016/j.jclinepi.2010.02.006. PMID: 20494804.
3. Tomacheuski, RM et al https://journals.plos.org/plosone/article?id=10.1371/journal.pone.0280830
4. de Oliveira FA et al. 10.1186/s12917-014-0200-0
5. Silva NEOF et al https://doi.org/10.1371/journal.pone.0239622.
6. Luna SPLet al 10.1371/journal.pone.0233552
7. Fonseca MW et al https://doi.org/10.3390/ani13132136.
8. Robles I et al https://doi.org/10.1371/journal.pone.0284218
9. Tomacheuski RM https://www.mdpi.com/2076-2615/13/3/364
10. Trindade PHE et al https://www.mdpi.com/2076-2615/12/21/2940
11. Trindade PHE et al https://doi.org/10.1016/j.applanim.2023.106002

2023 Pain in Animals Workshop

Success or Failure: Ground Reaction Forces

Mike Conzemius, DVM, PhD, DACVS
https://www.gqvetsurgery.com/
Queen Creek, AZ

Pain is Universal

Disclaimer

This certifies that the views expressed in this presentation are those of the author and do not reflect the official policy of the NINDS, NIH, or FDA.

Disclosure

This certifies that I, Mike Conzemius, have no financial relationship that is relevant to the subject matter of the presentation.

Best practices for measuring and reporting ground reaction forces in dogs.

Conzemius MG, Torres BT, Muir P, Evans R, Krotscheck U, Budsberg S. Best practices for measuring and reporting ground reaction forces in dogs. Vet Surg. 2022 Apr;51(3):385-396. doi: 10.1111/vsu.13772. Epub 2022 Jan 26. PMID: 35083759.

- SOPs, QA, subjects, study design, data normalization, clinical application, etc.

What Ground Reaction Force Data should be used?

- Minimum considered include velocity, acceleration, PVF and VI.
  - Velocity and acceleration ranges for an acceptable trial should be set prior to data collection. Subject heterogeneity influences normalization of this data to body weight and height.

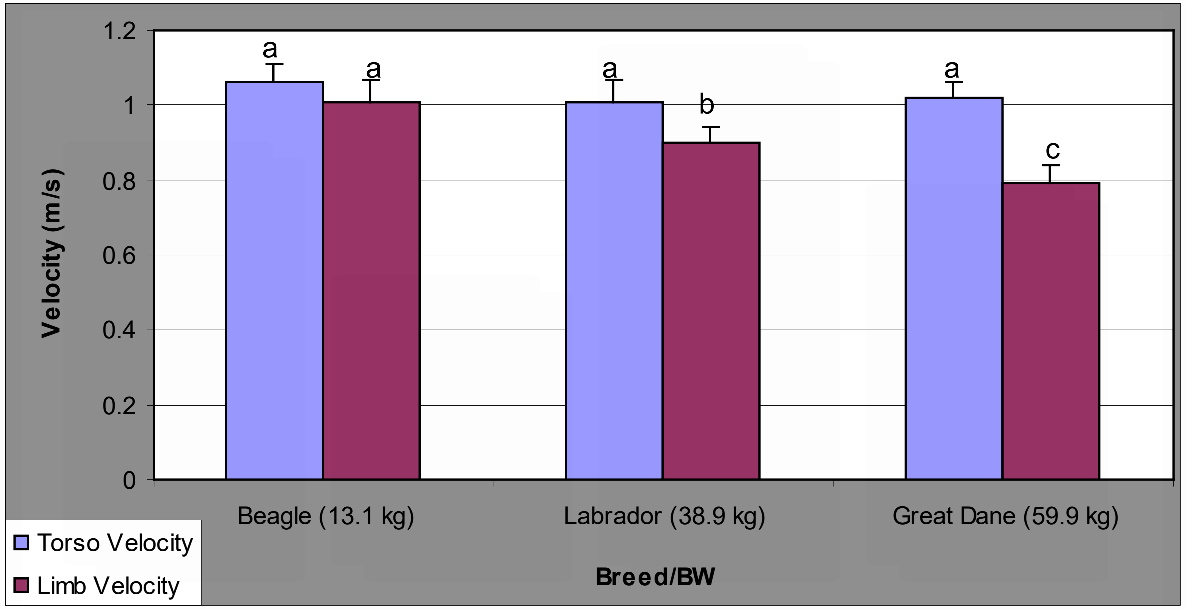

  - Since PVF and VI respond differently to subtle changes in subject velocity, both need to be part of an evaluation. This reduces over interpretation of data. In effect, a change in PVF without context of VI can be irrelevant.

Is a change in PVF from 37 to 40 and VI from 12.5 to 14 in an individual, or group of walking dogs treated for osteoarthritis over 2 months clinically important?

PVF: ((40-37)/37)*100% = 8.1%

VI: ((14.0-12.5)/12.5*100% =12.0%

PVF+VI/2: (8.1+12.0)/2 = **10.05% increase in GRFs**

Is a change in PVF from 37 to 40 and VI from 14 to 12.5 in an individual, or group of walking dogs treated for osteoarthritis over 2 months clinically important?

PVF: ((40-37)/37)*100% = 8.1%
VI: ((12.5-14.0)/12.5*100% = -10.1%
PVF+VI/2: (8.1-10.1)/2 = **-1.0 decrease in GRFs**

Success/Failure in Context of Disease

- Need to have placebo treatment from RCT: best data is for treatment of OA.
- In a canine OA regulatory study placebo data was evaluated over 42 days.
  - Mean data did not change
  - 5%-10% increase in GRFs - 12.1% of dogs
  - 5%-10% decrease in GRFs - 8.6% of dogs
  - >10% change - 1.7% of dogs
  - Thus, GRFs in individual dogs with OA can wax and wane over a short period but a change greater than 5% is unusual and a change greater than 10% is rare.

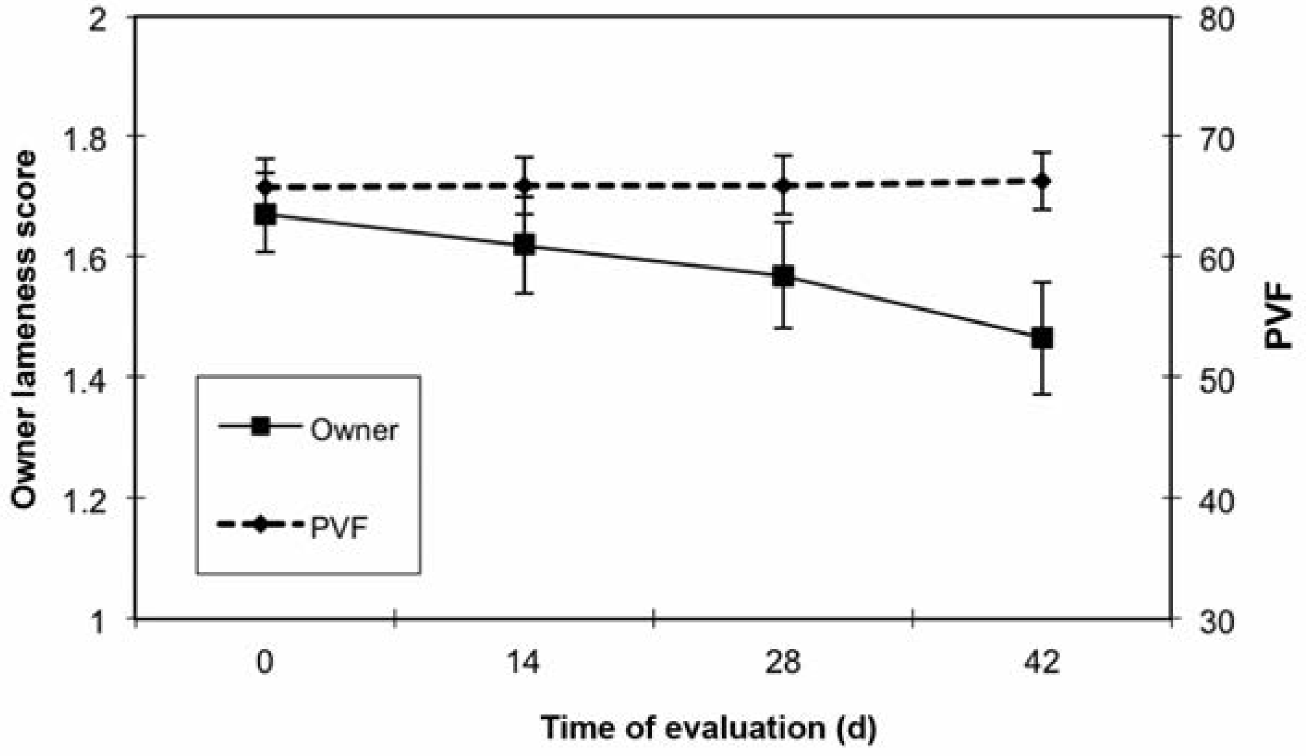

Conzemius MG, Evans RB. Care giver placebo effect in dogs with osteoarthritis. *JAVMA*, 2012, 15;241(10):1314-9. PMID: 23113523

Success/Failure Consensus Statement

- For OA, interventions can be compared to findings RCTs reporting the efficacy of widely accepted treatments.
- <u>Compare Averaged Group Data:</u>
  - Nonsteroidal anti-inflammatory drugs and omega-3 fatty acid diets. The increase in average GRFs over time for these interventions range from 3.5% to 5.6%.
- <u>Address the probability of change in the treatment versus placebo groups:</u>
  - In one RCT study of dogs receiving NSAIDS, 75% of dogs in the treatment group had at least a 5% increase over time. In contrast only 45% of dogs in the placebo group had the same change.

- ***Consensus statement:*** *In dogs with osteoarthritis, a change in both PVF and VI over a short time period (less than 6 months) of 3.5% is the minimum value that should be considered clinically important. The number of dogs in each group that have a change in GRFs greater than 5% is a good outcome measure.*

Authors: James N. Campbell*, James Connolly, Randall Stevens, Jennifer Nezzer

*Presenter

Abstract

There is a widespread sense that inadequacies in trial design and trial conduct detract from the ability to discover safe and effective treatments for chronic pain. These deficiencies may contribute to the striking paucity of new drug innovation over a matter of decades. A critical element of drug development pertains to “assay sensitivity.” Assay sensitivity refers to the ability to detect efficacy when in fact a drug is truly efficacious. Assay sensitivity is affected not only by trial conduct and trial design, but also, the selection of endpoints and the statistical analysis. Optimization of assay sensitivity is relevant to all stages of development of a novel drug therapy, but is most important in Phase 2, where less is known about dose selection and the treatment effects.   In chronic pain drug trials, the typical endpoint is a landmark analysis at 12 weeks. This means that the change in pain over the last seven days of the trial compared to a baseline period for the placebo and test article (TA) determines whether the trial hits the primary endpoint. However, data are typically collected throughout the trial and the 12th week landmark analysis neglects information about the treatment effect by not incorporating the data from the other weeks. Data from just one week could be misleading. Another scenario where a landmark analysis may not be appropriate is where an injection therapy is administered with the expectation that the TA could have a waning effect at week 12 but still be considered effective overall. Each week of data provides information about the difference between the TA and placebo. We posit that if there is no data trend suggesting tachyphylaxis, then the data from all weeks should be incorporated in the analysis. One simple approach to accomplish this is to perform an area under the curve (AUC) analysis based on data from treatment initiation or expected onset of efficacy to the end of the trial. AUC analyses are the norm for acute pain studies but are used inconsistently in chronic pain trials. In this presentation simulations and published trial data will be used to examine the use of AUC analyses and similar alternatives. A case will be made that an AUC approach may be more appropriate than a landmark analysis as the primary endpoint in some circumstances.

Statistical Considerations for Endpoint Choice: Single, Multiple, or Composite Endpoints

Pain studies often utilize more than one outcome to evaluate the effect(s) of a drug or intervention. These outcomes may be evaluated separately as multiple endpoints or combined into a composite endpoint. We briefly review different types of study endpoints (e.g., multiple endpoints, co-primary endpoints, and composite endpoints). There are important clinical and statistical considerations when choosing an appropriate endpoint or endpoints. Statistical considerations include hypothesis testing, balancing Type I and Type II errors, available statistical methods used to analyze different types of endpoints, how potential sources of missing data such as early withdrawals are handled, and interpretability of the results. For example, when multiple endpoints are analyzed in a single clinical trial, there is a risk of increasing the likelihood of making a false conclusion about the effect of a drug on one or more endpoints if the statistical analysis has not been adjusted for multiple testing. We discuss how these considerations may vary depending on the type of study being conducted (exploratory versus confirmatory). We also define the family-wise error rate and false discovery rates and discuss methods for controlling each. We conclude by discussing the importance of pre-specification.

Examples of Use of Multiple or Composite Endpoints in Veterinary Species: Food Animals

Hans Coetzee, BVSc, PhD, DACVCP, DACAW, DECAWBM(AWSEL)
College of Veterinary Medicine
Kansas State University

On July 25, 2017, the U.S. Food and Drug Administration announced the approval of Banamine Transdermal (flunixin transdermal solution), an animal drug approved for the control of pain associated with foot rot and the control of pyrexia (fever) associated with bovine respiratory disease. The label was updated in 2022 to include control of pyrexia associated with acute bovine mastitis with a milk discard time of 48 h. To demonstrate the effectiveness of flunixin transdermal solution for the control of pain associated with foot rot in cattle, two studies were conducted in accordance with Good Clinical Practices (GCP). Each site enrolled 30 Holstein steers approximately 8 months of age that were challenged by subcutaneous injection of a culture of *Fusobacterium necrophorum* into the interdigital space of the right front foot. This trial was characterized by 2 phases. The initial phase involved the development of footrot after disease induction. Calves that developed a lameness score of >3 and a lesion score of 2 or 3 in the front right interdigital space were subsequently randomized to receive either the transdermal flunixin at 3.3 mg/kg or a placebo. Lameness was scored at 6 h after treatment (48 h after lameness induction). Furthermore, a real-time gait analysis system was used to measure maximum total force and contact area on the lame claw at 6 h (+/- 30 minutes) after treatment. A multiple endpoint approach was used with the primary effectiveness variables being the average change in maximum total force and the average change in contact area. In order to demonstrate effectiveness, each study site was required to demonstrate a significant difference in lameness scores and an improvement in the change in contact area and total force on the right front limb at 6 hours after treatment. Composite Endpoints are most commonly used in the development of behavioral scoring systems. Categories of behaviors such as interactions with surrounding and conspecifics, activity, posture, head position or feeding behaviors are typically scored on a scale of 0 -2 or 0-3. Composite endpoints are created using the sum of each component to create a score out of 10. Several composite behavioral scoring systems have been described in veterinary species including cattle and pigs.

Adaptive and Other Innovative Pain Measurement Study Designs

Presenter: Qiao Zhang, M.S., Ph.D., CVM/FDA

Adaptive designs and enrichment designs are increasingly being proposed for use animal clinical trials. CVM published a relevant Guidance for Industry (GFI) 268 “Adaptive and Other Innovative Designs for Effectiveness Studies of New Animal Drugs” in 2021. The guidance discusses the benefits and considerations for a comprehensive list of adaptive and innovative designs. We briefly review two widely used adaptive designs: sample size re-estimation (SSR) and group sequential design, and one enrichment design: placebo lead-in design. These designs provide flexibility and other advantages to conducting the clinical trials, including avoiding underpowering the study, reducing the expected sample size and study duration, and mitigating placebo effect. We discuss the importance of pre-specifying the details of the adaptation or enrichment. Additionally, we discuss the important clinical and statistical considerations when applying these designs in confirmatory studies, including control of the family-wise error rate (FWER), the generalizability of effectiveness results to the target population, the inferential value and independent substantiation of evidence, as well as a reliable evaluation of safety.

Application of AIML to Large Animals for Pain Measurements

Eduarda Bortoluzzi
Assistant Professor of Animal Welfare
Department of Anatomy and Physiology
Kansas State University
bortoluzzi@vet.k-state.edu

Kansas State University
College of Veterinary Medicine

Presenter Notes
2023-08-29 12:07:59

To talk about AIML applications in pain measurement of large animals I first need to introduce a concept of animal welfare. Some of you might be familiar with the framework from Dr. Fraser where he used 3 circles to assess welfare of animals. Basic health and functioning, affective states, and [illegible]

Affective States

"Term that refers to emotions and other feelings that are experienced as pleasant or unpleasant"

"States like pain, distress, and pleasure"

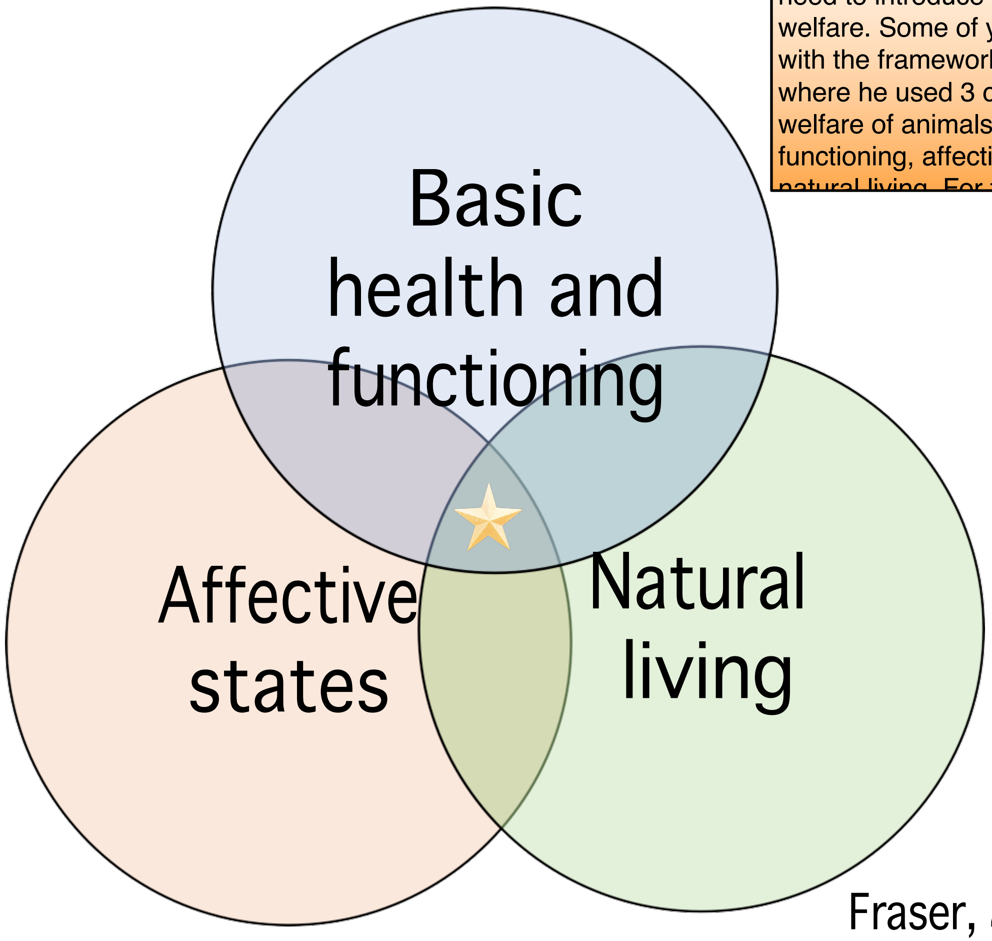

Fraser, 2008

Positive ⟷ Negative

Kansas State University

College of Veterinary Medicine

Affective states

Subjective measures

I am in pain. Do you hear me?

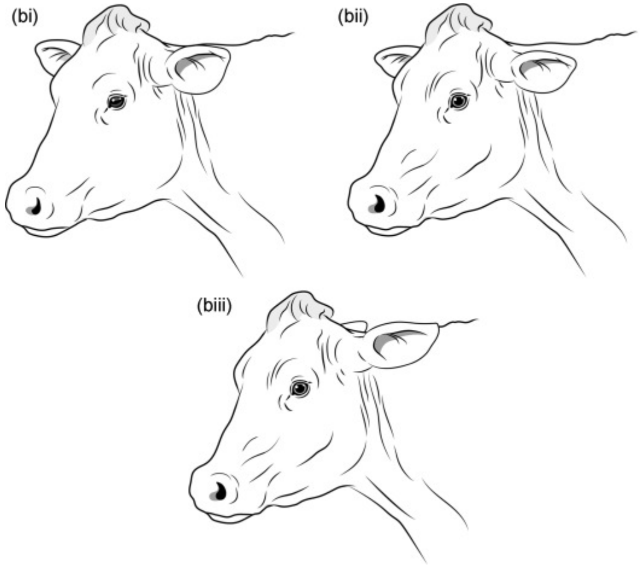

Gleerup et al., 2015

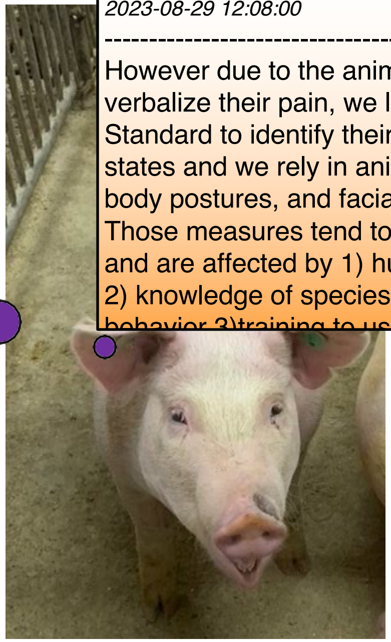

Presenter Notes
2023-08-29 12:08:00

However due to the animal's inability to verbalize their pain, we lack a Gold Standard to identify their affective states and we rely in animal behavior, body postures, and facial grimaces. Those measures tend to be subjective and are affected by 1) human empathy 2) knowledge of species and normal behavior 3)training to use those [illegible]

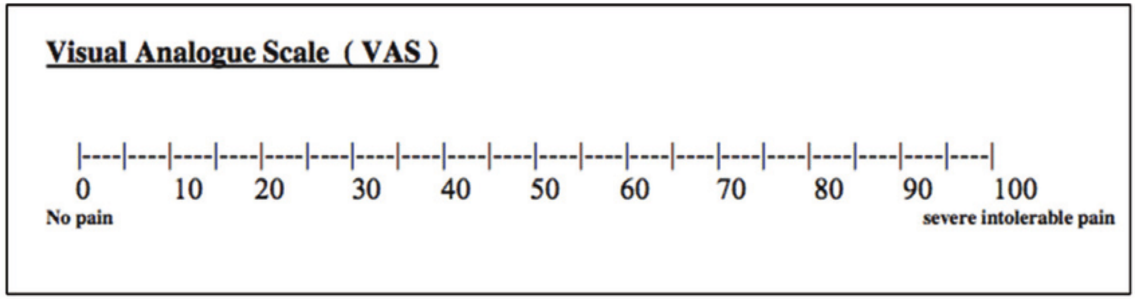

Aggarwal et al., 2018

High variability in pain scores among farmers and veterinarians

Thomsen et al., 2012

KANSAS STATE UNIVERSITY
College of Veterinary Medicine

Behavior observations

- Time consuming
- Require training
- Prone to human error

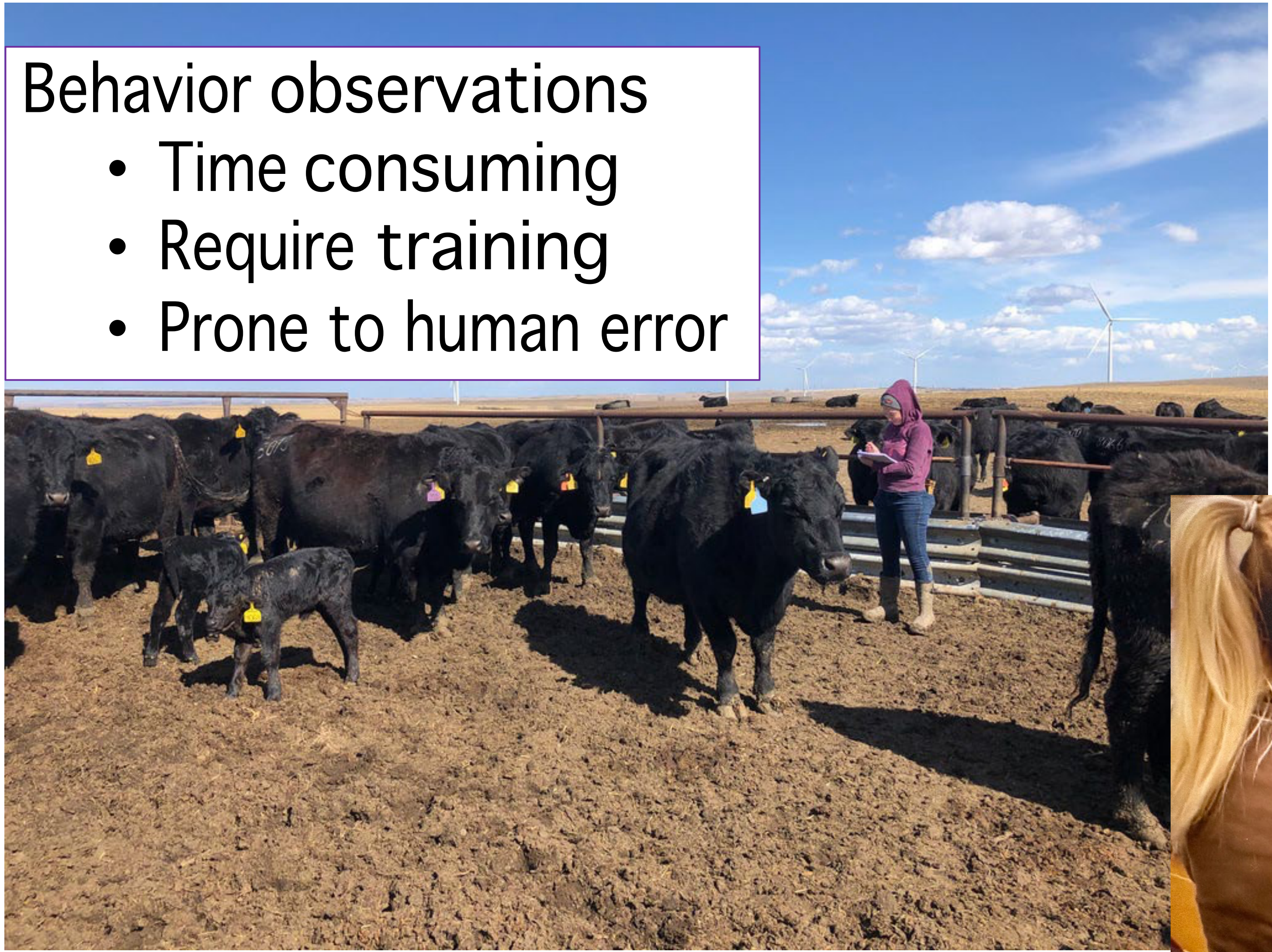

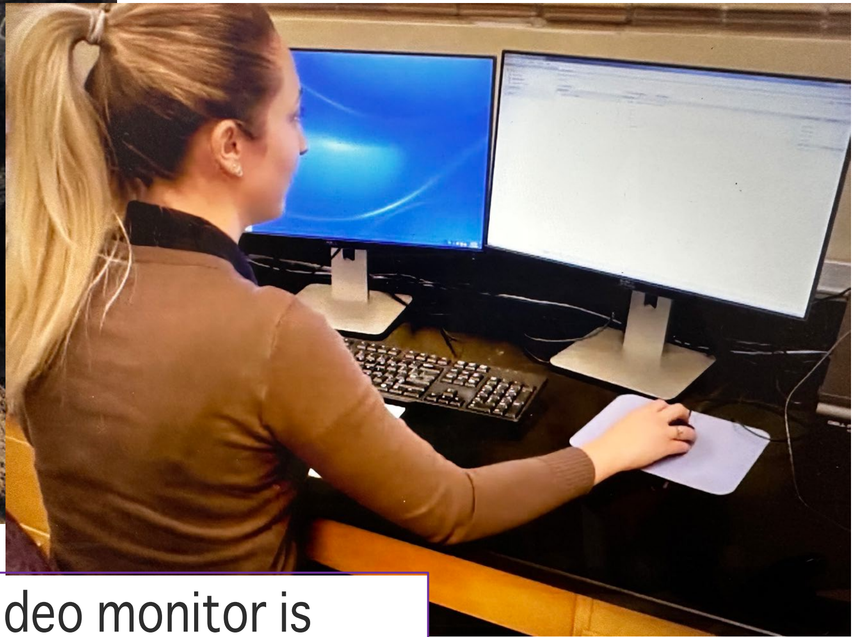

"Spending hours and hours each day sitting in front of a video monitor is mind-numbingly boring, so an observer's attention can easily drift."

Anderson & Perona 2014

KANSAS STATE UNIVERSITY
College of Veterinary Medicine

Presenter Notes
2023-08-29 12:08:01

Human behavior observation are one of our main tools to measure affective states. Nevertheless, they require extensive training, are time-consuming, and are prone to human error. This picture is one of my studies during my Ph.D. where I spent 3 hours inside logging nursing behaviors.

AIML

Subjective measure ⟶ Objective measure

↑ Validity	Quality of the measurement
↑ Realiability	Measurements will repeatedly capture the same information
↓ Bias	Systematic distortion of the data

Kansas State University
College of Veterinary Medicine

G4UM++i•#iMiiW imlll

Presenter Notes
2023-08-29 12:08:01

Human research on facial expressions of pain

Artificial Intelligence and Computer Vision in Low Back Pain: A Systematic Review

by Federico D'Antoni [1], Fabrizio Russo [2,*], Luca Ambrosio [2], Luca Vollero [1], Gianluca Vadala [2], Mario Merone [1,*] R

1 Unit of Computer Systems and Bioinformatics, Universita C 00128 Rome, Italy
2 Department of Orthopaedic Surgery, Universita Campus B Rome, Italy
* Authors to whom correspondence should be addressed.

Semi-automated tracking of pain in critical care patients using artificial intelligence: a retrospective observational study

NaoY-a Kobayashi B, TakuY-a Shig.§, Saori Ikumi, Kazuki Watanabe, Hitoshi Murakami & Masanori Yamauchi

Scientific Reports 11, Article number: 5229 (2021) Cite this article

Feasibility of a Real-Time Clinical Augmented Reality and Artificial Intelligence Framework for Pain Detection and Localization From the Brain

Xiao-Su Hu [1,2]; Thiago D. Nascim
Sean Petty [3]; Stephanie O'Malley:
Eric Maslowski [6]; Alexandre F Da!

Pain Rep. 2022 Nov-Dec; 7(6): e1044.
Published online 2022 Nov 3. doi: 10.1097/PR9.0000000000001044
PMCID: PMC9635040
PMID: 36348668

Artificial intelligence and machine learning in pain research: a data scientometric analysis

Jorn Lotsch,[a,b,*] Alfred Ultsch,[c] Benjamin Mayer,[a] and Dario Kringel[a]

Author information Article notes Copyright and License information PMC Disclaimer

Species	Study	State	Stimulus	Focus area	State Classifier	[illegible]	[illegible]
Sheep	Mahmoud et al. (2018)	Pain	Unkown of naturally occuring	Face	+	Yes	
	Pessanha et al. (2021)		Unkown of naturally occuring	Face	+	Yes	
	Noor et al. (2020)		Unkown of naturally occuring	Face	+	Yes	
Horses	Lencioni et al. (2021)	Pain	Surgical castration	Face	+	Yes	
	Hummel et al. (2020)		Unkown or induced pain	Face	+	Yes	
	Pessanha et al. (2022)		Unkown or induced pain	Face	+	Yes	
	Broomé et al. (2019)		Induced pain	Body and face	+		Yes
	Broomé et al. (2022)		Induced pain	Body and face	+		Yes
	Rashid et al. (2022)		Induced pain	Body	+		Yes
	Reulke et al. (2018)		Vet. Procedure	Body	-		Yes
	Corujo et al. (2021)	Emotion	Unknown	Body and face	+	Yes	
	Li et al. (2021)	-	-	Face	-	-	-
Pigs	Statham	Emotion	Induced behavior	Body	-		Yes

Adapted from Broomé et al. (2023)

Presenter Notes
2023-08-29 12:08:02

Human research on facial expressions of pain

Presenter Notes
2023-08-29 12:08:02

Publication on Plos one. The author used 3000 images to create a dataset for training and testing of a convolutional network to automatically assess horse grimace scale in 7 horses submitted to castration.
his study isbased on assessment offacial expressions of7horses that underwent castration, collected through [illegible]

RESEARCH ARTICLE

Pain assessment in horses using automatic facial expression recognition through deep learning-based modeling

Gabriel Carreira Lencioni[1]*, Rafael Vieira de Sousa[2], Edson José de Souza Sardinha[2], Rodrigo Romero Corrêa[3], Adroaldo José Zanella[1]

1 Department of Preventive Veterinary Medicine and Animal Health of the School of Veterinary Medicine and Animal Science (FMVZ) of the University of São Paulo (USP), São Paulo, SP, Brazil, **2** Department of Biosystems Engineering, Faculty of Animal Science and Food Engineering (FZEA), of the University of São Paulo, Pirassununga, São Paulo, Brazil, **3** Department of Surgery of the School of Veterinary Medicine and Animal Science (FMVZ) of the University of São Paulo (USP), São Paulo, SP, Brazil

* gabriel.lencioni@usp.br

Convolutional Neural Network

↓

Horse Grimace Scale

↓

Castration

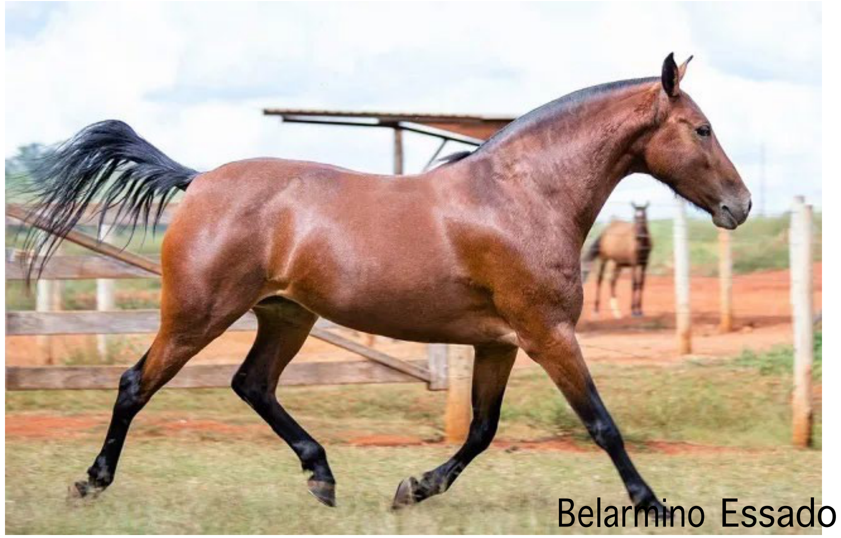

Belarmino Essado

KANSAS STATE UNIVERSITY
College of Veterinary Medicine

Presenter Notes
2023-08-29 12:08:02

Ears = good ability to identify the absence of pain, but it did not obtain the same performance in the differentiation between the Moderately Present and Obviously Present classes.
This result indicates the need for better balancing of the image database with more samples in order to get better

Ears
Accuracy = 90.3%

Eyes
Accuracy = 65.5%

Mouth and nostrils
Accuracy = 74.5%

Co[illegible]

Pain
- Not present
- Moderately present
- Obviously present

75.8%

Pain
- Not present
- Present

88.3%

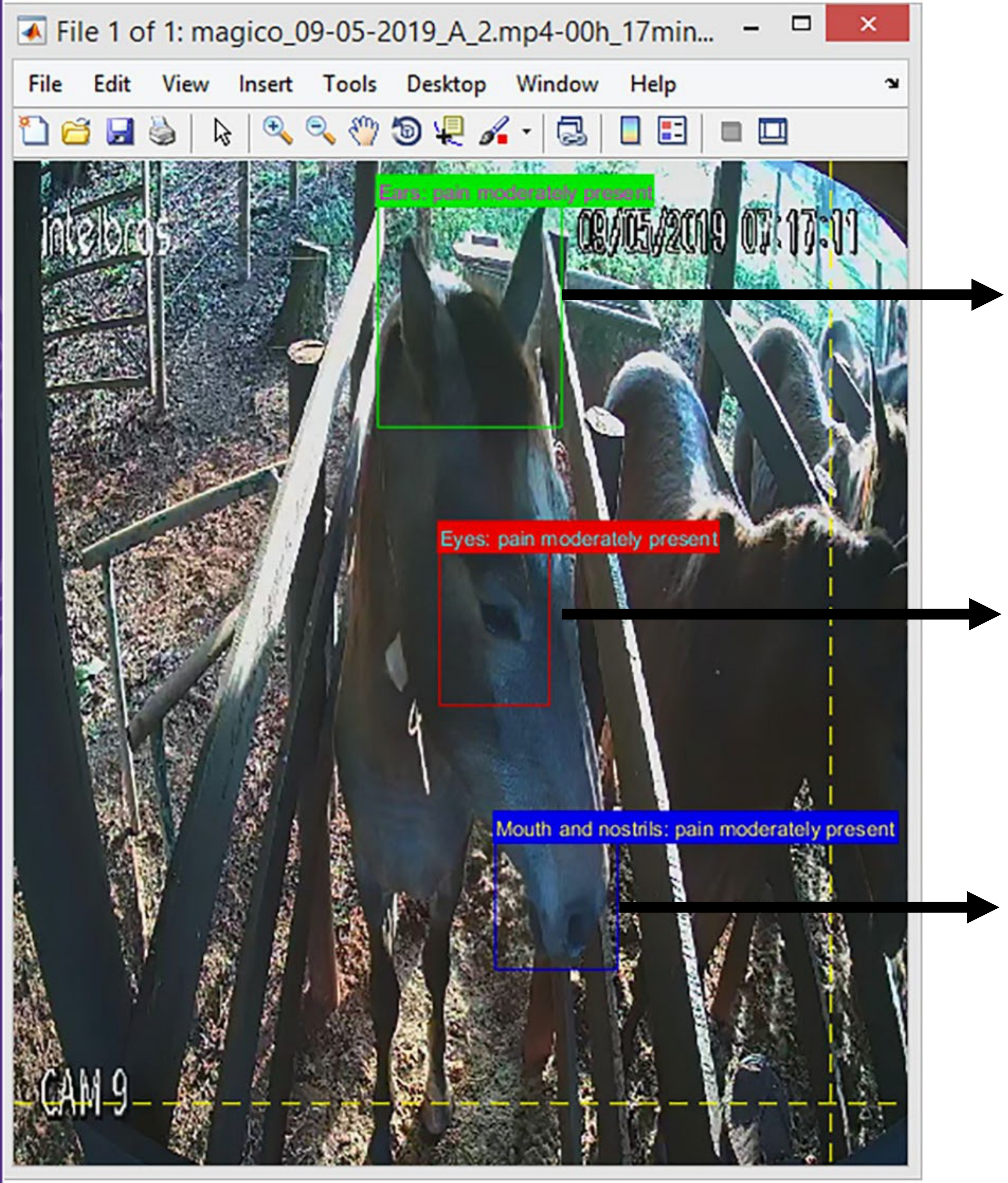

Lencioni et al. (2021)

Kansas State University
College of Veterinary Medicine

2020 15th IEEE International Conference on Automatic Face and Gesture Recognition (FG 2020)

Towards automatic monitoring of disease progression in sheep: A hierarchical model for sheep facial expressions analysis from video

Francisca Pessanha[1,3], Krista McLennan[2] and Marwa Mahmoud[3]
[1] Faculty of Engineering, University of Porto, Portugal
[2] Department of Biological Sciences, University of Chester, United Kingdom
[3] Department of Computer Science and Technology, University of Cambridge, United Kingdom

- Robust sheep face detection model
- Continuous detection and analysis of video
- Accounts for head rotation and self-occlusion
- Temporal nature of facial expressions
- Pain estimation models utilizing regions of interest

KANSAS STATE UNIVERSITY
College of Veterinary Medicine

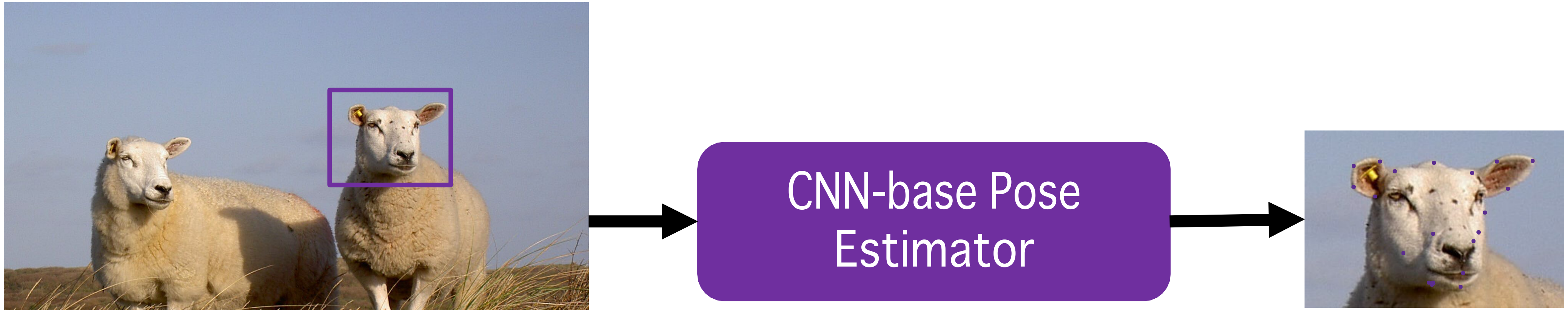

CNN-based face detection

Pose-Informed
Landmark Localization

Adapted from Pessanha et al. (2020)

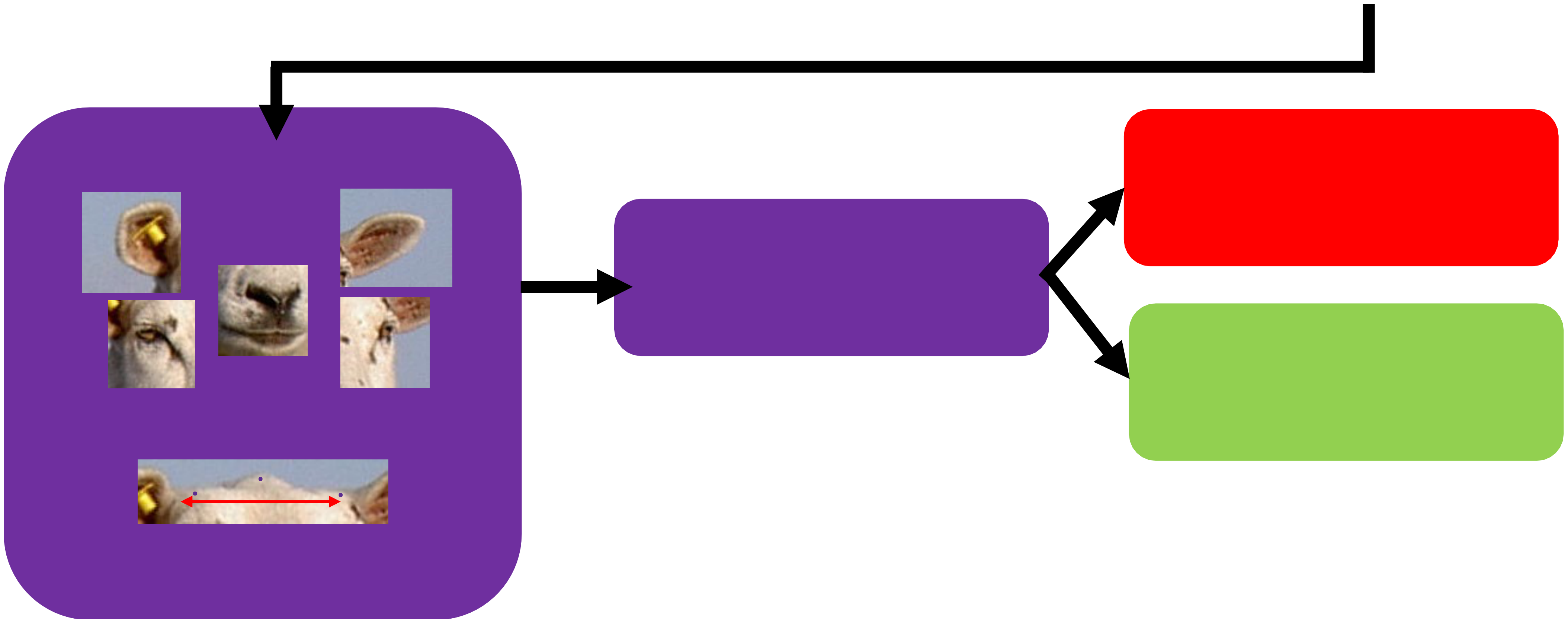

PAIN ESTIMATION RESULTS FQ,R THE SVM MODELS TRAINED WITH THE INDIVIDUAL FEATURES AND THE COMBINED FEATURE VECTOR WHEN APPLIED TO THE DATASET PROPOSED (MAJQ,RITY VOTE: 55 %)

	Pre,cision.	*Recall*	*F1- score*	*Ac,curacy,*
HOGs	0.74	0.66	0.69	0.74
Pos,e	0.76	0.54	0.63	0.7 1
Geometry	0.75	0.49	0.58	10..68
Combined	0.83	0.,68	0.73	10,.78

Open Access 141@1

Evaluation of Precision Livestock Technology and Human Scoring of Nursery Pigs in a Controlled Immune Challenge Experiment

by Eduarda M. Bortoluzzi [1], Mikayla J. Goering [1], Sara J. Ochoa [1], Aaron J. Holliday [2], Jared M. Mumm [1], Catherine E. Nelson [1], Hui Wu [3], Benny E. Mote [2], Eric T. Psota [4], Ty 8. Schmidt [2], Majid Jaberi-Douraki [3,5,6] and Lindsey E. Hulbert [1,*]

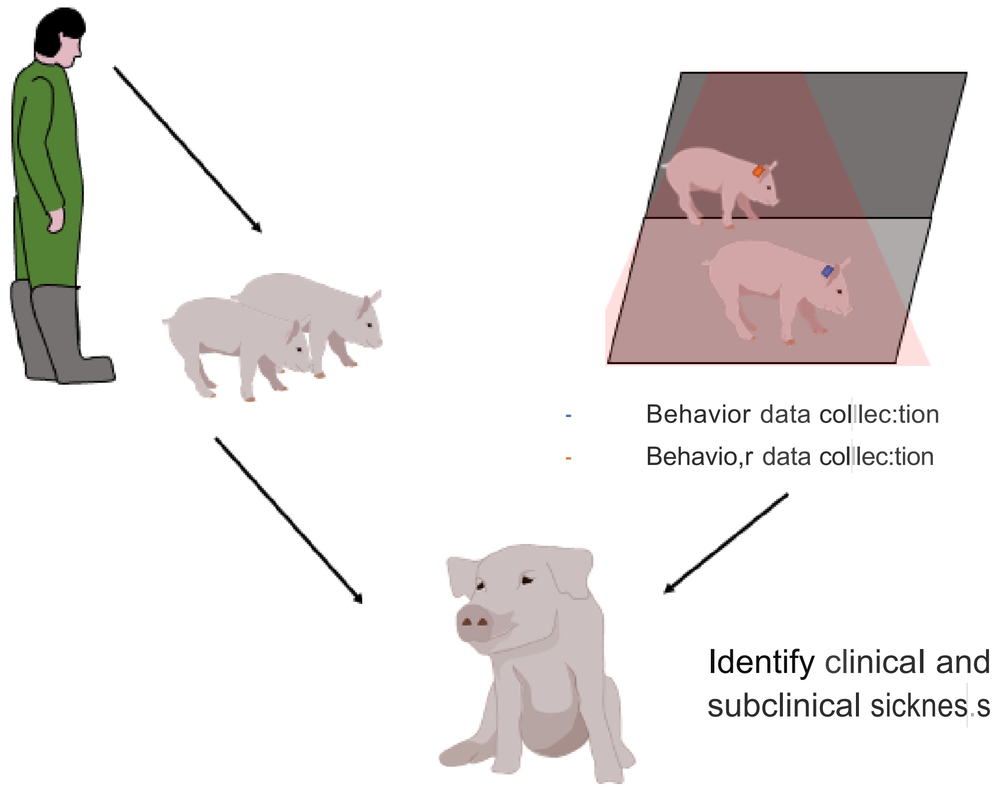

KANSAS STATE UNIVERSITY
College of Veterinary Medicine

Gold standard test

- Challenged = 1
- Sham = 0

Sick Scores

Human observation

NU*track*

Behaviors based on cutoff values

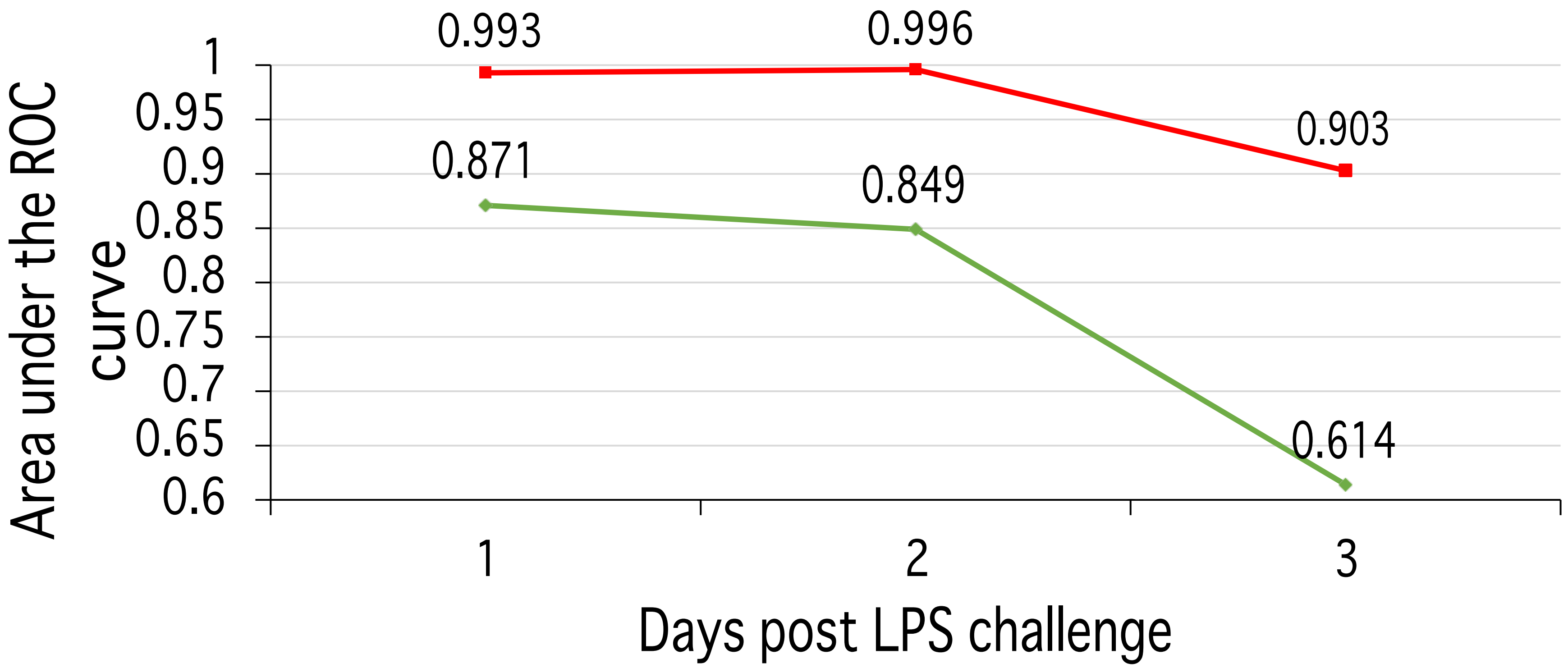

KANSAS STATE UNIVERSITY
College of Veterinary Medicine

Synthetic Data

Synthetic data generation for deep learning model training to understand livestock behavior

by

Armin Maraghehmoghaddam

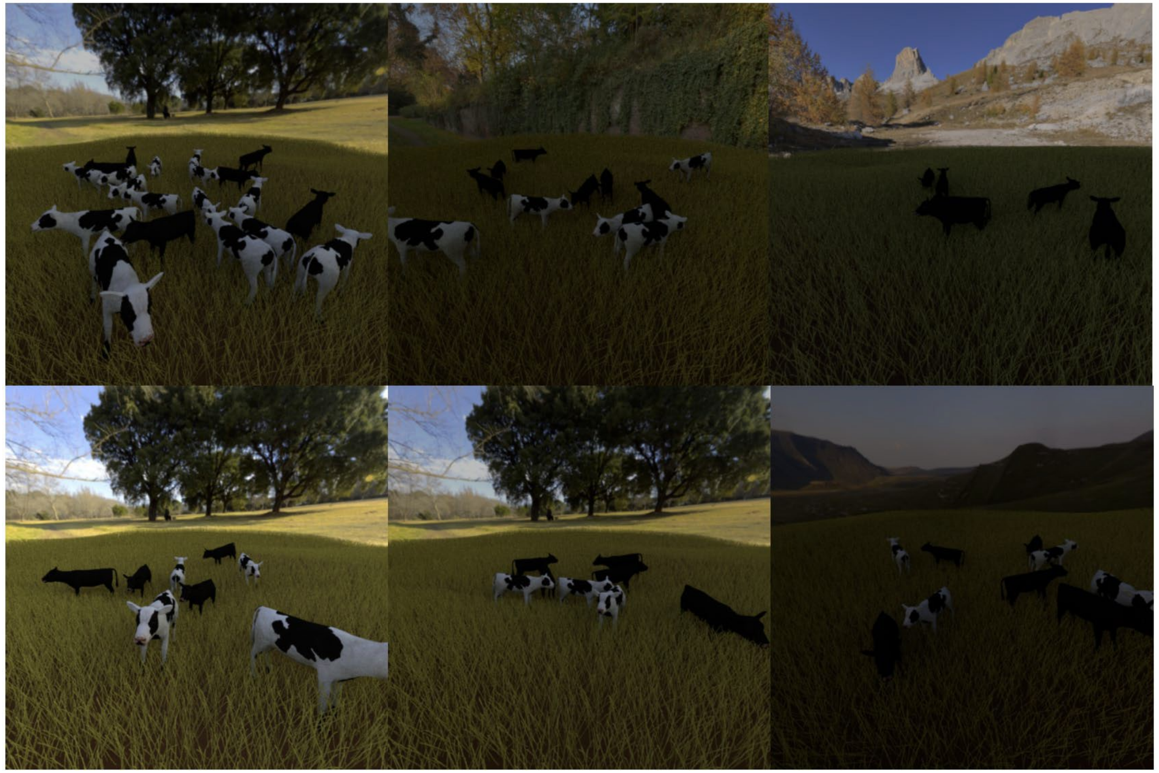

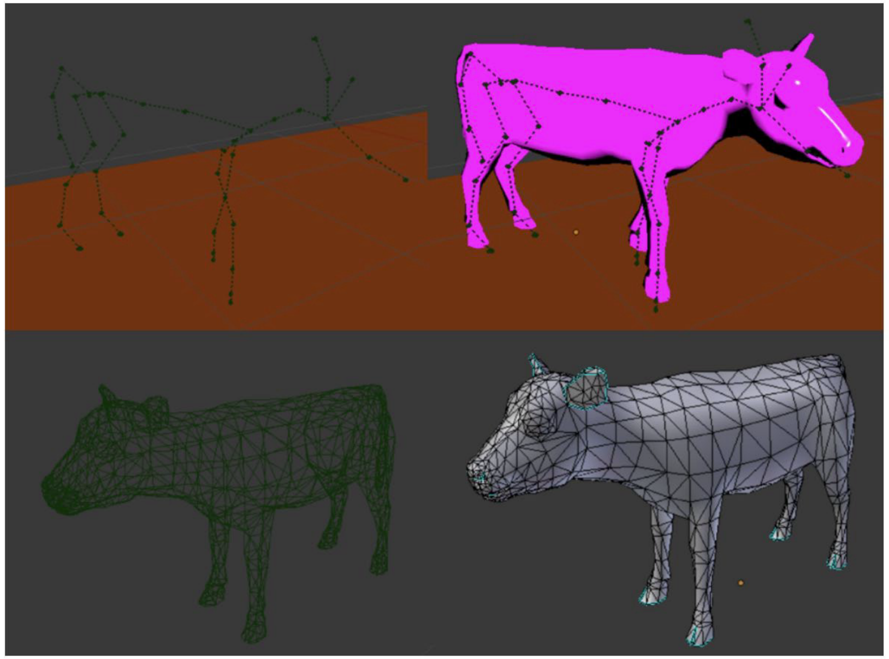

Synthetic Data

Artificial Intelligence and Machine Learning offers the potential to provide objectification of measures that were previously subjective

- Emotions and feelings
- Replacement, reduction, and refinement
- Technology era

Kansas State University
College of Veterinary Medicine

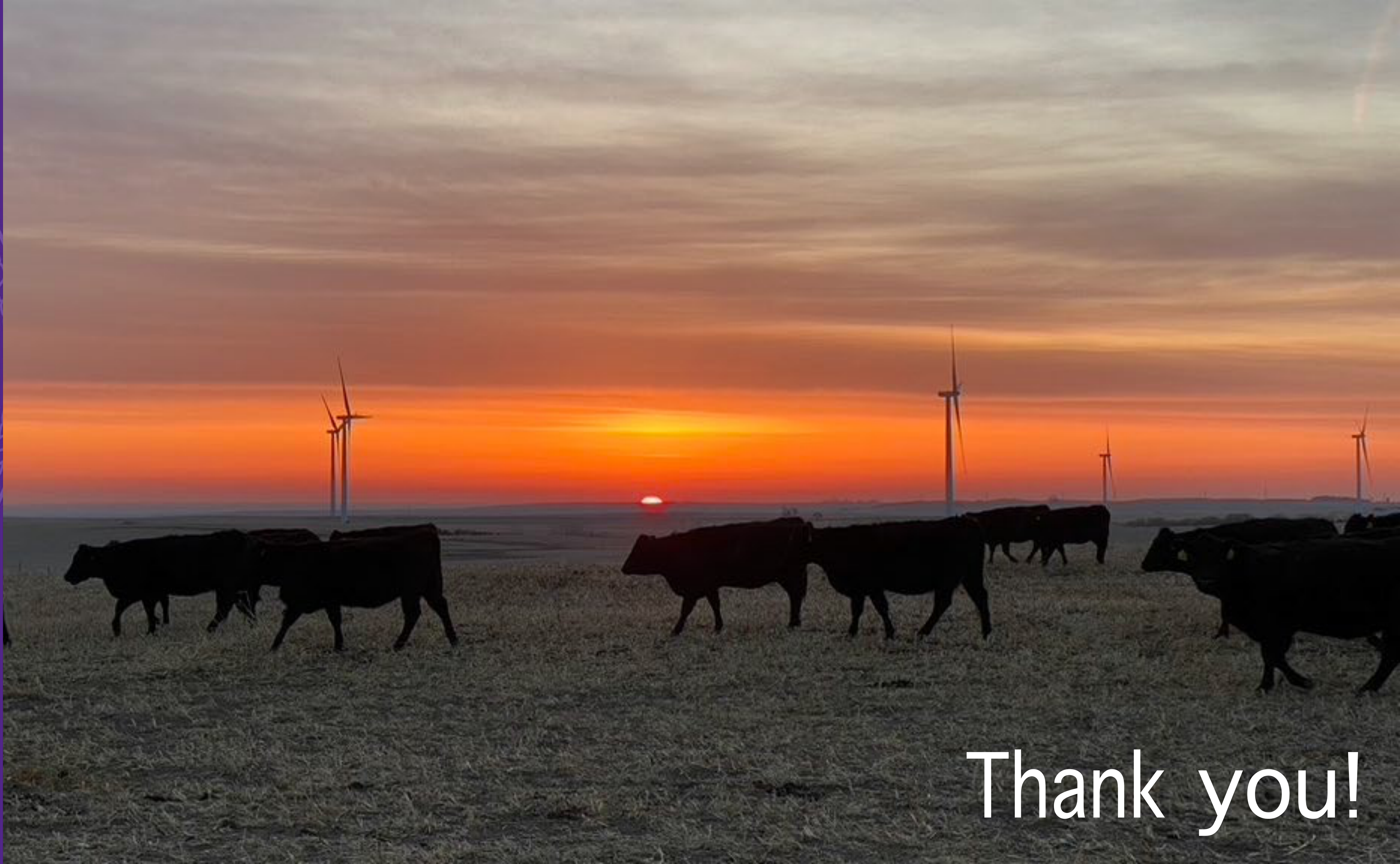

Thank you!
